# High resolution profiling of coral-associated bacterial communities using full-length 16S rRNA sequence data from PacBio SMRT sequencing system

**DOI:** 10.1038/s41598-017-03139-4

**Published:** 2017-06-05

**Authors:** Wirulda Pootakham, Wuttichai Mhuantong, Thippawan Yoocha, Lalita Putchim, Chutima Sonthirod, Chaiwat Naktang, Nalinee Thongtham, Sithichoke Tangphatsornruang

**Affiliations:** 1 National Center for Genetic Engineering and Biotechnology (BIOTEC), National Science and Technology Development Agency, Pathum Thani, Thailand; 2 Phuket Marine Biological Center, Phuket, 83000 Thailand

## Abstract

Coral reefs are a complex ecosystem consisting of coral animals and a vast array of associated symbionts including the dinoflagellate *Symbiodinium*, fungi, viruses and bacteria. Several studies have highlighted the importance of coral-associated bacteria and their fundamental roles in fitness and survival of the host animal. The scleractinian coral *Porites lutea* is one of the dominant reef-builders in the Indo-West Pacific. Currently, very little is known about the composition and structure of bacterial communities across *P. lutea* reefs. The purpose of this study is twofold: to demonstrate the advantages of using PacBio circular consensus sequencing technology in microbial community studies and to investigate the diversity and structure of *P. lutea*-associated microbiome in the Indo-Pacific. This is the first metagenomic study of marine environmental samples that utilises the PacBio sequencing system to capture full-length 16S rRNA sequences. We observed geographically distinct coral-associated microbial profiles between samples from the Gulf of Thailand and Andaman Sea. Despite the geographical and environmental impacts on the coral-host interactions, we identified a conserved community of bacteria that were present consistently across diverse reef habitats. Finally, we demonstrated the superior performance of full-length 16S rRNA sequences in resolving taxonomic uncertainty of coral associates at the species level.

## Introduction

Coral reefs are among the most productive and biologically diverse marine ecosystems, harbouring approximately 30% of the known aquatic species and supporting the productivity of ~25% of marine fisheries^1^. Corals live with a dynamic and complex assemblage of associated microorganisms including the dinoflagellate alga *Symbiodinium*, fungi, viruses and bacteria that are often considered to be a distinct ecological unit, or “holobiont”^2, 3^. Recent research studies have demonstrated that corals harbour large, diverse populations of bacteria that have co-evolved with their hosts^4, 5^. These prokaryotes are likely to serve beneficial roles in provisioning and cycling of carbon, nitrogen and sulfur in coral reefs^6–10^. Additionally, these coral-associated bacteria appear to be involved in protecting their hosts against pathogenic microbes by preventing their colonisation through physical occupation of otherwise available niches^11^ or through the production of antibacterial compounds^12^. Despite the importance of bacterial associates to coral hosts, there have been very few studies that focused on the identification and characterisation of species-specific association between corals and bacteria.

Historically, environmental 16S rRNA gene profiling was performed using clone-based Sanger sequencing, which provided accurate, full-length or near full-length sequences. Due to the high cost and low-throughput nature of the approach, the number of 16S rRNA sequences used in Sanger-based bacterial profiling studies was often lower than 200 sequences per sample, which was hardly sufficient to capture the complete diversity of the population^13–15^. Over the past decade, the use of Sanger sequencing in bacterial community surveys has gradually been replaced by various next generation sequencing platforms (Roche 454^16^, Illumina^17^, Ion Torrent PGM^18^) due to their economy of scale and magnitude orders higher sequencing throughput. Nowadays, the majority of microbial profiling studies utilises the short-read V3-V4, V4-V5 or V5-V6 amplicons instead of the full-length 16S rRNA sequences in environmental community surveys^19, 20^. The advance in throughput has, however, come at the cost of read length, and this tradeoff has inevitably resulted in less accurate classification of partial 16S sequences, especially at the genus or species level. Previous studies have shown that taxonomic assignment and phylogenetic placement were highly sensitive to the region of the 16S rRNA gene sequenced as well as the length of the region sequenced^21, 22^.

Recently, Pacific Biosciences (PacBio) has developed a single molecule real time (SMRT) DNA sequencing system that is able to generate raw reads with an average length of longer than 10 kb. Even though these raw reads are inherently error-prone, with an average error rate of 13%^23^, the multi-pass nature of circular consensus sequencing (CCS) allows calling of the consensus insert sequences with the accuracy of >99%^24^. The use of barcodes enables multiplexing of different samples into a single SMRTbell library, reducing the overall sequencing cost. Despite the fact that PacBio platform can provide full-length 16S rRNA sequence data at a fraction of the cost of Sanger sequencing, it is still not as cost-effective as the short-read Illumina or Ion Torrent technologies. This may be one of the reasons why PacBio system has not widely been adopted in microbial community surveys^25, 26^. Here, we demonstrated the advantages of the long-read PacBio sequencing technology by comparing the ability of full-length and partial 16S rRNA sequences in classifying coral-associated bacteria at the species level.

Southeast Asia contains the largest area of coral reefs with 34% of world’s total^27^. The scleractinian coral *Porites lutea* is one of the dominant reef-builders widely distributed across the Indo-West Pacific, including the Gulf of Thailand and Andaman Sea^28^. A number of reports have highlighted the important contribution of coral-associated bacteria to the overall fitness and long-term survival of their host colonies^12^. Currently, very little is known about the composition and structure of bacterial communities across *P. lutea* reefs. Our objective here is twofold; first to demonstrate the feasibility and practicality of utilising PacBio CCS technology in microbial community studies, and second to investigate the diversity and structure of bacterial communities associated with *P. lutea*. This is the first 16S rRNA gene-based community survey of marine environmental samples that takes full advantage of the PacBio SMRT sequencing system to capture full-length 16S rRNA sequences. With tens of thousands of full-length 16S reads obtained from each sample, we thoroughly examined the core microbiome harboured by *P. lutea* and compared the diversity and composition of bacterial communities associated with corals from the Gulf of Thailand and Andaman Sea.

## Results and Discussion

### Multiplex amplicon sequencing of full-length 16S rRNA gene

**Figure 1:**
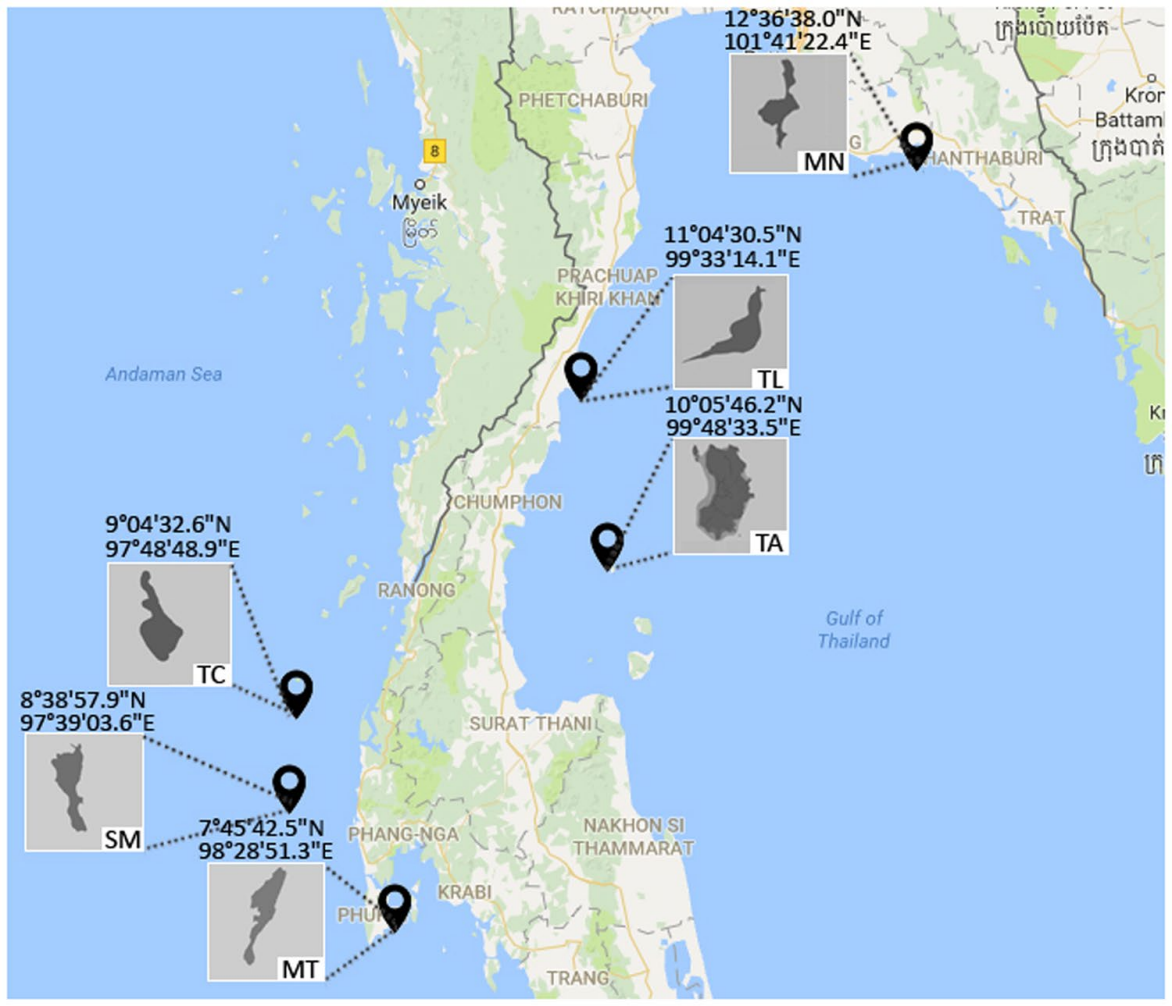
A map showing the sampling sites (with GPS coordinates) in the Gulf of Thailand and Andaman Sea. Sampling locations are abbreviated as follows: Mannai (MN), Talu (TL), Tao (TA), Tachai (TC), Similan (SM) and Maiton (MT). The main and inset maps were drawn using the R packages^83^ ‘ggplot2’ version 2.2.1 (https://cran.r-project.org/web/packages/ggplot2/ggplot2.pdf)^84^ and ‘ggmap’ version 2.6.1 (https://cran.r-project.org/web/packages/ggmap/ggmap.pdf)^85^. The static map of the Gulf of Thailand and Andaman Sea displayed was queried from the Google Maps (Map data^©^2016 Google) using the function ‘get_map’ in the ‘ggmap’ package.

**Figure 2:**
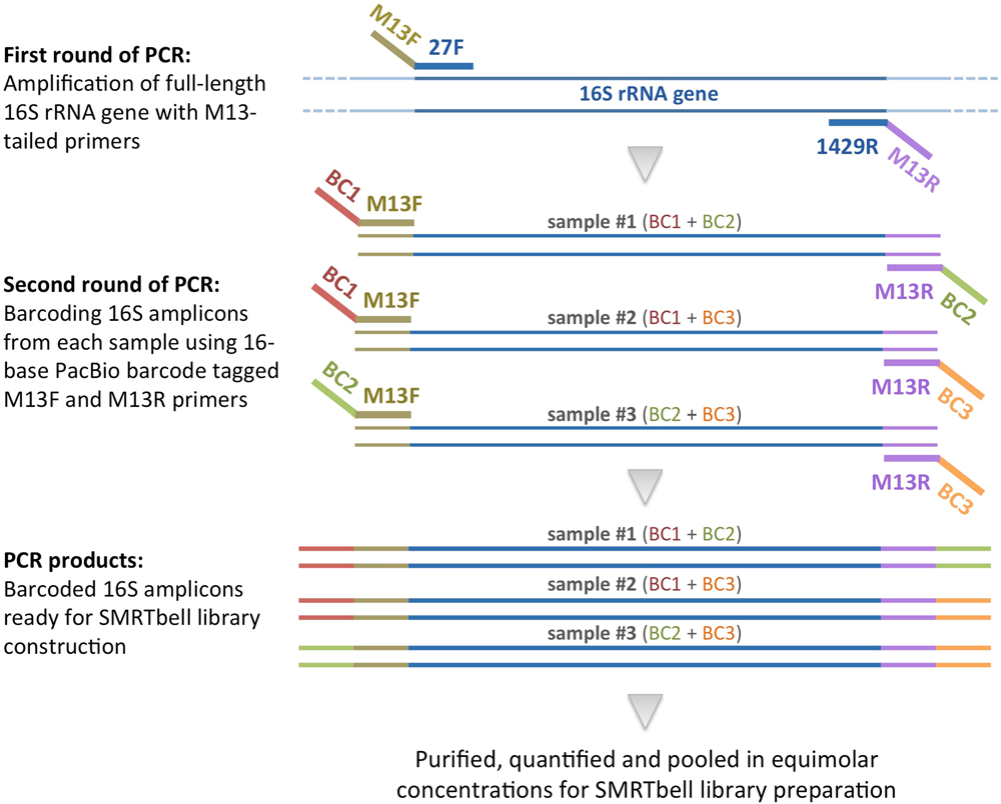
Two-step generation of 16S amplicons for PacBio sequencing using M13-tagged barcoded primers. The first round of PCR amplifies full-length 16S rRNA fragments using gene-specific primers tailed with universal M13 forward or reverse sequence. In the second step, a unique combination of barcode sequences is added to 16S amplicons from each sample using M13 forward and reverse primers tagged with 16-base PacBio barcodes at their 5′ ends. Barcoded amplicons are subsequently pooled for SMRTbell library construction and multiplexed sequencing.

Full-length 16S rRNA genes were amplified from bacterial communities associated with *P. lutea* collected from six sampling sites in the Gulf of Thailand and Andaman Sea (Fig. 1). Three coral samples were collected at each site. Barcoded full-length 16S rRNA amplicons were generated using a two-step PCR approach. The first round of PCR was performed using 16S specific primers concatenated with universal M13 forward or reverse sequences at their 5′ ends. Unique combinations of identifier sequences (barcodes) were added to primary PCR products during the second round of amplification using M13 forward and reverse primers tagged with 16-base PacBio barcode sequences. We decided to use an asymmetric barcoding system where PCR products from each sample were tagged with completely different barcode sequences on each end (Fig. 2). This mix-and-match barcoding strategy can achieve a high level of multiplexing with fewer number of barcode-tagged M13 forward and reverse primers and thus helps minimize the cost of primer synthesis and HPLC purification, which can be substantial in surveys with a large number of samples.

A total of 1,187,995 PacBio reads totaling 28.15 Gb were obtained from 24 SMRT cells (12 SMRT cells/library) with a mean accuracy of 84%. We were able to assemble and demultiplex 606,795 circular consensus sequencing (CCS) reads with an average accuracy of 99.36% and a mean length of 1,402 nt (Supplementary Fig. S1). The average polymerase read length was 25.4 kb, and the average number of full passes of the CCS reads was 21.2. Following the removal of chimeras and CCS reads shorter than 1000 nt, a total of 571,710 reads were obtained for all samples. The number of processed full-length 16S rRNA sequences per coral sample ranged from 20,453 to 60,143, with an average of 31,761 reads/sample (Table 1).

**Table 1:** Number of sequences, OTUs and alpha diversity estimates of bacterial communities associated with *P. lutea* in the Gulf of Thailand and Andaman Sea.

	Sampling site	Sample name	Number of CCS reads	Number of processed reads*	Number of OTUs	Chao1	Simpson	Shannon	Good’s coverage
**Gulf of Thailand**	Mannai	MN1	44117	30890	1282	1442.30	0.91	5.87	0.99
		MN2	26018	20453	958	1135.77	0.92	5.34	0.98
		MN3	24280	22707	517	702.11	0.83	4.49	0.99
	Tao	TA1	46466	44796	615	776.29	0.95	5.56	1.00
		TA2	24445	22974	523	637.18	0.96	5.98	0.99
		TA3	38438	35603	569	850.35	0.95	5.46	0.99
	Talu	TL1	25792	25562	331	464.17	0.93	5.14	1.00
		TL2	23307	23120	365	514.53	0.93	5.04	0.99
		TL3	25497	24709	367	468.27	0.90	4.91	1.00
**Andaman Sea**	Maiton	MT1	44083	43913	302	482.00	0.77	3.30	1.00
		MT2	41612	41384	285	426.22	0.68	2.39	1.00
		MT3	60478	60143	456	690.37	0.76	3.27	1.00
	Similan	SM1	45338	44254	410	616.90	0.78	2.87	1.00
		SM2	27841	26307	477	621.07	0.87	4.15	0.99
		SM3	33789	32179	282	453.27	0.75	2.48	1.00
	Tachai	TC1	25103	24331	471	624.01	0.88	4.59	0.99
		TC2	24009	23374	288	509.69	0.83	3.34	0.99
		TC3	26182	25011	401	529.18	0.76	3.47	0.99
	**Total:**	606795	571710						

*After the removal of chimeras and reads shorter than 1000 nt.

Due to the high cost of Sanger sequencing, the number of full-length 16S rRNA sequences investigated in coral microbiome studies typically ranged from 50 to 200 reads per sample^14–16, 29^, which was insufficient to capture the complete diversity of the population. This work is among the first to utilise the high-throughput, long-read sequencing capability of the PacBio system to obtain tens of thousands full-length 16S sequences for microbial diversity studies. Even though PacBio sequencing is not as cost-effective as some of the available short-read platforms such as Illumina, it is still much more economical to obtain full-length 16S rRNA gene sequences using PacBio technology compared to the alternative Sanger sequencing. A major drawback of PacBio sequencing approach is the high raw read error rate (~13%)^23^; however, the errors appeared to be distributed randomly throughout the read without any GC-bias^30^. Since CCS allows for repeated sequencing of individual inserts, stochastic errors associated with single-pass sequences are reduced with each CCS pass. The lack of systematic errors in PacBio raw reads facilitates the calling of consensus insert sequences from multi-pass reads with an average accuracy of >99%^31^. With an average polymerase read length of 25.4 kb, the 1.4-kb consensus sequences obtained from multiple passes had an average error rate of only 0.64%, comparable to the rate observed from short-read sequencing platforms^32^.

As the cost of library construction increases linearly with the number of samples, it is more economical to multiplex amplicons from several samples into a single SMRTbell library. Since primer oligonucleotides need to be HPLC-purified after the synthesis to ensure their integrity, it can be expensive to synthesize barcode-tagged gene-specific primers for each target sequence. We demonstrated a simple and cost-effective two-step PCR approach to incorporate barcode sequences into the 5′ and 3′ ends of 16S rRNA amplicons (Fig. 2). After amplifying the target sequence with gene-specific primers tagged with M13F and M13R universal sequences, we added the identifier sequences to the primary amplicons through the second round of PCR using barcode-tagged M13F and M13R primers. This same set of barcode-tagged universal M13 primers can be used to incorporate identifier sequences into other target amplicons that contain M13F and M13R tail sequences at their 5′ and 3′ ends, respectively. Our rationale behind the development of this PacBio barcoding strategy was that research groups are often interested in studying more than one target region in the samples. Synthesis and HPLC purification of a large number of gene-specific fusion primers can be prohibitively expensive especially when a study includes several samples and different gene targets are analyzed. The economical aspect of the amplicon barcoding using a two-step PCR outweighs the simplicity of the fusion PCR method where each gene-specific primer is directly tagged with barcode sequences. Moreover, a single-step PCR with long fusion primers can lead to differences in amplification efficiency among samples; this problem can be alleviated to some extent with a two-step PCR^33^. In an attempt to minimize biases introduced during PCR, the smallest possible number of amplification cycles (i.e., until PCR product bands were barely visible on agarose gels) was chosen for both primary and secondary reactions.

### Assessment of *P. lutea*-associated bacterial diversity using high-coverage PacBio 16S rRNA gene sequences

To assess the diversity of bacterial community present within each sample, a series of alpha diversity indices were calculated. The number of operational taxonomic units (OTUs) at a 3% dissimilarity level and the diversity estimates are listed in Table 1. The highest number of OTUs was found in MN1 sample (1282 OTUs) in the Gulf of Thailand, while the lowest number of OTUs was found in SM3 sample (282 OTUs) collected from the Andaman Sea. Rarefaction curves nearly plateaued off for the majority of the samples (Supplementary Fig. S2), indicating that sufficient sampling has been performed to capture the total diversity of the communities. In addition, Good’s coverage values were above 0.98 for all samples (Table 1), agreeing with the rarefaction analysis result that overall bacterial diversity had likely been observed in these samples.

*P. lutea* corals from different locations harboured bacterial communities with different degrees of diversity, but in general bacterial assemblages from corals in the Gulf of Thailand were more diverse than those in the Andaman Sea (Table 1, Supplementary Fig. S3). Shannon indices ranged from 4.49 to 5.98 for samples collected from the Gulf of Thailand (MN, TA and TL), similar to those reported for *P. lutea* from the South China Sea reefs^34^, whereas samples from the Andaman Sea (MT, SM and TC) had slightly lower index values (2.39–4.59) comparable to those observed for the same coral species from reefs in the Indo-Pacific (Indonesia)^35^ and West Indian Ocean^36^. The Chao1 estimator was employed to calculate estimated richness at the species level and ranged from 426 (MT2) to 1442 (MN1). These richness values were within the ranges of previously reported richness estimates for *Porites*-associated bacterial communities^34, 35^. The differences in the diversity estimates observed among different studies could reflect distinction among geographical locations of the coral hosts, or they could be due to variations in methodologies used to obtain 16S amplicons (e.g. PCR primer selection or 16S rRNA regions used for analyses).

### Habitat influences on the composition of coral-associated bacterial communities

**Figure 3:**
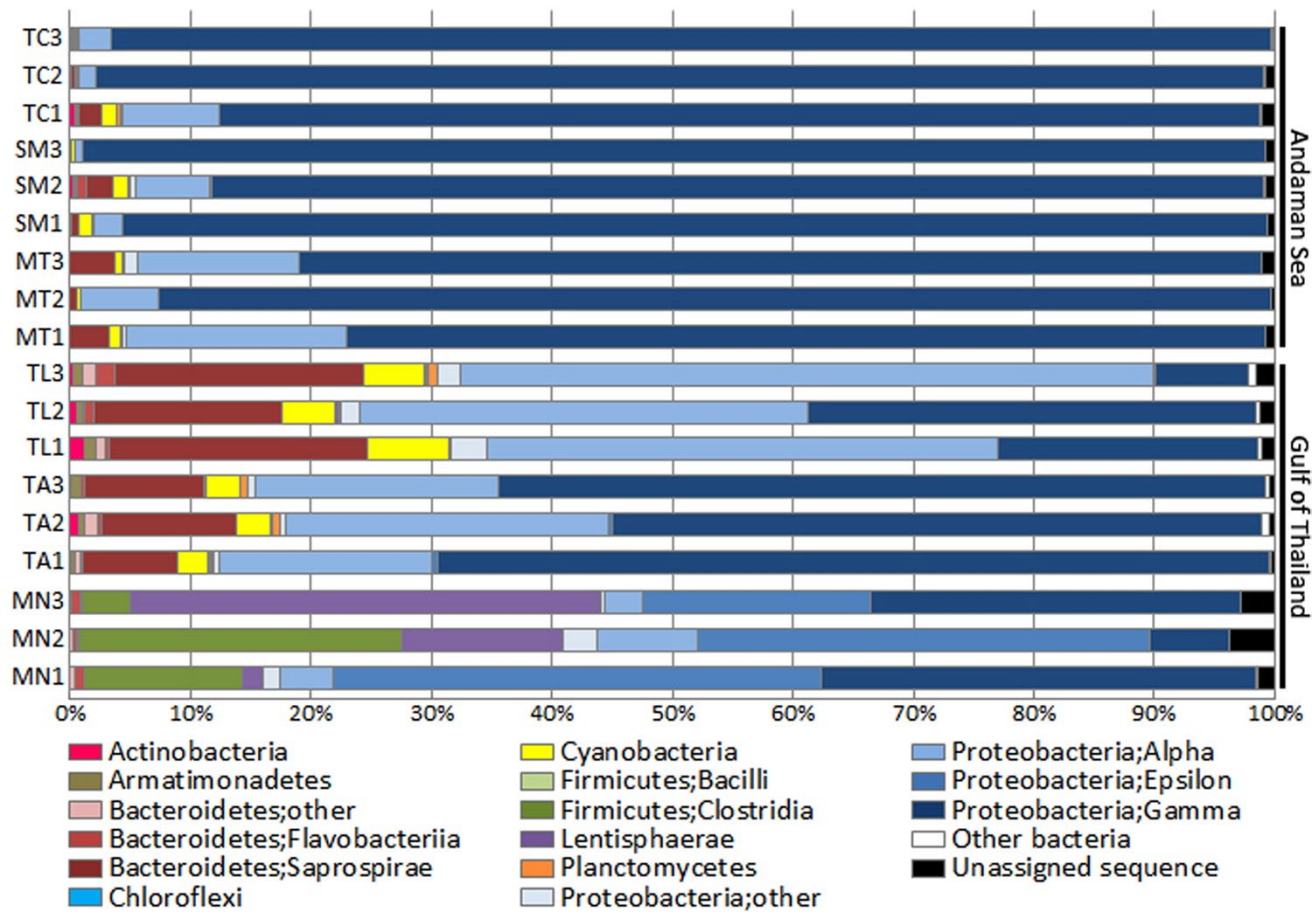
Taxonomic classification of OTUs at the phylum/class levels based on Greengenes database using QIIME software.

Using a confidence threshold of 80%, 552,908 out of 571,710 filtered reads were assigned using the RDP classifiers^22^. Sequences were classified into 18 phyla, 45 classes and 91 orders, and only 1.1% of sequences were unclassifiable at the genus level. A total of 15 known and 3 candidate bacterial phyla were present across all samples, with 8 to 16 phyla present in each sample (Supplementary Dataset 1). The proportion of each phylum varied among corals from different habitats; nonetheless, Proteobacteria represented the ubiquitous and dominant phylum in all *P. lutea* samples, constituting as much as 84.7% of all 16S sequences in our study (Fig. 3). Microbial communities associated with *Porites* corals also comprised members of other commonly occurring marine and coral bacterial phyla, including Bacteroidetes, Firmicutes, Lentisphaerae, and Cyanobacteria.

Proteobacteria account for the majority of bacterial diversity in most of the marine environment including tropical coral species^5, 37^, temperate and cold-water scleractinians and octocorals^38, 39^. The Proteobacteria detected in *P. lutea* contained representatives from the Alpha-, Beta-, Delta-, Epsilon- and Gamma-classes. Classification and diversity analyses revealed geographically distinct coral-bacteria associations, and a thorough analysis showed a significant and consistent difference in the relative sequence abundance of Proteobacteria classes between samples from the Gulf of Thailand and Andaman Sea.

Gammaproteobacteria were predominant in bacterial communities associated with corals in the Andaman Sea (MT, SM and TC), accounting for more than 76% of the sequences in each sample, whereas Alphaproteobacteria (18–57% in TA and TL samples) and Epsilonproteobacteria (19–41% in MN samples) were harboured primarily by corals retrieved from the Gulf of Thailand (Fig. 3). Betaproteobacteria and Deltaproteobacteria were found at consistently low levels (0.1–2.8%) across all samples. Interestingly, sequences affiliated with Firmicutes, Lentisphaerae, Bacteroidetes and Cyanobacteria were much more prevalent in bacterial communities associated with corals in the Gulf of Thailand compared to those in the Andaman Sea (Fig. 3). Similar to our findings, previous studies have also identified members of Alphaproteobacteria, Gammaproteobacteria, Firmicutes and Bacteroidetes as ubiquitous bacterial associates of *Porites* spp. from the Caribbean Sea^40^, Red Sea^19^, South China Sea^34^ and Indo-Pacific reefs^35^.

**Figure 4:**
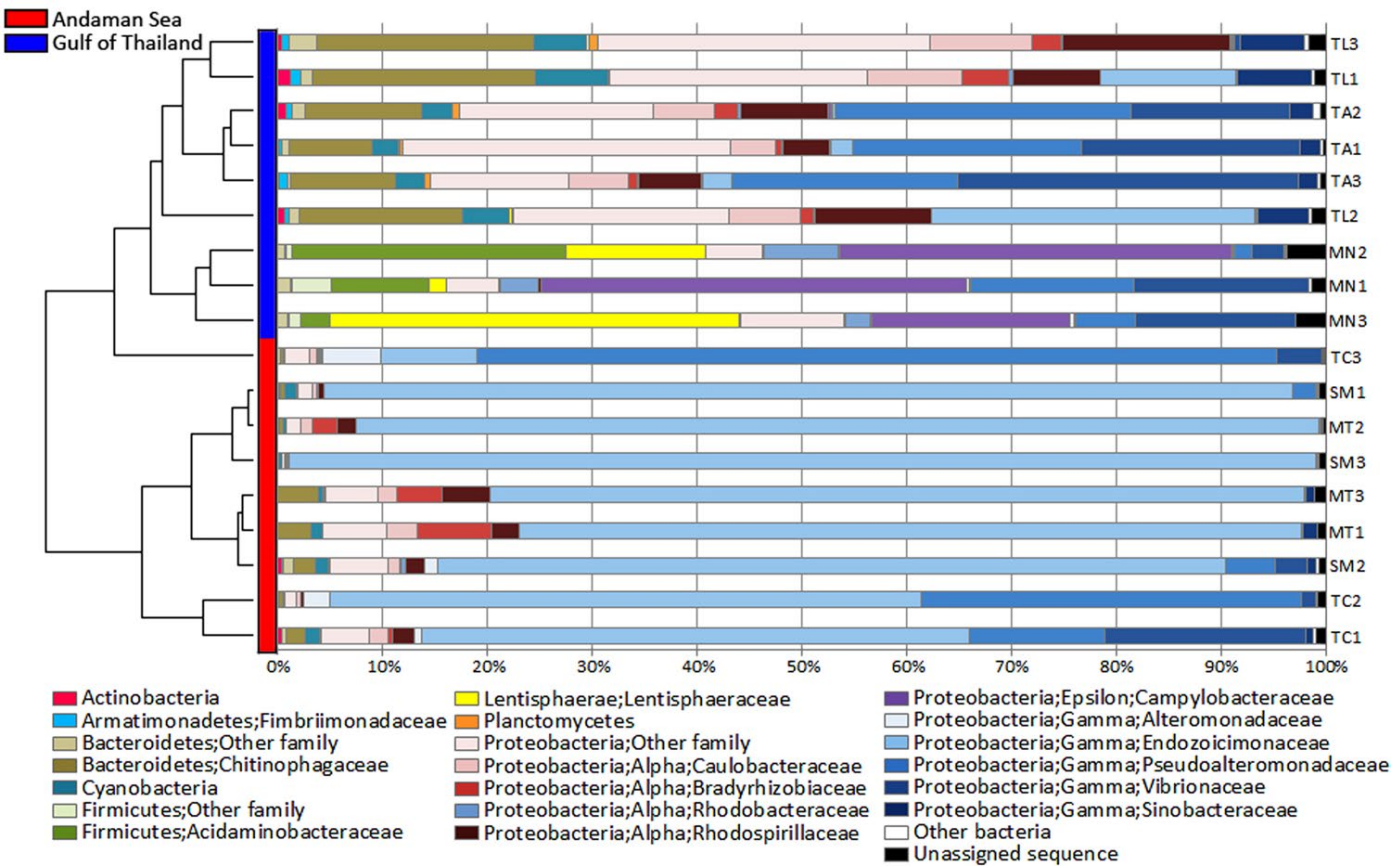
A dendrogram illustrating the similarities among the bacterial profiles of *P. lutea* microbiomes from different locations in the Gulf of Thailand and Andaman Sea.

Unweighted pair group method with arithmetic mean (UPGMA) clustering revealed two significantly distinct clusters of bacterial communities corresponding to the geographical habitats of their coral hosts (Fig. 4). The Gammaproteobacteria orders Oceanospirillales (family Hahellaceae) and Vibrionales (family Pseudoalteromonadaceae) dominated coral-associated bacterial assemblages from the Andaman Sea, with Hahellaceae representing as much as 85% of total reads from MT and SM (Fig. 4). On the contrary, the composition of coral microbiomes was much more diverse in the Gulf of Thailand, with Campylobacterales, Lentisphaerales and Clostridiales detected mostly in samples from MN and Rhodospirillales, Ellin329 and Saprospirales detected mostly in samples from TA and TL (Fig. 4; Supplementary Dataset 1). Pairwise analyses using Welch’s *t*-test (*P-*value < 0.05) highlighted the OTUs primarily responsible for the observed differences between the habitats studied. We noticed a striking overrepresentation of the Gammaproteobacteria family Hahellaceae in coral microbiomes in the Andaman Sea, whereas Chitinophagaceae, Caulobacteraceae and Rhodospirillaceae were more abundant in bacterial communities associated with corals in the Gulf of Thailand (Supplementary Fig. S4).

The Hahellaceae genus *Endozoicomonas* was the predominant taxon for most corals from the Andaman Sea. Dominance of *Endozoicomonas* in *Porites* microbiomes in the Caribbean has previously been demonstrated using various hypervariable regions and sequencing techniques including Illumina sequencing of the V5 regions^41^, 454 sequencing of the V1-V3 regions^42^, and Sanger sequencing of full-length 16S rRNA genes^13^. The specific widespread association of *Endozoicomonas* bacteria with both *P. lutea* and *P. astreoides* may suggest a co-evolution between *Porites* spp. and the symbiotic bacterial partner, as previously suggested for other scleractinian corals *Stylophora pistillata* ^16^ and *Pocillopora verrucosa* ^43^. However, *Endozoicomonas* species have relatively large genomes (5–6 Mb) that do not appear to be streamlined for an obligate endosymbiotic lifestyle, suggesting that they have free-living stages^43, 44^. Analysis of *Endozoicomonas* genomes revealed an enrichment of genes associated with transport and secretion processes, which may be related to the transfer of carbohydrates, amino acids and protein between the symbionts and host cells^45^. Interestingly, *Endozoicomonas* genomes encoded a large number of transposase genes, which may help the bacteria to rapidly adapt to a new host or transition between symbiotic lifestyles^45^.

Geographical locations are known to influence the abundance and composition of bacterial populations within coral microbiomes^46–48^. While corals from most of our sampling sites were associated mainly with Proteobacteria classes Alpha-, Beta-, Delta- and Gammaproteobacteria, samples from MN, situated at the northern tip of the Gulf of Thailand, had a distinct bacterial composition of Epsilonproteobacteria, Lentisphaeria and Clostridia (Fig. 4). The presence of Clostridia in *P. lutea*-associated microbiome has previously been observed in the South China Sea^49^ and the Indo-Pacific^35^, whereas Epsilonproteobacteria and Lentisphaeria have been identified from corals in the West Indian Ocean^36, 50^. Cyanobacteria represented a minor constituent (<5%) of *P. lutea* microbial communities in our study, similar to the reports from the South China Sea^49^ and West Indian Ocean^36^. Conversely, a higher proportion was observed in a microbial survey from the Indonesian reefs, where as much as 28% of the 16S sequences from the assemblages were affiliated with Cyanobacteria^35^. In the South China Sea, Chlorobi was identified as one of the major groups associated with *P. lutea* ^49^, but it was absent from bacterial assemblages associated with reefs in the Indo-Pacific (Indonesia)^35^, Gulf of Thailand and Andaman Sea. Differences in *P. lutea-*associated microbial profiles across the globe suggest that environmental conditions and geographical separation exert certain influences on the composition of bacterial consortia associated with coral hosts. It is also plausible that the inconsistency observed among the findings on the specificity of coral-associated microbial communities was due to differences in the 16S sequencing methods or the choice of primers used. Each sequencing approach has its own drawbacks and biases and thus may have failed to capture a complete picture of the microbial assemblages associated with *P. lutea*. Several studies have shown major differences in community abundance when different primer sets were used, and in many cases, specific bacterial lineages could not be detected with one primer set or the other^51–53^. Therefore, it is entirely possible that the discrepancies in the abundance and composition of *P. lutea*-associated bacterial populations observed between this and past studies may be due to primer choices.

### Bacterial indicator species associated with *P. lutea* from different locations

To determine whether particular bacterial species were significantly associated with corals from different locations in this study, we analysed our data for the presence of candidate indicator species at each sampling site using the indicator value (INDVAL) method^54^. Coral samples from MN appeared to harbour a large number of unique species exclusively found in that location, leading to the identification of several indicator species with an index value of 1. About half of those indicator species were members of Alphaproteobacteria family Rhodobacteraceae (genera *Aliiroseovarius, Leisingera, Roseovarius, Thalassobius* and *Tropicibacter*) while the remaining belonged to a diverse group of bacteria, with representatives from Bacteriodetes (*Marinifilum fragile*, *Tenacibaculum gallaicum*), Clostridia (*Vallitalea guaymasensis*), Deltaproteobacteria (*Halobacteriovorax litoralis*), Epsilonproteobacteria (*Arcobacter defluvii*, *Arcobacter halophilus*) and Gammaproteobacteria (*Litoribrevibacter albus*, *Photobacterium angustum*; Supplementary Table S1). Interestingly, a few indicator species for TA including *Vibrio crassostreae*, which was present exclusively at this location, belonged to the genus that was often associated with coral diseases^55^. Recently, it has been shown that *V. crassostreae* was a benign colonizer of oysters that could be turned into a pathogen by introgression of virulence plasmid into the population^56^. Since *V. crassostreae* was identified as an indicator species for visually healthy colonies in TA, this species was probably not virulent in *P. lutea* at the time of sample collection. While the majority of the indicator species associated with *P. lutea* from TA and TL (Gulf of Thailand) are members of Gammaproteobacteria, it is interesting to note that indicator species for corals from the Andaman Sea, especially those that were exclusive to the sampling sites (i.e., having an index value of 1), usually belonged to the taxa with relatively low abundance such as Betaproteobacteria, Bacteroidetes and Actinobacteria (Supplementary Table S1). It is currently not known whether any of the low abundant community members identified is functionally relevant to coral’s health and survival.

### Identification of the *P. lutea* core microbiome

**Figure 5:**
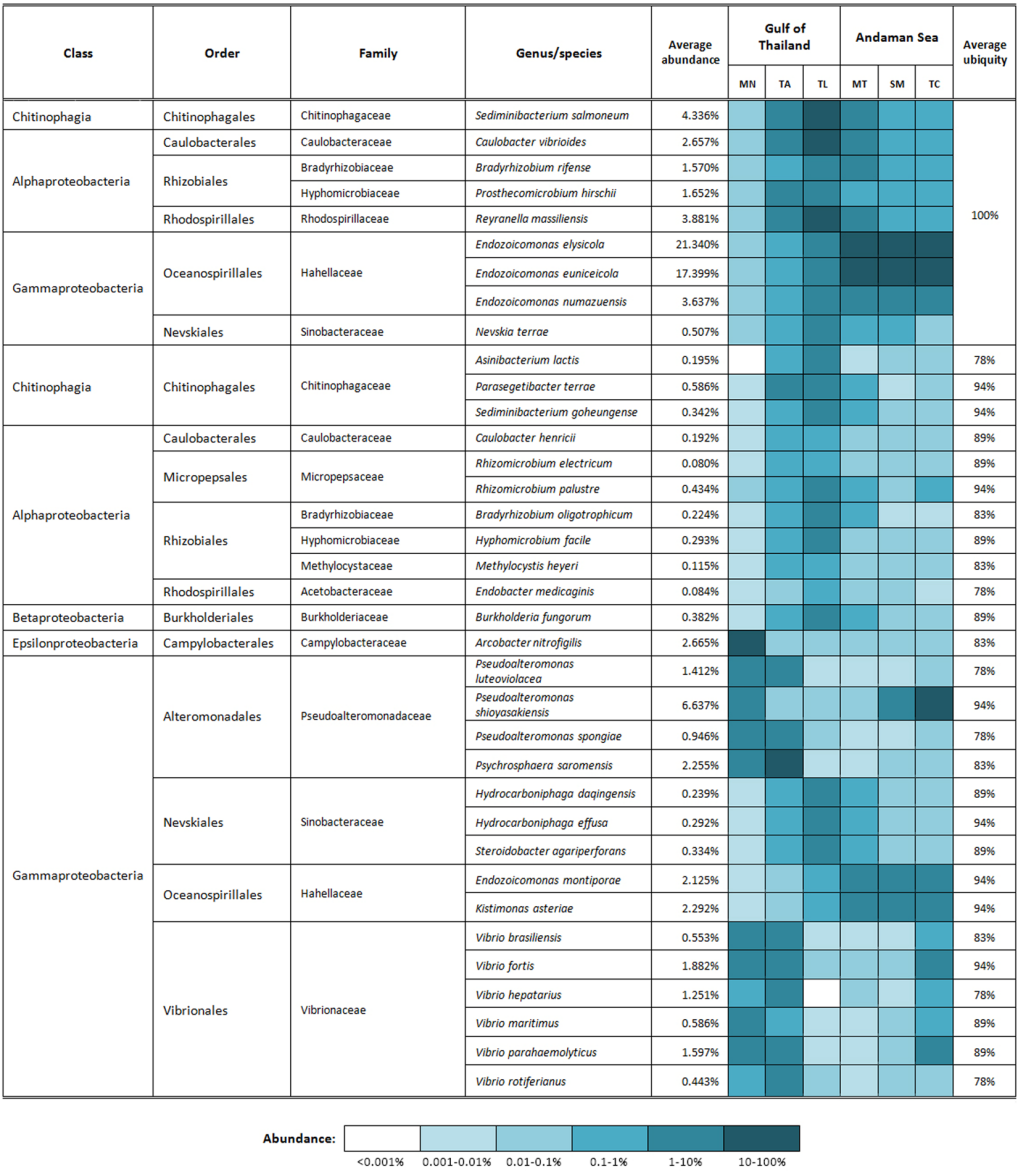
A list of bacteria present in *P*. *lutea* core microbiome, their relative abundance (average species abundance across all 18 *P. lutea* samples) and their average ubiquity (percentages of *P. lutea* samples in which the species was detected). The heat map displays the average abundance of species in the core microbiome from each of the sampling sites in the Gulf of Thailand and Andaman Sea.

Several studies reported that similar bacterial populations have been found on the same coral species that were geographically separated^2, 14, 57^, suggesting that corals harboured certain species-specific microbiome regardless of their origins. Relatively little is known about the bacteria assemblage that constitutes the *P. lutea* core microbiome. To examine the conserved bacterial communities associated with *P. lutea*, we first identified species that were ubiquitously present in at least 50% of all samples disregarding the relative abundance of each species. A total of 98 species were identified as candidate members of the core microbiome, with Gammaproteobacteria and Alphaproteobacteria constituting the greatest proportions of the putative core microbiome (71% and 11.5%, respectively; Supplementary Table S2). Oceanospirillales (47.25%), Alteromonadales (14.7%), Vibrionales (7.65%) orders had the highest relative abundance with 10, 23 and 22 species, respectively, present in the putative core microbiome (Supplementary Table S2). When a more stringent criterion was applied to examine bacterial species consistently present in at least 75% of *P. lutea* samples (defined as core members), we identified 36 conserved bacterial species from 21 genera, the majority of which belonged to Gammaproteobacteria families Vibrionaceae, Hahellaceae, Pseudoalteromonadaceae, and Chitinophagaceae (Fig. 5). We also evaluated our corals for bacterial species that were highly persistently and found in all 18 samples. Nine conserved bacterial species consistently present at 100% sample coverage were members of Alphaproteobacteria (*Caulobacter vibrioides*, *Bradyrhizobium rifense*, *Prosthecomicrobium hirschii, Reyranella massiliensis*), Gammaproteobacteria (*Endozoicomonas elysicola, Endozoicomonas euniceicola, Endozoicomonas numazuensis, Nevskia terrae*) and Bacteroidetes (*Sediminibacterium salmoneum*; Fig. 5).

Hahellaceae have previously been reported as dominant members of the core microbiomes of several coral hosts, including *Acropora cervicornis, P. astreoides* and *P. furcata* ^58^. Bayer *et al*.^59^ analysed *Endozoicomonas* sequences associated with 14 species of corals from various geographic regions and found that none of the *Endozoicomonas* OTUs were detected in more than one coral host, suggesting that each coral harboured its own unique *Endozoicomonas* strain. In this study, we showed that *E. elysicola* and *E. euniceicola* were the two most abundant species in the *P. lutea* core microbiome, with the relative abundance of 21.3% and 17.4%, respectively (Fig. 5). Besides being found embedded deep within tissues of *Stylophora pistillata* and *Pocillopora verrucosa* corals^43^, *Endozoicomonas* appears to inhabit the coral surface mucus layer and the coral skeleton^60^ of both *Acropora* and *Porites* corals, and its function has been speculated to be involved in biofilm production that allowed other bacteria to colonize coral surfaces^61^. It has also been shown that *Endozoicomonas* genomes are enriched for genes involved in transport activities, especially carbon sugar transport and the secretion of proteins, suggesting that *Endozoicomonas* species have potential roles in the upcycling of carbohydrates and the provision of proteins to the host^45^.

Multiple *Vibrio* and *Pseudoalteromonas* species were detected in the *P. lutea* core microbiome, similar to the previous observation on *P. astreoides* ^58^. One of the *Pseudoalteromonas* species present in the core microbiome, *Pseudoalteromonas luteoviolacea*, has been shown to produce compounds with antibacterial activity^62^, suggesting that it may be involved in protecting *P. lutea* against pathogenic microbes. Another member of the core microbiome, *Pseudoalteromonas spongiae*, has been demonstrated to form active biofilm on natural marine substrata and facilitate larval settlement of certain benthic invertebrates^63^. Interestingly, six out of thirty-six species identified as members of the *P. lutea* core microbiome belong to the genus *Vibrio* (Fig. 5). *Vibrio* species are often acknowledged for their roles as opportunistic or pathogenic bacteria associated with coral diseases^64, 65^; nevertheless, they are also recognized as common members of healthy coral microbiomes^5, 7, 60^. Nitrogen fixation has been demonstrated as a common characteristic among several *Vibrio* species, including putative coral pathogenic strains such as *Vibrio alginolyticus* and *Vibrio harveyi* ^66^. It is plausible that under normal condition these *Vibrio* species develop a mutualistic relationship with corals, and upon nutrient or heat stress (for example, high sea water temperature), they act as opportunistic pathogens, outcompeting other species in the coral mucus^61^. To this point, there has been no report linking any of the *Vibrio* species present in the *P. lutea* core microbiome (*Vibrio brasiliensis, Vibrio fortis, Vibrio hepatarius, Vibrio maritimus, Vibrio parahaemolyticus, Vibrio rotiferianus*) to coral diseases. However, four *Vibrio* isolates identified as a causative agent of yellow blotch/band disease in reef-building Caribbean corals *Montastraea* were closely related to *V. parahaemolyticus* based on 16S rRNA sequence homology^67^. Despite the lack of evidence of *V*. *parahaemolyticus* causing diseases in corals, this particular Gram-negative bacterium is a major food-borne pathogen that causes life-threatening diseases in humans following the consumption of raw or undercooked marine products through the production of various toxins and virulence factors such as hemolysins^68^. Future studies are required to determine whether *V. parahaemolyticus* is capable of inducing signs of yellow blotch/band or other diseases in corals and to examine possible roles of those toxins in coral infection.

We noticed that most of the species identified in the *P. lutea* core microbiome with representation in at least 75% of the samples were present in low abundance. Of the 36 species found within at least 75% of all samples, only three had a relative abundance higher than 5% within the whole community. Another 14 had a relative abundance between 1% to 5%, and the majority was found to have lower than 1% relative abundance in the whole community analysis (Fig. 5). Similarly, in acroporid coral *Acropora granulosa*, only two (out of 159 phylotypes detected within at least 30% of samples) were found to have higher than 5% relative abundance within their bacterial-associated communities^60^. These findings highlight the need for caution when determining bacterial associates as core members of the microbiome based solely on a high relative abundance within the host’s whole community^60, 69^. Interestingly, while Littman *et al*.^70^ demonstrated that bacterial profiles separated *Acropora* corals based on geographic locations, their conclusions were based primarily on dominant members of the community driving the differences between locations. Upon careful examination of individual sequences that represented minor components of retrieved 16S sequences, the authors indeed noticed the same bacterial phylotypes within all corals investigated. This is in agreement with our finding that bacteria that form stable and species-specific associations may be present at low relative abundance in the coral microbiome.

### Superior performance of full-length 16S rRNA sequences in taxonomical classification at species resolution

**Figure 6:**
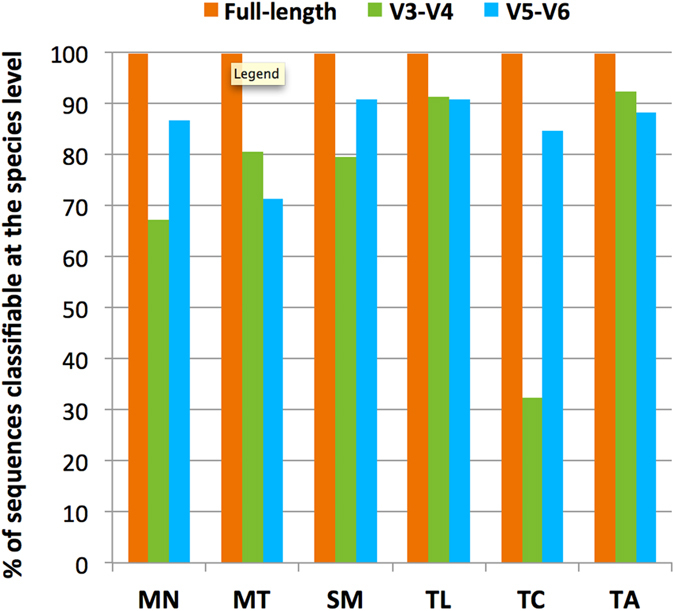
A histogram illustrating proportions of sequence reads from each sampling site that are classifiable at the species level using full-length (orange) or partial 16S rRNA gene fragments (V3-V4, green; V5-V6, blue).

Resolving the taxonomy of 16S rRNA sequences based solely on limited segments of the 16S rRNA gene derived from next generation sequencing can be challenging. To compare the resolution of microbial community analyses obtained from commonly used hypervariable regions and full-length 16S rRNA sequences, we first extracted the V3-V4 and V5-V6 regions from the full-length reads and aligned both the *in silico* amplicons (420-bp V3-V4 and 255-bp V5-V6 fragments) and their respective full-length sequences against non-redundant reference sequences in the Ribosomal Database Project (RDP)^71^. We observed notable differences in the proportions of sequences that could be assigned at the species level using V3-V4, V5-V6 hypervariable regions or full-length amplicons (Fig. 6). Notably, 99.7% of the full-length 16S sequences were taxonomically classified at the species resolution, whereas the percentages of the V3-V4 sequences assigned to specific species varied from 32% to 93%, depending on the taxa and composition of microbial associates present in the samples. Even though the percentages of the V5-V6 segments effective for taxonomic identification (71% to 90%) were lower than those of the full-length sequences (99.7%), the overall performance of this hypervariable region in species identification of *P. lutea*-associated bacteria appeared to be better than that of the V3-V4 sequences (Fig. 6).

**Figure 7:**
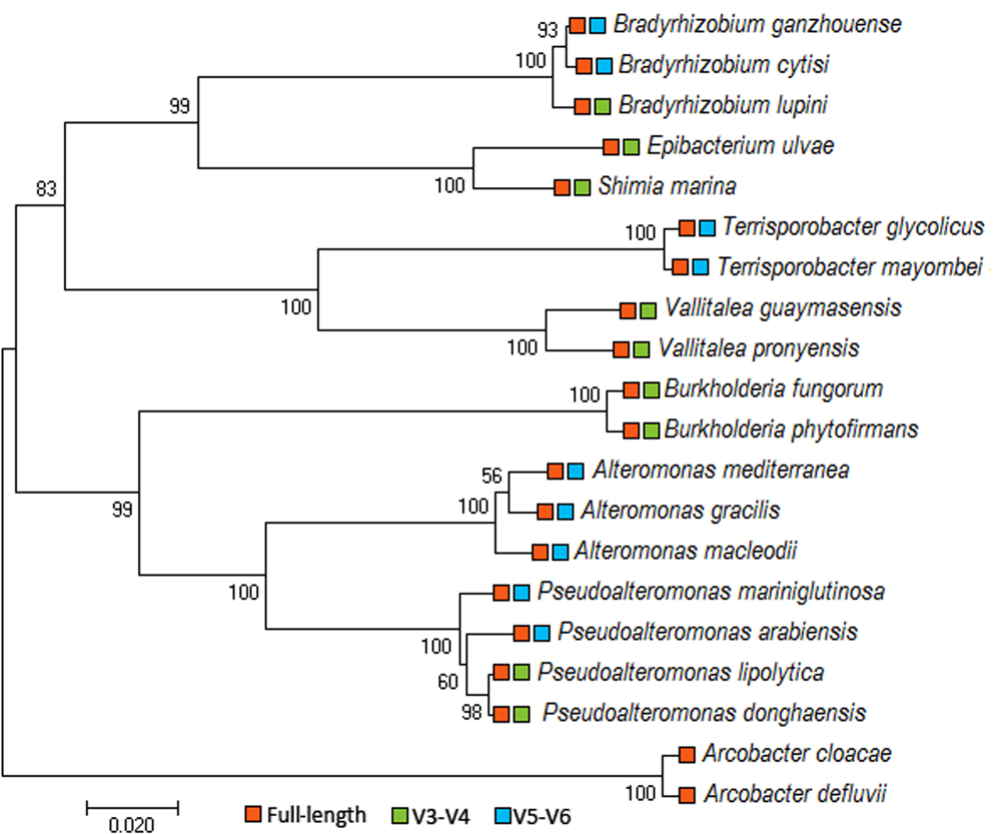
Comparison of species detection between full-length and partial 16S rRNA sequences. A neighbor-joining phylogenetic tree of 20 species identified in coral-associated microbiome generated using MEGA7. Reference type strain sequences were obtained from the RDP database. The clustering of the sequences was tested by a bootstrap approach using 1,000 replications. Orange, green and blue boxes indicate species that are classifiable based on sequence information from full-length, V3-V4 and V5-V6 regions of the 16S rRNA genes, respectively.

Upon further investigation, we found several groups of CCS reads, the V3-V4 and/or V5-V6 regions of which could not be used to identify taxa at a high resolution. These groups contained sequences affiliated with bacteria from genera *Alteromonas, Pseudoalteromonas*, *Burkholderia, Terrisporobacter, Arcobacter*, *Vallitalea* and *Bradyrhizobium* among others (Fig. 7 and Supplementary Table S3). Specific examples of taxonomic classification at the species level that could not be achieved based on the sequences of V3-V4 and/or V5-V6 regions alone are illustrated in Fig. 7 and Supplementary Fig. S5. Interestingly, we also observed a situation in which two bacterial species (*Epibacterium ulvae* and *Shimia marina*) belonging to different genera could not be taxonomically separated based solely on the sequence information from their respective V5-V6 regions (Fig. 7 and Supplementary Fig. S5). The number and percentage of bacterial species within each genus (identified in our coral microbiome) that were classifiable using the V3-V4, V5-V6 or full-length 16S rRNA sequences are shown in Supplementary Table S3. It is worth noting that the performance comparison reported here was based on *in silico*-amplified V3-V4 and V5-V6 fragments, which only considered the classification power of the sequences in those regions. However, it did not take into account the priming bias of the primers, which was one of the important factors responsible for differences in community profiles evaluated using different primer sets^52^.

An unusually low proportion (32.33%) of total sequences from TC could be classified at the species level using the V3-V4 sequences (Fig. 6), prompting us to examine those unclassified reads more closely. We discovered that almost 60% of the CCS reads from TC belonged to either *Pseudoalteromonas arabiensis* or *Pseudoalteromonas mariniglutinosa*. The V3-V4 variable regions of these two bacterial species shared 100% sequence identity and thus could not be used for classification at species resolution (Fig. 7, Supplementary Fig. S5). While the sequence information from V5-V6 fragments were effective in differentiating *Pseudoalteromonas arabiensis* from *Pseudoalteromonas mariniglutinosa*, it is interesting to note that it could not distinguish other two *Pseudoalteromonas* species, namely *Pseudoalteromonas donghaensis* and *Pseudoalteromonas lipolytica* (Fig. 7, Supplementary Fig. S5). A similar case was observed with sequences associated with genus *Bradyrhizobium*, where the V3-V4 regions could classify *Bradyrhizobium lupini* but not *Bradyrhizobium ganzhouense* or *Bradyrhizobium cytisi*; however, the situation was reversed when the V5-V6 sequences were used for the classification (Fig. 7).

These findings demonstrated that partial 16S sequence information from the V3-V4 or V5-V6 regions alone is often insufficient for taxonomic assignments of the coral-associated bacteria at the species level. In many cases, the sequence to be classified is identical or nearly identical to several other bacterial sequences in the reference database. In contrast, full-length 16S sequences had an advantage of covering all hypervariable regions of the 16S rRNA genes and could provide higher taxonomic resolution compared to partial 16S sequences. Ambiguous classification or misclassification of short hypervariable sequences also affects the interpretation of community function inferred from community diversity information at different taxonomic levels^72^. In addition to providing correct taxonomic assignment, full-length 16S rRNA gene sequences yielded more accurate estimates of species richness compared to short hypervariable fragments obtained from next generation sequencing^73^. Youssef *et al*.^73^ evaluated the validity of species richness estimates calculated using pyrosequencing-derived fragments and their correlations to estimates produced using nearly complete 16S rRNA sequences and demonstrated that divergent estimates of OTUs and species richness could be over- or underestimated depending on the hypervariable regions used. The bias in species richness estimates observed could be explained by the proportions of hypervariable, variable and conserved bases present in the fragments used in the analyses^73^.

Recent advances in high-throughput PacBio sequencing and its ability to generate CCS reads with higher than 99% accuracy have provided an economical approach to investigate the diversity and structure of microbial communities in environmental samples using full-length 16S rRNA sequences. The utilisation of full-length sequence information will help mitigate issues relating to classification discrepancies/misclassification and the inflation of diversity or missing diversity. Finally, the generation of full-length 16S rRNA sequences will also help the research community expand the microbial 16S rRNA gene catalogue as new full-length sequences from underrepresented or previously undiscovered taxa can be deposited into the reference databases. A resurgence of full-length 16S rRNA sequences used as gold standards, through the adoption of the PacBio CCS technology, has the potential to transform microbial community profiling studies by increasing the accuracy of taxonomic assignments for both known and novel species.

## Materials and Methods

### Sample collection and DNA extraction

Coral samples were collected in February 2016 at the depth of 7–12 m from six survey sites in the Gulf of Thailand (Mannai, MN; Tao, TA; Talu, TL) and Andaman Sea (Maiton, MT; Similan, SM; Tachai, TC; Fig. 1). At each sampling location, samples of *P. lutea* were collected underwater from three visually healthy colonies that were at least 10 m apart using scalpel blades and placed in sterile disposable 2-mL screw-capped tubes (without any air space). Samples from all locations were transported back to shore within 2–3 hours under low light in a 4 °C container. Upon returning to the laboratory, seawater was immediately removed from each tube, and coral samples were completely submerged in absolute ethanol and stored at −20 °C prior to DNA extraction.

Coral tissue samples were pulverized in liquid nitrogen with sterile mortars and pestles, and genomic DNA was extracted using the High Pure Template PCR Preparation Kit (Roche Life Science, Indianapolis, IN, USA) according to the manufacturer’s instruction. DNA was eluted in 50 μL of elution buffer and its quality was assessed on 0.8% agarose gel to ensure there was no degradation or ribosomal RNA contamination. DNA samples were subsequently quantified using the NanoDrop ND-1000 Spectrophotometer (Thermo Fisher Scientific, Waltham, MA, USA) and diluted to 50 ng/μL for PCR amplification.

### 16S rRNA gene amplification, sample barcoding and PacBio sequencing

The diversity of bacterial communities associated with corals from various habitats was analyzed using single molecule real-time PacBio sequencing technology (Pacific Biosciences, Menlo Park, CA, USA). Full-length 16S ribosomal RNA gene was amplified from 50 ng of genomic DNA using the bacterial-specific primer 27 F (5′-AGAGTTTGATCMTGGCTCAG) and 1492 R (5′-TACGGYTACCTTGTTACGACTT). To allow multiplexing of amplicons from several samples in one library, the 5′ ends of the 16S rRNA forward and reverse primers were tagged with the universal M13F (5′-TGTAAAACGACGGCCAGT) and M13R (5′-GGAAACAGCTATGACCATG) sequences, respectively. In addition to universal sequence tails, a 5′ block (5′NH_4_-C6) was added to 16S specific primers to ensure that carry-over amplicons from first round PCR were not ligated to the SMRTbell adapters in subsequent steps. A set of five barcoded M13F and five barcoded M13R primers were designed to generate PacBio sequencing ready amplicons from 16S rRNA target sequence flanked by M13 universal overhangs. All primers were synthesized and HPLC-purified (according to PacBio’s SMRT sequencing recommendation) by Integrated DNA Technology (San Jose, CA, USA).

To obtain barcoded 16S rRNA amplicons, we carried out the amplifications in two steps. First rounds of PCR were performed using M13-tagged 16S specific forward and reverse primers in a final volume of 20 μL consisting of 0.4 U of Phusion High-Fidelity DNA Polymerase (Thermo Fisher Scientific, Waltham, MA, USA), 1 × Phusion HF Buffer, 0.2 mM dNTPs, 1.5 mM MgCl_2_, 0.1 μM of each primer and distilled water to make the remainder of the 20 μL volume. Conditions used for amplification in the thermocycler were as follows: preincubation at 98 °C for 2 min, followed by 10 cycles of denaturation at 98 °C for 30 s, annealing at 66 °C for 15 s, elongation at 72 °C for 45 s and 10 cycles of denaturation at 98 °C for 30 s, annealing at 68 °C for 15 s, elongation at 72 °C for 45 s and a final extension step at 72 °C for 5 min. A unique combination of forward and reverse barcode sequences was added to 16S rRNA amplicons from each sample through the second round of PCR using M13F and M13R primers tagged with 16-base PacBio barcodes at their 5′ ends. PacBio barcode sequences are available from https://github.com/PacificBiosciences/Bioinformatics-Training/blob/master/barcoding/pacbio_384_barcodes.fasta. Primary PCR products were diluted 1:100, and 1 μL of the diluted products were used as templates for the secondary amplification reactions, which were carried out in a final volume of 20 μL consisting of 0.4 U of Phusion High-Fidelity DNA Polymerase (Thermo Fisher Scientific, Waltham, MA, USA), 1 × Phusion HF Buffer, 0.2 mM dNTPs, 1.5 mM MgCl_2_, 0.1 μM of each primer and distilled water to make the remainder of the 20 μL volume. Conditions used for amplification in the thermocycler were as follows: preincubation at 98 °C for 2 min, followed by 3 cycles of denaturation at 98 °C for 30 s, annealing at 63 °C for 15 s, elongation at 72 °C for 45 s and 5 cycles of denaturation at 98 °C for 30 s, annealing at 66 °C for 15 s, elongation at 72 °C for 45 s and a final extension step at 72 °C for 5 min. Barcoded 16S rRNA amplicons obtained from the secondary PCR were purified using Agentcourt AMPure XP magnetic beads (Beckman Coulter, Indianapolis, IN, USA) and quantified using a Qubit 2.0 Fluorometer and a Qubit dsDNA BR Assay Kit (Thermo Fisher Scientific, Waltham, MA, USA). Purified amplicons were then pooled in equimolar concentrations, and 500 ng of DNA was used for library preparation. Two PacBio libraries were constructed; each contained a pool of barcoded amplicons from nine samples. The SMRTbell adapters were ligated onto barcoded PCR products, and the libraries were sequenced on a PacBio RSII system using the P6-C4 polymerase and chemistry with a 360-min movie time.

### Sequence data analysis

PacBio raw reads were processed using RS_ReadsOfInsert protocol in the SMRT Analysis software version 2.3 to obtain demultiplexed concensus sequences with a minimum of three full passes. Sequence data were processed using the software package QIIME version 1.9.1^74^. Sequences shorter than 1000 nt were removed prior to downstream analyses. *De novo* chimeric detection was performed using the abundance-based algorithm implemented in UCHIME^75^ using a reference dataset from RDP^71^. The remaining sequences were clustered into OTUs based on an “open-reference” OTU-picking method at 97% identity using UCLUST^75^. Taxonomy was assigned to the representative sequence of each OTU using the RDP Classifier^22^ retrained toward the Greengenes database (V13.8)^76^. Diversity analyses of our samples were performed using the QIIME pipeline. To avoid biases generated by unequal sampling depth, the OTU table was rarefied to an even depth of 19,074 sequences per sample (corresponding to the number of sequences present in the sample with the lowest number of sequences) in comparison of all samples. After sequencing data were rarefied, the following alpha diversity measures were calculated: number of OTUs, Chao1 estimate of species richness, Shannon diversity, and Simpson diversity. Differences in relative abundance between samples from the Gulf of Thailand and Andaman Sea were compared using Welch’s *t-*test implemented in the Statistical Analyses of Metagenomic Profiles (STAMP) software^77^. The similarity among different bacterial assemblages was determined using the Euclidean distance matrix, and the dendrogram was calculated using an UPGMA clustering algorithm implemented in the STAMP software.

### Indicator species analysis and identification of *P. lutea* core microbiome

To analyse the strength and significance of the relationship between species occurrence or abundance and groups of sites, we employed INDVAL (indicator value index) method^54^ as implemented in R to identify species that were significantly associated with corals from different sampling sites (*P* < 0.05). The INDVAL method has previously been used to identify prokaryotic indicators of each particular coral habitat^49, 78^. Candidate members of the *P. lutea* core microbiome were identified at different levels of sample coverage (defined as the minimal percentage of all samples in which an OTU must be present to be considered part of the core microbiome). The percentages of sample coverage analysed ranged from 50% to 100% based on previous studies^79, 80^, with a 100% sample coverage being the most stringent criterion highlighting highly persistent bacterial species represented in all samples. Bacterial type strain sequences were downloaded from RDP and clustered at 100% identity using the CD-HIT software^81^ to obtain a set of non-redundant reference sequences. To identify candidate members of the core microbiome, full-length 16S rRNA sequences were aligned to the non-redundant RDP reference sequences using BLAST with a sequence identity cutoff of >90%.

### Comparison of species detection between full-length and partial 16S rRNA amplicons

To compare the ability of full-length 16S rRNA, V3-V4 and V5-V6 sequences in species identification, we extracted the V3-V4 and V5-V6 hypervariable regions from full-length sequences using the following flanking sequences: 5′-ACTCCTACGGGAGGCAGCAG (338 F) and 5′-CTACCAGGGTATCTAATC (786 R) for the V3-V4 region and 5′-GGATTAGATACCCTGGTAGTCC (785 F) and 5′-CTCACGRCACGAGCTGACG (1081 R) for the V5-V6 region. The full-length 16S rRNA sequences along with their respective V3-V4 and V5-V6 regions were aligned to the non-redundant RDP reference (bacterial type strain) sequences using BLAST with an E-value cutoff of 10^−10^. Any query sequence that returned two or more best hits belonging to different species with identical E-value, bit-score and aligned region was considered ineffective in resolving taxonomic classification at the species level. A neighbor-joining phylogenetic tree of selected species was constructed using MEGA7^82^. The clustering of the sequences was tested by a bootstrap approach using 1,000 replications.

## Electronic supplementary material

Supplementary Information

Dataset 1

## References

[1] Moberg F, Folke C (1999). Ecological goods and services of coral reef ecosystems. Ecological Economics.

[2] Forest R, Victor S, Farooq A, Nancy K (2002). Diversity and distribution of coral-associated bacteria. Marine Ecology Progress Series.

[3] Rosenberg E, Koren O, Reshef L, Efrony R, Zilber-Rosenberg I (2007). The role of microorganisms in coral health, disease and evolution. Nat Rev Micro.

[4] Thompson JR, Rivera HE, Closek CJ, Medina M (2014). Microbes in the coral holobiont: partners through evolution, development, and ecological interactions. Frontiers in Cellular and Infection Microbiology.

[5] Bourne DG, Munn CB (2005). Diversity of bacteria associated with the coral *Pocillopora damicornis* from the Great Barrier Reef. Environ Microbiol.

[6] Wegley L, Edwards R, Rodriguez-Brito B, Liu H, Rohwer F (2007). Metagenomic analysis of the microbial community associated with the coral *Porites astreoides*. Environ Microbiol.

[7] Raina JB, Tapiolas D, Willis BL, Bourne DG (2009). Coral-associated bacteria and their role in the biogeochemical cycling of sulfur. Appl Environ Microbiol.

[8] Lesser MP (2007). Nitrogen fixation by symbiotic cyanobacteria provides a source of nitrogen for the scleractinian coral *Montastraea cavernosa*. Marine Ecology Progress Series.

[9] Lema KA, Willis BL, Bourne DG (2012). Corals form characteristic associations with symbiotic nitrogen-fixing bacteria. Appl Environ Microbiol.

[10] Rädecker, N., Pogoreutz, C., Voolstra, C. R., Wiedenmann, J. & Wild, C. Nitrogen cycling in corals: the key to understanding holobiont functioning? *Trends in Microbiology* **23**, 490–497, doi:10.1016/j.tim.2015.03.008. 10.1016/j.tim.2015.03.008 25868684

[11] Ritchie, K. B. & Smith, G. W. in *Coral Health and Disease* (eds Eugene Rosenberg & Yossi Loya) 259–264 (Springer Berlin Heidelberg, 2004).

[12] Krediet CJ, Ritchie KB, Paul VJ, Teplitski M (2013). Coral-associated micro-organisms and their roles in promoting coral health and thwarting diseases. Proc Biol Sci.

[13] Sharp KH, Distel D, Paul VJ (2012). Diversity and dynamics of bacterial communities in early life stages of the Caribbean coral *Porites astreoides*. The ISME journal.

[14] Hong M-J, Yu Y-T, Chen CA, Chiang P-W, Tang S-L (2009). Influence of species specificity and other factors on bacteria associated with the coral *Stylophora pistillata* in Taiwan. Applied and Environmental Microbiology.

[15] Ceh J, Van Keulen M, Bourne DG (2011). Coral-associated bacterial communities on Ningaloo Reef, Western Australia. FEMS Microbiology Ecology.

[16] Bayer T (2013). The microbiome of the Red Sea coral *Stylophora pistillata* is dominated by tissue-associated *Endozoicomonas* bacteria. Applied and Environmental Microbiology.

[17] Gloor GB (2010). Microbiome profiling by Illumina sequencing of combinatorial sequence-tagged PCR products. PLOS ONE.

[18] Salipante SJ (2014). Performance comparison of Illumina and Ion Torrent next-generation sequencing platforms for 16S rRNA-based bacterial community profiling. Appl Environ Microbiol.

[19] Lee OO (2012). Spatial and species variations in bacterial communities associated with corals from the Red Sea as revealed by pyrosequencing. Appl Environ Microbiol.

[20] Cardenas A, Rodriguez RL, Pizarro V, Cadavid LF, Arevalo-Ferro C (2012). Shifts in bacterial communities of two Caribbean reef-building coral species affected by white plague disease. The ISME journal.

[21] Liu Z, DeSantis TZ, Andersen GL, Knight R (2008). Accurate taxonomy assignments from 16S rRNA sequences produced by highly parallel pyrosequencers. Nucleic Acids Res.

[22] Wang Q, Garrity GM, Tiedje JM, Cole JR (2007). Naïve Bayesian classifier for rapid assignment of rRNA sequences into the new bacterial taxonomy. Applied and Environmental Microbiology.

[23] Koren S, Phillippy AM (2015). One chromosome, one contig: complete microbial genomes from long-read sequencing and assembly. Curr Opin Microbiol.

[24] Roberts RJ, Carneiro MO, Schatz MC (2013). The advantages of SMRT sequencing. Genome Biology.

[25] Mosher JJ (2014). Improved performance of the PacBio SMRT technology for 16S rDNA sequencing. J Microbiol Methods.

[26] Myer PR, Kim M, Freetly HC, Smith TP (2016). Metagenomic and near full-length 16S rRNA sequence data in support of the phylogenetic analysis of the rumen bacterial community in steers. Data Brief.

[27] Wilkinson, C. *Status of coral reefs of the world: 2008*. 296 (Global Coral Reef Monitoring Network and Reef and Rainforest Research Center, 2008).

[28] Yeemin T (2009). Conditions of coral communities in the Gulf of Thailand: a decade after the 1998 severe bleaching event. Journal of Coral Reef Studies.

[29] Jasmin C, Anas A, Nair S (2015). Bacterial diversity associated with *Cinachyra cavernosa* and *Haliclona pigmentifera*, cohabiting sponges in the coral reef ecosystem of Gulf of Mannar, Southeast Coast of India. PLOS ONE.

[30] Ferrarini M (2013). An evaluation of the PacBio RS platform for sequencing and de novo assembly of a chloroplast genome. BMC Genomics.

[31] Frank JA (2016). Improved metagenome assemblies and taxonomic binning using long-read circular consensus sequence data. Scientific reports.

[32] Kozich JJ, Westcott SL, Baxter NT, Highlander SK, Schloss PD (2013). Development of a dual-index sequencing strategy and curation pipeline for analyzing amplicon sequence data on the MiSeq Illumina sequencing platform. Applied and Environmental Microbiology.

[33] Berry D, Ben Mahfoudh K, Wagner M, Loy A (2011). Barcoded primers used in multiplex amplicon pyrosequencing bias amplification. Appl Environ Microbiol.

[34] Li J (2013). Highly heterogeneous bacterial communities associated with the South China Sea Reef corals *Porites lutea*, *Galaxea fascicularis* and *Acropora millepora*. PLOS ONE.

[35] McKew BA (2012). Characterization of geographically distinct bacterial communities associated with coral mucus produced by *Acropora* spp. and *Porites* spp. Appl Environ Microbiol.

[36] Sere M (2016). Characterisation of an atypical manifestation of black band disease on *Porites lutea* in the Western Indian Ocean. PeerJ.

[37] Vega Thurber R (2009). Metagenomic analysis of stressed coral holobionts. Environ Microbiol.

[38] Neulinger SC, Jarnegren J, Ludvigsen M, Lochte K, Dullo WC (2008). Phenotype-specific bacterial communities in the cold-water coral *Lophelia pertusa* (Scleractinia) and their implications for the coral′s nutrition, health, and distribution. Appl Environ Microbiol.

[39] Kellogg CA, Ross SW, Brooke SD (2016). Bacterial community diversity of the deep-sea octocoral *Paramuricea placomus*. PeerJ.

[40] Meyer JL, Paul VJ, Teplitski M (2014). Community shifts in the surface microbiomes of the coral *Porites astreoides* with unusual lesions. PLOS ONE.

[41] Rodriguez-Lanetty M, Grandos-Cifuentes C, Barberan A, Bellantuono AJ, Bastidas C (2013). Ecological inferences from a deep screening of the complex bacterial consortia associated with the coral. Porites astreoides. Mol Ecol.

[42] Morrow KM, Moss AG, Chadwick NE, Liles MR (2012). Bacterial associates of two Caribbean coral species reveal species-specific distribution and geographic variability. Appl Environ Microbiol.

[43] Neave MJ (2017). Differential specificity between closely related corals and abundant *Endozoicomonas* endosymbionts across global scales. The ISME journal.

[44] Neave, M. J., Michell, C. T., Apprill, A. & Voolstra, C. R. Whole-genome sequences of three symbiotic *Endozoicomonas* strains. *Genome Announc* **2**, doi:10.1128/genomeA.00802-14 (2014). 10.1128/genomeA.00802-14 PMC4132622 25125646

[45] Neave MJ, Michell CT, Apprill A, Voolstra CR (2017). *Endozoicomonas* genomes reveal functional adaptation and plasticity in bacterial strains symbiotically associated with diverse marine hosts. Scientific reports.

[46] Roder C, Bayer T, Aranda M, Kruse M, Voolstra CR (2015). Microbiome structure of the fungid coral *Ctenactis echinata* aligns with environmental differences. Mol Ecol.

[47] Hernandez-Zulueta, J. *et al*. First deep screening of bacterial assemblages associated with corals of the Tropical Eastern Pacific. *FEMS Microbiol Ecol* **92**, doi:10.1093/femsec/fiw196 (2016). 10.1093/femsec/fiw196 27633927

[48] Hernandez-Agreda, A., Leggat, W., Bongaerts, P. & Ainsworth, T. D. The Microbial Signature Provides Insight into the Mechanistic Basis of Coral Success across Reef Habitats. *MBio* **7**, doi:10.1128/mBio.00560-16 (2016). 10.1128/mBio.00560-16 PMC4981706 27460792

[49] Li J (2014). Bacterial dynamics within the mucus, tissue and skeleton of the coral *Porites lutea* during different seasons. Scientific reports.

[50] Sere MG (2013). Bacterial communities associated with *Porites* white patch syndrome (PWPS) on three western Indian Ocean (WIO) coral reefs. PLoS One.

[51] Thijs, S. *et al*. Comparative Evaluation of Four Bacteria-Specific Primer Pairs for 16S rRNA Gene Surveys. *Frontiers in Microbiology* **8**, doi:10.3389/fmicb.2017.00494 (2017). 10.3389/fmicb.2017.00494 PMC5368227 28400755

[52] Shakya M (2013). Comparative metagenomic and rRNA microbial diversity characterization using archaeal and bacterial synthetic communities. Environ Microbiol.

[53] Klindworth A (2013). Evaluation of general 16S ribosomal RNA gene PCR primers for classical and next-generation sequencing-based diversity studies. Nucleic Acids Res.

[54] De Caceres M, Legendre P (2009). Associations between species and groups of sites: indices and statistical inference. Ecology.

[55] Munn, C. B. The role of *Vibrios* in diseases of corals. *Microbiol Spectr* **3**, doi:10.1128/microbiolspec.VE-0006-2014 (2015). 10.1128/microbiolspec.VE-0006-2014 26350314

[56] Bruto, M. *et al*. *Vibrio crassostreae*, a benign oyster colonizer turned into a pathogen after plasmid acquisition. *The ISME journal*, doi:10.1038/ismej.2016.162 (2016). 10.1038/ismej.2016.162 PMC5364345 27922600

[57] Rohwer F, Breitbart M, Jara J, Azam F, Knowlton N (2001). Diversity of bacteria associated with the Caribbean coral *Montastraea franksi*. Coral Reefs.

[58] Chu ND, Vollmer SV (2016). Caribbean corals house shared and host-specific microbial symbionts over time and space. Environ Microbiol Rep.

[59] Bayer T (2013). Bacteria of the genus *Endozoicomonas* dominate the microbiome of the Mediterranean gorgonian coral *Eunicella cavolini*. Marine Ecology Progress Series.

[60] Ainsworth TD (2015). The coral core microbiome identifies rare bacterial taxa as ubiquitous endosymbionts. The ISME journal.

[61] Jessen C (2013). *In-situ* effects of eutrophication and overfishing on physiology and bacterial diversity of the Red Sea coral *Acropora hemprichii*. PLOS ONE.

[62] Thogersen MS (2016). Production of the bioactive compounds violacein and indolmycin is conditional in a *maeA* mutant of *Pseudoalteromonas luteoviolacea* S4054 lacking the malic enzyme. Front Microbiol.

[63] Huang YL, Dobretsov S, Xiong H, Qian PY (2007). Effect of biofilm formation by *Pseudoalteromonas spongiae* on induction of larval settlement of the polychaete *Hydroides elegans*. Appl Environ Microbiol.

[64] Roder C (2014). Bacterial profiling of white plague disease in a comparative coral species framework. The ISME journal.

[65] Sweet M, Bythell J (2012). Ciliate and bacterial communities associated with White Syndrome and Brown Band Disease in reef-building corals. Environ Microbiol.

[66] Chimetto LA (2008). *Vibrios* dominate as culturable nitrogen-fixing bacteria of the Brazilian coral *Mussismilia hispida*. Systematic and Applied Microbiology.

[67] Cervino JM (2004). Relationship of *Vibrio* species infection and elevated temperatures to yellow blotch/band disease in Caribbean corals. Appl Environ Microbiol.

[68] Wang R (2015). The pathogenesis, detection, and prevention of Vibrio parahaemolyticus. Front Microbiol.

[69] Hernandez-Agreda A, Gates RD, Ainsworth TD (2017). Defining the Core Microbiome in Corals’ Microbial Soup. Trends Microbiol.

[70] Littman RA, Willis BL, Pfeffer C, Bourne DG (2009). Diversities of coral-associated bacteria differ with location, but not species, for three acroporid corals on the Great Barrier Reef. FEMS Microbiol Ecol.

[71] Cole JR (2014). Ribosomal Database Project: data and tools for high throughput rRNA analysis. Nucleic Acids Res.

[72] Singer E (2016). High-resolution phylogenetic microbial community profiling. The ISME journal.

[73] Youssef N (2009). Comparison of species richness estimates obtained using nearly complete fragments and simulated pyrosequencing-generated fragments in 16S rRNA gene-based environmental surveys. Appl Environ Microbiol.

[74] Caporaso JG (2010). QIIME allows analysis of high-throughput community sequencing data. Nat Methods.

[75] Edgar RC (2010). Search and clustering orders of magnitude faster than BLAST. Bioinformatics.

[76] DeSantis TZ (2006). Greengenes, a chimera-checked 16S rRNA gene database and workbench compatible with ARB. Applied and Environmental Microbiology.

[77] Parks DH, Tyson GW, Hugenholtz P, Beiko RG (2014). STAMP: statistical analysis of taxonomic and functional profiles. Bioinformatics.

[78] Glasl B, Herndl GJ, Frade PR (2016). The microbiome of coral surface mucus has a key role in mediating holobiont health and survival upon disturbance. The ISME journal.

[79] van de Water JAJM (2016). Spirochaetes dominate the microbial community associated with the red coral Corallium rubrum on a broad geographic scale. Scientific reports.

[80] Lawler, S. N. *et al*. Coral-Associated Bacterial Diversity Is Conserved across Two Deep-Sea Anthothela Species. *Frontiers in Microbiology* **7**, doi:10.3389/fmicb.2016.00458 (2016). 10.3389/fmicb.2016.00458 PMC4820459 27092120

[81] Fu L, Niu B, Zhu Z, Wu S, Li W (2012). CD-HIT: accelerated for clustering the next-generation sequencing data. Bioinformatics.

[82] Kumar S, Stecher G, Tamura K (2016). MEGA7: Molecular Evolutionary Genetics Analysis Version 7.0 for Bigger Datasets. Mol Biol Evol.

[83] Team, R. C. In *In R Foundation for Statistical Computing*. (ed. R. F. f S. Computing) (2016).

[84] Wickham, H. *ggplot2: Elegant graphis for data analysis*. (Springer-Verlag New York, 2009).

[85] Kahle D, Wickham H (2013). ggmap: Spatial visualization with ggplot2. The R Journal.

