# Supplementary material for: High resolution profiling of coral-associated bacterial communities using full-length 16S rRNA sequence data from PacBio SMRT sequencing system
Source: Sci Rep. 2017 Jun 5;7:2774. doi: 10.1038/s41598-017-03139-4 (PMC5459821; doi:10.1038/s41598-017-03139-4)
Supplement: Supplementary file 1 — Supplementary Information [file 41598_2017_3139_MOESM1_ESM.pdf]

Supplementary Information

High resolution profiling of coral-associated bacterial communities using full-length 16S rRNA sequence data from PacBio SMRT sequencing system

Wirulda Pootakham[1,*], Wuttichai Mhuantong[1], Thippawan Yoocha[1], Lalita Putchim[2], Chutima Sonthirod[1], Chaiwat Naktang[1], Nalinee Thongtham[2] and Sithichoke Tangphatsornruang[1].

[1]National Center for Genetic Engineering and Biotechnology (BIOTEC), National Science and Technology Development Agency, Pathum Thani, Thailand
[2]Phuket Marine Biological Center, Phuket, 83000, Thailand

[*]Corresponding author
Wirulda Pootakham
National Center for Genetic Engineering and Biotechnology (BIOTEC), 113 Thailand Science Park, Phahonyothin Rd, Khlong Nueng, Khlong Luang, Pathum Thani, Thailand 12120
Email address: wirulda@alumni.stanford.edu
Phone: 66 2 564 6700 ext 3548

Supplementary Table S1. A list of indicator species significantly (*P* < 0.05) associated with corals from particular location (MN, Mannai; TA, Tao; TL, Talu; MT, Maiton; TC, Tachai) as revealed by the indicator value (INDVAL) analysis. The analysis showed that none of the bacterial species was significantly associated with coral samples from SM.

Location		Indicator species	Taxonomy	Index value	*P*-value
Gulf of Thailand	MN	*Aliiroseovarius crassostreae*	Bacteria;Proteobacteria;Alphaproteobacteria;Rhodobacterales;Rhodobacteraceae;Aliiroseovarius	1.00	0.008
		Arcobacter defluvii	Bacteria;Proteobacteria;Epsilonproteobacteria;Campylobacterales;Campylobacteraceae;Arcobacter	1.00	0.009
		Arcobacter halophilus	Bacteria;Proteobacteria;Epsilonproteobacteria;Campylobacterales;Campylobacteraceae;Arcobacter	1.00	0.006
		Halobacteriovorax litoralis	Bacteria;Proteobacteria;Deltaproteobacteria;Bdellovibrionales;Halobacteriovoraceae;Halobacteriovorax	1.00	0.009
		Leisingera aquimarina	Bacteria;Proteobacteria;Alphaproteobacteria;Rhodobacterales;Rhodobacteraceae;Leisingera	1.00	0.006
		Litoribrevibacter albus	Bacteria;Proteobacteria;Gammaproteobacteria;Oceanospirillales;Oceanospirillaceae;Litoribrevibacter	1.00	0.007
		Marinifilum fragile	Bacteria;Bacteroidetes;Bacteroidia;Bacteroidales;Marinifilaceae;Marinifilum	1.00	0.003
		Photobacterium angustum	Bacteria;Proteobacteria;Gammaproteobacteria;Vibrionales;Vibrionaceae;Photobacterium	1.00	0.007
		Roseovarius aestuarii	Bacteria;Proteobacteria;Alphaproteobacteria;Rhodobacterales;Rhodobacteraceae;Roseovarius	1.00	0.006
		Tenacibaculum gallaicum	Bacteria;Bacteroidetes;Flavobacteriia;Flavobacteriales;Flavobacteriaceae;Tenacibaculum	1.00	0.006
		Thalassobius aestuarii	Bacteria;Proteobacteria;Alphaproteobacteria;Rhodobacterales;Rhodobacteraceae;Thalassobius	1.00	0.007
		Tropicibacter mediterraneus	Bacteria;Proteobacteria;Alphaproteobacteria;Rhodobacterales;Rhodobacteraceae;Tropicibacter	1.00	0.013
		Tropicibacter multivorans	Bacteria;Proteobacteria;Alphaproteobacteria;Rhodobacterales;Rhodobacteraceae;Tropicibacter	1.00	0.017
		Vallitalea guaymasensis	Bacteria;Firmicutes;Clostridia;Clostridiales;Defluviitaleaceae;Vallitalea	1.00	0.003
		Fusibacter paucivorans	Bacteria;Firmicutes;Clostridia;Clostridiales;Clostridialesincertaesedis;Clostridiales;Fusibacter	1.00	0.009
		Shimia isoporae	Bacteria;Proteobacteria;Alphaproteobacteria;Rhodobacterales;Rhodobacteraceae;Shimia	1.00	0.013
		Thalassobius mediterraneus	Bacteria;Proteobacteria;Alphaproteobacteria;Rhodobacterales;Rhodobacteraceae;Thalassobius	1.00	0.011
		Arcobacter butzleri	Bacteria;Proteobacteria;Epsilonproteobacteria;Campylobacterales;Campylobacteraceae;Arcobacter	1.00	0.009
		Vallitalea pronyensis	Bacteria;Firmicutes;Clostridia;Clostridiales;Defluviitaleaceae;Vallitalea	1.00	0.007
		Arcobacter bivalviorum	Bacteria;Proteobacteria;Epsilonproteobacteria;Campylobacterales;Campylobacteraceae;Arcobacter	0.99	0.008
		Arcobacter nitrofigilis	Bacteria;Proteobacteria;Epsilonproteobacteria;Campylobacterales;Campylobacteraceae;Arcobacter	0.99	0.006
		Oceanirhabdus sediminicola	Bacteria;Firmicutes;Clostridia;Clostridiales;Clostridiaceae;Oceanirhabdus	0.99	0.008
		Aestuariispira insulae	Bacteria;Proteobacteria;Alphaproteobacteria;Rhodospirillales;Rhodospirillaceae;Aestuariispira	0.98	0.008
		Shimia marina	Bacteria;Proteobacteria;Alphaproteobacteria;Rhodobacterales;Rhodobacteraceae;Shimia	0.98	0.007
		Marinifilum flexuosum	Bacteria;Bacteroidetes;Bacteroidia;Bacteroidales;Marinifilaceae;Marinifilum	0.98	0.011
		Thalassolituus marinus	Bacteria;Proteobacteria;Gammaproteobacteria;Oceanospirillales;Oceanospirillaceae;Thalassolituus	0.98	0.007

		Tenacibaculum litopenaei	Bacteria;Bacteroidetes;Flavobacteriia;Flavobacteriales;Flavobacteriaceae;Tenacibaculum	0.97	0.012
		Vibrio gallaecicus	Bacteria;Proteobacteria;Gammaproteobacteria;Vibrionales;Vibrionaceae;Vibrio	0.95	0.019
		Marinobacterium stanieri	Bacteria;Proteobacteria;Gammaproteobacteria;Oceanospirillales;Oceanospirillaceae;Marinobacterium	0.95	0.004
		Neptunomonas antarctica	Bacteria;Proteobacteria;Gammaproteobacteria;Oceanospirillales;Oceanospirillaceae;Neptunomonas	0.93	0.016
		Thalassobius maritimus	Bacteria;Proteobacteria;Alphaproteobacteria;Rhodobacterales;Rhodobacteraceae;Thalassobius	0.93	0.009
	TA	*Vibrio crassostreae*	Bacteria;Proteobacteria;Gammaproteobacteria;Vibrionales;Vibrionaceae;Vibrio	1.00	0.006
		Pseudoalteromonas phenolica	Bacteria;Proteobacteria;Gammaproteobacteria;Alteromonadales;Pseudoalteromonadaceae;Pseudoalteromonas	0.96	0.009
		Vibrio coralliilyticus	Bacteria;Proteobacteria;Gammaproteobacteria;Vibrionales;Vibrionaceae;Vibrio	0.93	0.009
		Pseudoalteromonas luteoviolacea	Bacteria;Proteobacteria;Gammaproteobacteria;Alteromonadales;Pseudoalteromonadaceae;Pseudoalteromonas	0.92	0.006
		Psychrosphaera saromensis	Proteobacteria;Gammaproteobacteria;Alteromonadales;Pseudoalteromonadaceae;Psychrosphaera	0.88	0.017
		Vibrio caribbeanicus	Bacteria;Proteobacteria;Gammaproteobacteria;Vibrionales;Vibrionaceae;Vibrio	0.88	0.013
		Vibrio hepatarius	Bacteria;Proteobacteria;Gammaproteobacteria;Vibrionales;Vibrionaceae;Vibrio;Vibriooreintalisgroup	0.87	0.048
		Thalassotalea ponticola	Bacteria;Proteobacteria;Gammaproteobacteria;Alteromonadales;Colwelliaceae;Thalassotalea	0.87	0.008
		Psychrosphaera aestuarii	Bacteria;Proteobacteria;Gammaproteobacteria;Alteromonadales;Pseudoalteromonadaceae;Psychrosphaera	0.83	0.015
		Pseudoalteromonas piscicida	Bacteria;Proteobacteria;Gammaproteobacteria;Alteromonadales;Pseudoalteromonadaceae;Pseudoalteromonas	0.82	0.012
		Shewanella corallii	Bacteria;Proteobacteria;Gammaproteobacteria;Alteromonadales;Shewanellaceae;Shewanella	0.81	0.016
	TL	*Alteromonas marina*	Bacteria;Proteobacteria;Gammaproteobacteria;Alteromonadales;Alteromonadaceae;Alteromonas	0.93	0.01
		Pseudoalteromonas shioyasakiensis	Bacteria;Proteobacteria;Gammaproteobacteria;Alteromonadales;Pseudoalteromonadaceae;Pseudoalteromonas	0.84	0.013
		Pseudoalteromonas mariniglutinosa	Bacteria;Proteobacteria;Gammaproteobacteria;Alteromonadales;Pseudoalteromonadaceae;Pseudoalteromonas	0.84	0.015
		Pseudoalteromonas lipolytica	Bacteria;Proteobacteria;Gammaproteobacteria;Alteromonadales;Pseudoalteromonadaceae;Pseudoalteromonas	0.81	0.014
		Pseudoalteromonas antarctica	Bacteria;Proteobacteria;Gammaproteobacteria;Alteromonadales;Pseudoalteromonadaceae;Pseudoalteromonas	0.80	0.008
Andaman Sea	MT	*Agrococcus jenensis*	Bacteria;Actinobacteria;Actinobacteria;Micrococcales;Microbacteriaceae;Agrococcus	1.00	0.009
		Brevundimonas nasdae	Bacteria;Proteobacteria;Alphaproteobacteria;Caulobacterales;Caulobacteraceae;Brevundimonas	1.00	0.003
		Methylobacterium populi	Bacteria;Proteobacteria;Alphaproteobacteria;Rhizobiales;Methylobacteriaceae;Methylobacterium	1.00	0.012
		Acinetobacter schindleri	Bacteria;Proteobacteria;Gammaproteobacteria;Pseudomonadales;Moraxellaceae;Acinetobacter	0.99	0.017
		Bradyrhizobium japonicum	Bacteria;Proteobacteria;Alphaproteobacteria;Rhizobiales;Bradyrhizobiaceae;Bradyrhizobium	0.90	0.011
		Bradyrhizobium diazoefficiens	Bacteria;Proteobacteria;Alphaproteobacteria;Rhizobiales;Bradyrhizobiaceae;Bradyrhizobium	0.84	0.009
	TC	*Burkholderia stabilis*	Bacteria;Proteobacteria;Betaproteobacteria;Burkholderiales;Burkholderiaceae;Burkholderia	1.00	0.008
		Heliimonas saccharivorans	Bacteria;Bacteroidetes;Chitinophagia;Chitinophagales;Chitinophagaceae;Heliimonas	1.00	0.012
		Devosia insulae	Bacteria;Proteobacteria;Alphaproteobacteria;Rhizobiales;Hyphomicrobiaceae;Devosia	0.98	0.006

Supplementary Table S2. A list of candidate members of the P. lutea core microbiome (at 50% sample coverage), their relative abundance (average species abundance across all P. lutea samples) and their average ubiquity (percentages of P. lutea samples in which the species was detected).

Species	Taxonomy	Relative abundance	Average ubiquity
Endozoicomonas elysicola	Bacteria;Proteobacteria;Gammaproteobacteria;Oceanospirillales;Hahellaceae;Endozoicomonas	21.340%	100%
Endozoicomonas euniceicola	Bacteria;Proteobacteria;Gammaproteobacteria;Oceanospirillales;Hahellaceae;Endozoicomonas	17.399%	100%
Pseudoalteromonas shioyasakiensis	Bacteria;Proteobacteria;Gammaproteobacteria;Alteromonadales;Pseudoalteromonadaceae;Pseudoalteromonas	6.637%	94%
Sediminibacterium salmoneum	Bacteria;Bacteroidetes;Chitinophagia;Chitinophagales;Chitinophagaceae;Sediminibacterium	4.336%	100%
Reyranella massiliensis	Bacteria;Proteobacteria;Alphaproteobacteria;Rhodospirillales;unclassified Rhodospirillales;Reyranella	3.881%	100%
Endozoicomonas numazuensis	Bacteria;Proteobacteria;Gammaproteobacteria;Oceanospirillales;Hahellaceae;Endozoicomonas	3.637%	100%
Arcobacter nitrofigilis	Bacteria;Proteobacteria;Epsilonproteobacteria;Campylobacterales;Campylobacteraceae;Arcobacter	2.665%	83%
Caulobacter vibrioides	Bacteria;Proteobacteria;Alphaproteobacteria;Caulobacterales;Caulobacteraceae;Caulobacter	2.657%	100%
Pseudoalteromonas phenolica	Bacteria;Proteobacteria;Gammaproteobacteria;Alteromonadales;Pseudoalteromonadaceae;Pseudoalteromonas	2.371%	67%
Kistimonas asteriae	Bacteria;Proteobacteria;Gammaproteobacteria;Oceanospirillales;Hahellaceae;Kistimonas	2.292%	94%
Psychrosphaera saromensis	Bacteria;Proteobacteria;Gammaproteobacteria;Alteromonadales;Pseudoalteromonadaceae;Psychrosphaera	2.255%	83%
Endozoicomonas montiporae	Bacteria;Proteobacteria;Gammaproteobacteria;Oceanospirillales;Hahellaceae;Endozoicomonas	2.125%	94%
Vibrio fortis	Bacteria;Proteobacteria;Gammaproteobacteria;Vibrionales;Vibrionaceae;Vibrio	1.882%	94%
Arcobacter bivalviorum	Bacteria;Proteobacteria;Epsilonproteobacteria;Campylobacterales;Campylobacteraceae;Arcobacter	1.767%	72%
Prosthecomicrobium hirschii	Bacteria;Proteobacteria;Alphaproteobacteria;Rhizobiales;Hyphomicrobiaceae;Prosthecomicrobium	1.652%	100%
Vibrio parahaemolyticus	Bacteria;Proteobacteria;Gammaproteobacteria;Vibrionales;Vibrionaceae;Vibrio;Vibrio harveyi group	1.597%	89%
Bradyrhizobium rifense	Bacteria;Proteobacteria;Alphaproteobacteria;Rhizobiales;Bradyrhizobiaceae;Bradyrhizobium	1.570%	100%
Pseudoalteromonas luteoviolacea	Bacteria;Proteobacteria;Gammaproteobacteria;Alteromonadales;Pseudoalteromonadaceae;Pseudoalteromonas	1.412%	78%
Vibrio hepatarius	Bacteria;Proteobacteria;Gammaproteobacteria;Vibrionales;Vibrionaceae;Vibrio;Vibrio oreintalis group	1.251%	78%
Pseudoalteromonas spongiae	Bacteria;Proteobacteria;Gammaproteobacteria;Alteromonadales;Pseudoalteromonadaceae;Pseudoalteromonas	0.946%	78%
Vibrio maritimus	Bacteria;Proteobacteria;Gammaproteobacteria;Vibrionales;Vibrionaceae;Vibrio	0.586%	89%
Parasegetibacter terrae	Bacteria;Bacteroidetes;Chitinophagia;Chitinophagales;Chitinophagaceae;Parasegetibacter	0.586%	94%
Vibrio brasiliensis	Bacteria;Proteobacteria;Gammaproteobacteria;Vibrionales;Vibrionaceae;Vibrio;Vibrio oreintalis group	0.553%	83%
Nevskia terrae	Bacteria;Proteobacteria;Gammaproteobacteria;Nevskiales;Sinobacteraceae;Nevskia	0.507%	100%

Vibrio rotiferianus	Bacteria;Proteobacteria;Gammaproteobacteria;Vibrionales;Vibrionaceae;Vibrio;Vibrio harveyi group	0.443%	78%
Rhizomicrobium palustre	Bacteria;Proteobacteria;Alphaproteobacteria;Micropepsales;Micropepsaceae;Rhizomicrobium	0.434%	94%
Burkholderia fungorum	Bacteria;Proteobacteria;Betaproteobacteria;Burkholderiales;Burkholderiaceae;Paraburkholderia	0.382%	89%
Alteromonas macleodii	Bacteria;Proteobacteria;Gammaproteobacteria;Alteromonadales;Alteromonadaceae;Alteromonas	0.348%	67%
Sediminibacterium goheungense	Bacteria;Bacteroidetes;Chitinophagia;Chitinophagales;Chitinophagaceae;Sediminibacterium	0.342%	94%
Steroidobacter agariperforans	Bacteria;Proteobacteria;Gammaproteobacteria;Nevskiales;Sinobacteraceae;Steroidobacter	0.334%	89%
Vibrio coralliilyticus	Bacteria;Proteobacteria;Gammaproteobacteria;Vibrionales;Vibrionaceae;Vibrio	0.319%	67%
Hyphomicrobium facile	Bacteria;Proteobacteria;Alphaproteobacteria;Rhizobiales;Hyphomicrobiaceae;Hyphomicrobium	0.293%	89%
Hydrocarboniphaga effusa	Bacteria;Proteobacteria;Gammaproteobacteria;Nevskiales;Sinobacteraceae;Hydrocarboniphaga	0.292%	94%
Endozoicomonas gorgoniicola	Bacteria;Proteobacteria;Gammaproteobacteria;Oceanospirillales;Hahellaceae;Endozoicomonas	0.259%	67%
Vibrio furnissii	Bacteria;Proteobacteria;Gammaproteobacteria;Vibrionales;Vibrionaceae;Vibrio	0.242%	61%
Hydrocarboniphaga daqingensis	Bacteria;Proteobacteria;Gammaproteobacteria;Nevskiales;Sinobacteraceae;Hydrocarboniphaga	0.239%	89%
Bradyrhizobium oligotrophicum	Bacteria;Proteobacteria;Alphaproteobacteria;Rhizobiales;Bradyrhizobiaceae;Bradyrhizobium	0.224%	83%
Fimbriimonas ginsengisoli	Bacteria;Armatimonadetes;Fimbriimonadia;Fimbriimonadales;Fimbriimonadaceae;Fimbriimonas	0.204%	72%
Asinibacterium lactis	Bacteria;Bacteroidetes;Chitinophagia;Chitinophagales;Chitinophagaceae;Asinibacterium	0.195%	78%
Caulobacter henricii	Bacteria;Proteobacteria;Alphaproteobacteria;Caulobacterales;Caulobacteraceae;Caulobacter	0.192%	89%
Pseudoalteromonas lipolytica	Bacteria;Proteobacteria;Gammaproteobacteria;Alteromonadales;Pseudoalteromonadaceae;Pseudoalteromonas	0.190%	67%
Thalassotalea loyana	Bacteria;Proteobacteria;Gammaproteobacteria;Alteromonadales;Colwelliaceae;Thalassotalea	0.145%	67%
Photobacterium rosenbergii	Bacteria;Proteobacteria;Gammaproteobacteria;Vibrionales;Vibrionaceae;Photobacterium	0.145%	56%
Vibrio agarivorans	Bacteria;Proteobacteria;Gammaproteobacteria;Vibrionales;Vibrionaceae;Vibrio	0.119%	61%
Methylocystis heyeri	Bacteria;Proteobacteria;Alphaproteobacteria;Rhizobiales;Methylocystaceae;Methylocystis	0.115%	83%
Amphritea balenae	Bacteria;Proteobacteria;Gammaproteobacteria;Oceanospirillales;Oceanospirillaceae;Amphritea	0.108%	61%
Oligotropha carboxidovorans	Bacteria;Proteobacteria;Alphaproteobacteria;Rhizobiales;Bradyrhizobiaceae;Oligotropha	0.099%	61%
Vibrio caribbeanicus	Bacteria;Proteobacteria;Gammaproteobacteria;Vibrionales;Vibrionaceae;Vibrio	0.089%	56%
Endobacter medicaginis	Bacteria;Proteobacteria;Alphaproteobacteria;Rhodospirillales;Acetobacteraceae;Endobacter	0.084%	78%
Vibrio ponticus	Bacteria;Proteobacteria;Gammaproteobacteria;Vibrionales;Vibrionaceae;Vibrio	0.081%	50%
Vibrio neptunius	Bacteria;Proteobacteria;Gammaproteobacteria;Vibrionales;Vibrionaceae;Vibrio	0.080%	67%
Rhizomicrobium electricum	Bacteria;Proteobacteria;Alphaproteobacteria;Micropepsales;Micropepsaceae;Rhizomicrobium	0.080%	89%
Endozoicomonas atrinae	Bacteria;Proteobacteria;Gammaproteobacteria;Oceanospirillales;Hahellaceae;Endozoicomonas	0.073%	72%
Alteromonas mediterranea	Bacteria;Proteobacteria;Gammaproteobacteria;Alteromonadales;Alteromonadaceae;Alteromonas	0.069%	61%

Vibrio harveyi	Bacteria;Proteobacteria;Gammaproteobacteria;Vibrionales;Vibrionaceae;Vibrio;Vibrio harveyi group	0.066%	56%
Vibrio pelagius	Bacteria;Proteobacteria;Gammaproteobacteria;Vibrionales;Vibrionaceae;Vibrio	0.060%	56%
Salinirepens amamiensis	Bacteria;Bacteroidetes;Flavobacteriia;Flavobacteriales;Crocinitomicaceae;Salinirepens	0.050%	67%
Prochlorococcus marinus	Bacteria;Cyanobacteria;Synechococcales;Prochloraceae;Prochlorococcus	0.044%	61%
Tropicibacter phthalicicus	Bacteria;Proteobacteria;Alphaproteobacteria;Rhodobacterales;Rhodobacteraceae;Tropicibacter	0.042%	72%
Pseudoalteromonas rubra	Bacteria;Proteobacteria;Gammaproteobacteria;Alteromonadales;Pseudoalteromonadaceae;Pseudoalteromonas	0.042%	50%
Telmatospirillum siberiense	Bacteria;Proteobacteria;Alphaproteobacteria;Rhodospirillales;Rhodospirillaceae;Telmatospirillum	0.040%	61%
Reyranella soli	Bacteria;Proteobacteria;Alphaproteobacteria;Rhodospirillales;unclassified Rhodospirillales;Reyranella	0.038%	72%
Pseudoalteromonas flavipulchra	Bacteria;Proteobacteria;Gammaproteobacteria;Alteromonadales;Pseudoalteromonadaceae;Pseudoalteromonas	0.038%	61%
Thalassotalea ganghwensis	Bacteria;Proteobacteria;Gammaproteobacteria;Alteromonadales;Colwelliaceae;Thalassotalea	0.036%	50%
Pseudoalteromonas agarivorans	Bacteria;Proteobacteria;Gammaproteobacteria;Alteromonadales;Pseudoalteromonadaceae;Pseudoalteromonas	0.034%	61%
Pseudoalteromonas tetraodonis	Bacteria;Proteobacteria;Gammaproteobacteria;Alteromonadales;Pseudoalteromonadaceae;Pseudoalteromonas	0.033%	67%
Vibrio mediterranei	Bacteria;Proteobacteria;Gammaproteobacteria;Vibrionales;Vibrionaceae;Vibrio	0.032%	50%
Bradyrhizobium pachyrhizi	Bacteria;Proteobacteria;Alphaproteobacteria;Rhizobiales;Bradyrhizobiaceae;Bradyrhizobium	0.031%	50%
Pseudoalteromonas arabiensis	Bacteria;Proteobacteria;Gammaproteobacteria;Alteromonadales;Pseudoalteromonadaceae;Pseudoalteromonas	0.029%	50%
Povalibacter uvarum	Bacteria;Proteobacteria;Gammaproteobacteria;Nevskiales;Sinobacteraceae;Povalibacter	0.027%	50%
Aquibacter zeaxanthinifaciens	Bacteria;Bacteroidetes;Flavobacteriia;Flavobacteriales;Flavobacteriaceae;Aquibacter	0.026%	50%
Alteromonas mediterranea	Bacteria;Proteobacteria;Gammaproteobacteria;Alteromonadales;Alteromonadaceae;Alteromonas	0.026%	50%
Vibrio natriegens	Bacteria;Proteobacteria;Gammaproteobacteria;Vibrionales;Vibrionaceae;Vibrio;Vibrio harveyi group	0.026%	50%
Vibrio alginolyticus	Bacteria;Proteobacteria;Gammaproteobacteria;Vibrionales;Vibrionaceae;Vibrio;Vibrio harveyi group	0.021%	56%
Vibrio inhibens	Bacteria;Proteobacteria;Gammaproteobacteria;Vibrionales;Vibrionaceae;Vibrio	0.021%	50%
Pseudoalteromonas aurantia	Bacteria;Proteobacteria;Gammaproteobacteria;Alteromonadales;Pseudoalteromonadaceae;Pseudoalteromonas	0.020%	56%
Acidisphaera rubrifaciens	Bacteria;Proteobacteria;Alphaproteobacteria;Rhodospirillales;Acetobacteraceae;Acidisphaera	0.020%	56%
Pseudoalteromonas antarctica	Bacteria;Proteobacteria;Gammaproteobacteria;Alteromonadales;Pseudoalteromonadaceae;Pseudoalteromonas	0.019%	50%
Vibrio tubiashii	Bacteria;Proteobacteria;Gammaproteobacteria;Vibrionales;Vibrionaceae;Vibrio;Vibrio oreintalis group	0.019%	50%
Pseudoalteromonas aliena	Bacteria;Proteobacteria;Gammaproteobacteria;Alteromonadales;Pseudoalteromonadaceae;Pseudoalteromonas	0.017%	56%
Caulobacter mirabilis	Bacteria;Proteobacteria;Alphaproteobacteria;Caulobacterales;Caulobacteraceae;Caulobacter	0.017%	61%
Pseudoalteromonas translucida	Bacteria;Proteobacteria;Gammaproteobacteria;Alteromonadales;Pseudoalteromonadaceae;Pseudoalteromonas	0.015%	61%
Nisaea nitritireducens	Bacteria;Proteobacteria;Alphaproteobacteria;Rhodospirillales;Rhodospirillaceae;Nisaea	0.015%	61%
Ichthyenterobacterium magnum	Bacteria;Bacteroidetes;Flavobacteriia;Flavobacteriales;Flavobacteriaceae;Ichthyenterobacterium	0.014%	50%

Supplementary Table S3. Performance of full-length 16S, V3-V4 or V5-V6 regions in identifying P. lutea-associated bacteria at the species level.

Genus found in P. lutea samples	Number of species in the genus	Number (and %) of classifiable species using full-length sequences		Number (and %) of classifiable species using V3-V4 sequences		Number (and %) of classifiable species using V5-V6 sequences	
Acinetobacter	38	38	100.0%	37	97.4%	19	50.0%
Afipia	5	5	100.0%	5	100.0%	5	100.0%
Agarivorans	3	3	100.0%	3	100.0%	2	66.7%
Agrococcus	9	9	100.0%	6	66.7%	8	88.9%
Alcanivorax	9	9	100.0%	8	88.9%	8	88.9%
Alteromonas	13	13	100.0%	8	61.5%	12	92.3%
Amphritea	4	4	100.0%	4	100.0%	4	100.0%
Arcobacter	17	17	100.0%	16	94.1%	16	94.1%
Asticcacaulis	6	6	100.0%	6	100.0%	6	100.0%
Bacillus	198	197	99.5%	175	88.4%	150	75.8%
Bacteriovorax	1	1	100.0%	1	100.0%	1	100.0%
Balneola	2	2	100.0%	2	100.0%	2	100.0%
Bradyrhizobium	23	22	95.7%	6	26.1%	10	43.5%
Burkholderia	85	85	100.0%	82	96.5%	63	74.1%
Clostridium	164	164	100.0%	156	95.1%	145	88.4%
Cohaesibacter	3	3	100.0%	3	100.0%	3	100.0%
Congregibacter	1	1	100.0%	1	100.0%	1	100.0%
Coraliomargarita	2	2	100.0%	1	50.0%	1	50.0%
Desulfobacter	6	6	100.0%	6	100.0%	6	100.0%
Desulfococcus	2	2	100.0%	2	100.0%	2	100.0%
Devosia	16	16	100.0%	14	87.5%	16	100.0%
Enhydrobacter	1	1	100.0%	1	100.0%	1	100.0%
Enterovibrio	4	4	100.0%	4	100.0%	4	100.0%
Ferrimonas	7	7	100.0%	7	100.0%	7	100.0%

Fimbriimonas	1	1	100.0%	1	100.0%	1	100.0%
Flammeovirga	5	5	100.0%	5	100.0%	5	100.0%
Flavobacterium	121	121	100.0%	115	95.0%	97	80.2%
Fluviicola	2	2	100.0%	2	100.0%	2	100.0%
Francisella	7	7	100.0%	7	100.0%	5	71.4%
Fulvivirga	2	2	100.0%	2	100.0%	2	100.0%
Fusibacter	3	3	100.0%	3	100.0%	3	100.0%
Gemmata	1	1	100.0%	1	100.0%	1	100.0%
Glaciecola	3	3	100.0%	3	100.0%	3	100.0%
Hymenobacter	33	33	100.0%	33	100.0%	28	84.8%
Hyphomicrobium	9	9	100.0%	8	88.9%	9	100.0%
Idiomarina	23	23	100.0%	23	100.0%	19	82.6%
Janthinobacterium	2	2	100.0%	2	100.0%	2	100.0%
Lentisphaera	2	2	100.0%	1	50.0%	2	100.0%
Luteimonas	11	11	100.0%	11	100.0%	11	100.0%
Lysobacter	28	28	100.0%	28	100.0%	26	92.9%
Magnetospirillum	2	2	100.0%	2	100.0%	2	100.0%
Marinomonas	24	24	100.0%	24	100.0%	18	75.0%
Methylobacterium	48	48	100.0%	33	68.8%	45	93.8%
Methyloversatilis	2	2	100.0%	2	100.0%	2	100.0%
Nautella	1	1	100.0%	1	100.0%	1	100.0%
Neptunomonas	7	7	100.0%	7	100.0%	7	100.0%
Nevskia	5	5	100.0%	5	100.0%	5	100.0%
Oceanospirillum	4	4	100.0%	4	100.0%	4	100.0%
Oleibacter	1	1	100.0%	1	100.0%	1	100.0%
Oleispira	2	2	100.0%	2	100.0%	2	100.0%
Pedomicrobium	4	4	100.0%	4	100.0%	2	50.0%
Persicobacter	2	2	100.0%	2	100.0%	2	100.0%
Phaeobacter	2	2	100.0%	2	100.0%	2	100.0%
Photobacterium	24	24	100.0%	24	100.0%	21	87.5%

Prochlorococcus	1	1	100.0%	1	100.0%	1	100.0%
Propionibacterium	14	14	100.0%	9	64.3%	14	100.0%
Propionigenium	2	2	100.0%	2	100.0%	2	100.0%
Pseudoalteromonas	39	39	100.0%	27	69.2%	31	79.5%
Pseudomonas	157	156	99.4%	130	82.8%	85	54.1%
Pseudoruegeria	4	4	100.0%	4	100.0%	4	100.0%
Psychrobacter	34	34	100.0%	29	85.3%	10	29.4%
Reinekea	3	3	100.0%	3	100.0%	3	100.0%
Rhodoplanes	6	6	100.0%	5	83.3%	6	100.0%
Roseivirga	3	3	100.0%	3	100.0%	3	100.0%
Salinisphaera	6	6	100.0%	5	83.3%	5	83.3%
Saprospira	2	2	100.0%	2	100.0%	2	100.0%
Sediminibacterium	3	3	100.0%	3	100.0%	2	66.7%
Shewanella	61	60	98.4%	54	88.5%	42	68.9%
Sphingobium	34	34	100.0%	28	82.4%	31	91.2%
Sphingomonas	70	70	100.0%	57	81.4%	62	88.6%
Spirochaeta	16	16	100.0%	16	100.0%	15	93.8%
Spongiibacter	2	2	100.0%	2	100.0%	2	100.0%
Staphylococcus	47	47	100.0%	38	80.9%	26	55.3%
Stenotrophomonas	11	11	100.0%	11	100.0%	8	72.7%
Telmatospirillum	1	1	100.0%	1	100.0%	1	100.0%
Tenacibaculum	20	20	100.0%	19	95.0%	20	100.0%
Tepidibacter	3	3	100.0%	3	100.0%	3	100.0%
Thalassobius	4	4	100.0%	4	100.0%	4	100.0%
Thalassococcus	2	2	100.0%	2	100.0%	2	100.0%
Thalassomonas	4	4	100.0%	4	100.0%	4	100.0%
Thalassospira	8	8	100.0%	7	87.5%	5	62.5%
Vibrio	100	100	100.0%	93	93.0%	55	55.0%

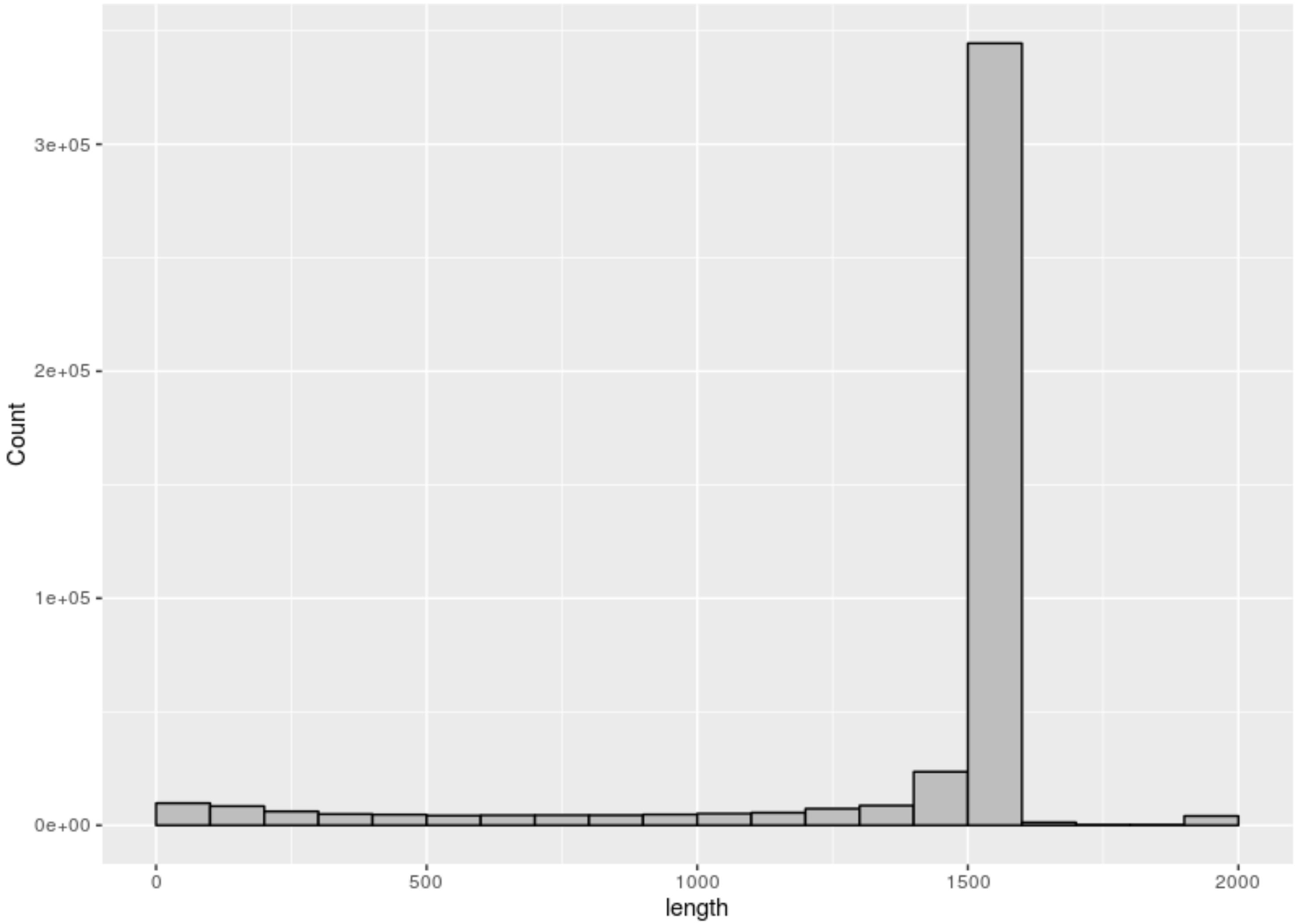

Supplementary Figure S1: Length distribution of PacBio CCS reads.

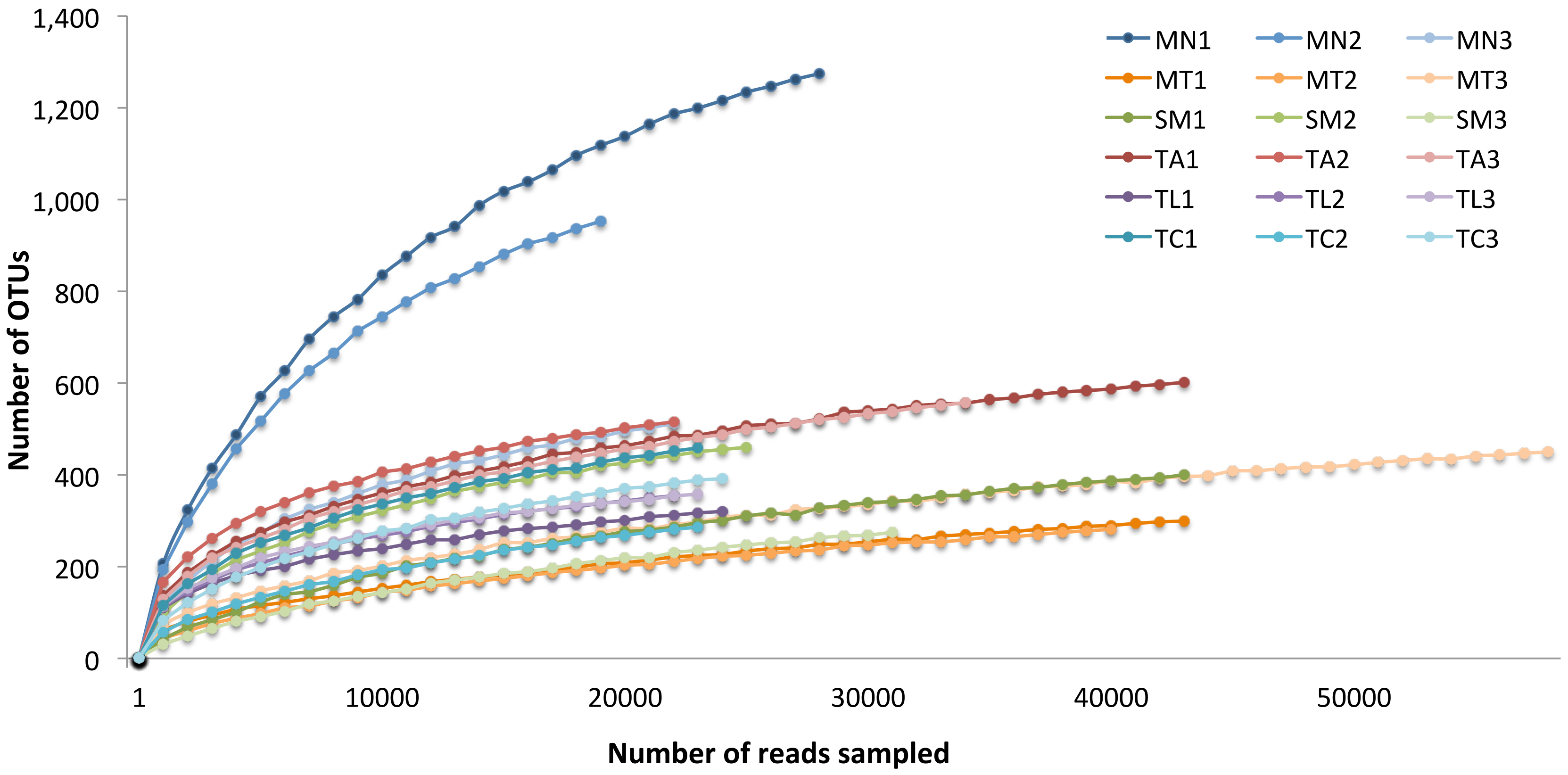

Supplementary Figure S2: Rarefaction curves of OTUs for the coral-associated bacterial community samples from the Gulf of Thailand and Andaman Sea.

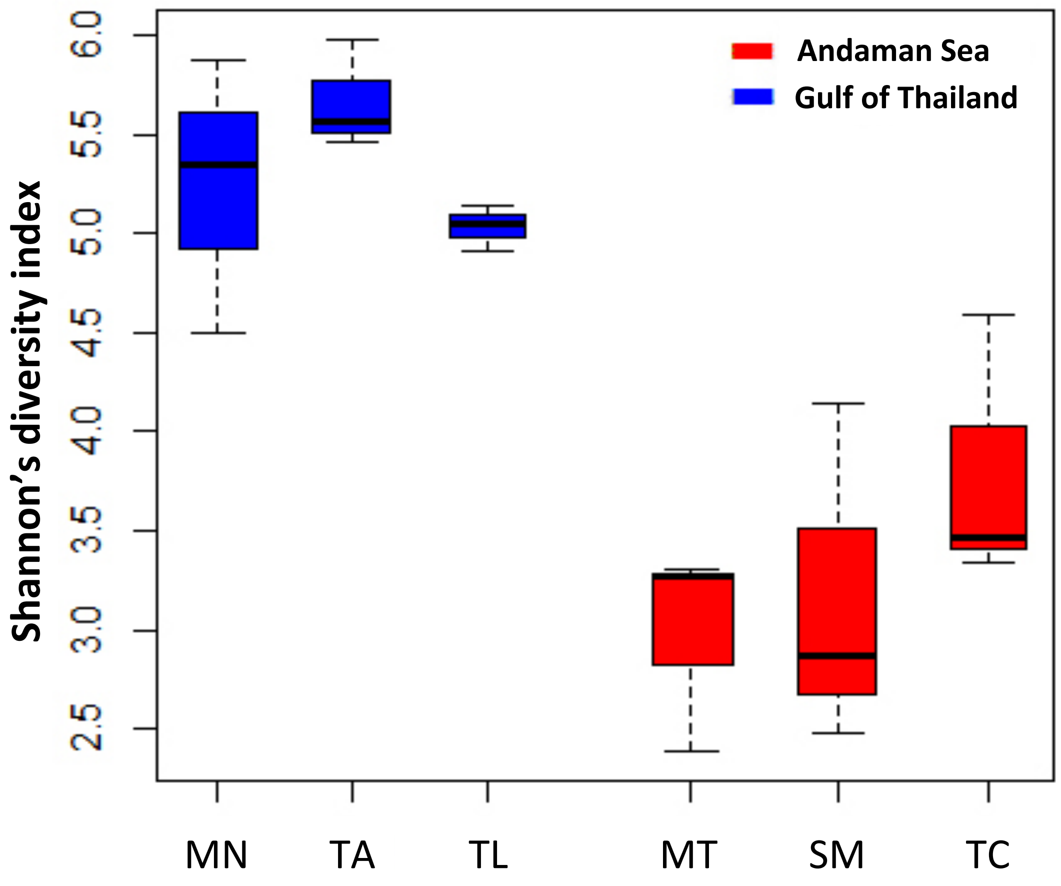

Supplementary Figure S3: Box-plots showing the Shannon diversity indices of coral-associated bacteria from six sampling sites.

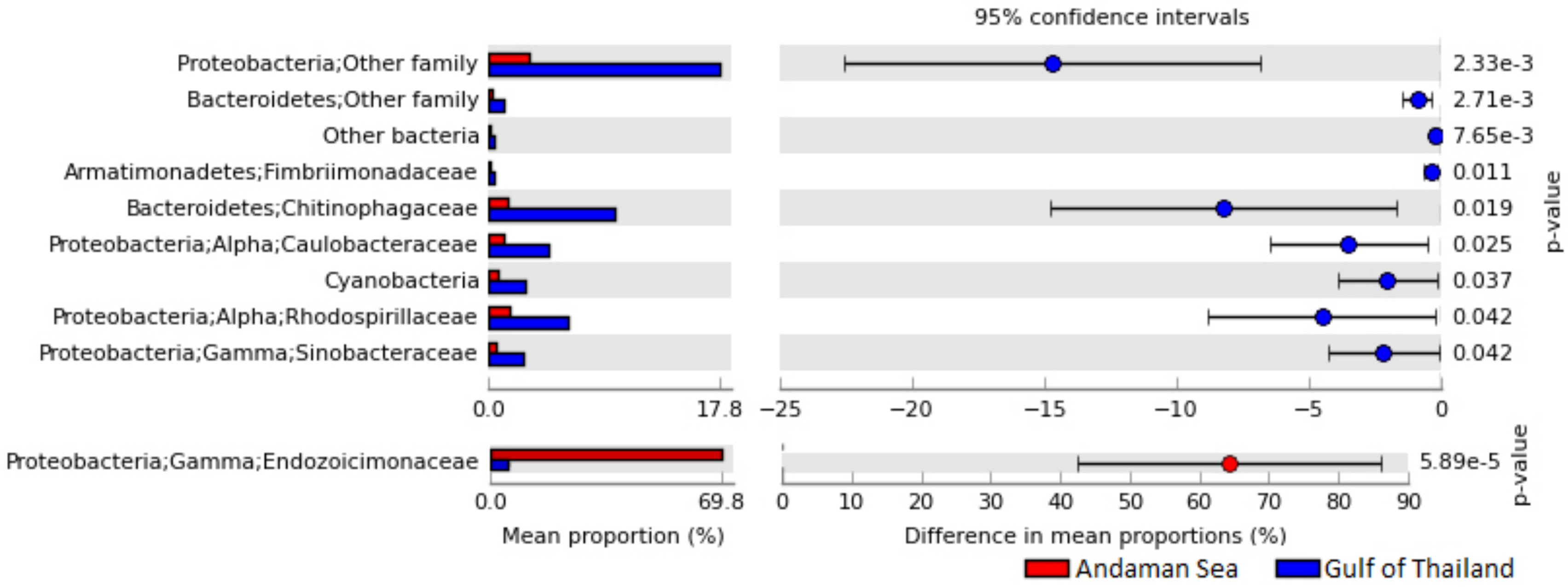

Supplemental Figure S4. Welch's *t*-test based comparative analysis of family abundance in bacterial communities from the Gulf of Thailand and Andaman Sea ($P < 0.05$).

Supplementary Figure S5. Examples of sequence alignment of 16S rRNA genes from closely related bacterial species demonstrating the cases where V3-V4 or V5-V6 regions alone cannot be used for species identification.

```
V3-V4 was highlighted in green
V5-V6 was highlighted in blue
==================================================================================================

CLUSTAL W (1.81) multiple sequence alignment

Alteromonas_mediterranea_S004067      TTAGTTCAGAGTTTGATCATGGCTCAGATTGAACGCTGGCGGCAGGCCTAACACATGCAA
Alteromonas_macleodii_S000014590      ----------------------CTCAGATTGAACGCTGGCGGCAGGCCTAACACATGCAA
Alteromonas_gracilis_S004263718       --------------------TGGCTCAGATTGAACGCTGGCGGCAGGCCTAACACATGCAA
                                                            **************************************

Alteromonas_mediterranea_S004067      GTCGAACGGTAACAGGATTAGCTTGCTAATCGCTGACGAGTGGCGGACGGGTGAGTAATG
Alteromonas_macleodii_S000014590      GTCGAACGGTAACATTTCTAGCTTGCTAGAAGATGACGAGTGGCGGACGGGTGAGTAATG
Alteromonas_gracilis_S004263718       GTCGAACGGTAACATTTCTAGCTTGCTAGAAGATGACGAGTGGCGGACGGGTGAGTAATG
                                      **************    **********   * ***************************

Alteromonas_mediterranea_S004067      CTTGGGAACTTGCCTTTGCGAGGGGGATAACAGTTGGAAACGACTGCTAATACCGCATAA
Alteromonas_macleodii_S000014590      CTTGGGAACTTGCCTTTGCGAGGGGGATAACAGTTGGAAACGACTGCTAATACCGCATAA
Alteromonas_gracilis_S004263718       CTTGGGAACTTGCCTTTGCGAGGGGGATAACAGTTGGAAACGACTGCTAATACCGCATAA
                                      ************************************************************

Alteromonas_mediterranea_S004067      CGTCTACGGACCAAACGGGGCTTAGGCTCCGGCGCAAAGAGAGGCCCAAGTGAGATTAGC
Alteromonas_macleodii_S000014590      TGTCTTCGGACCAAACGGGGCTTCGGCTCCGGCGCAAAGAGAGGCCCAAGTGAGATTAGC
Alteromonas_gracilis_S004263718       TGTCTTCGGACCAAACGGGGCTTCGGCTCCGGCGCAAAGAGAGGCCCAAGTGAGATTAGC
                                       **** ***************** ************************************

Alteromonas_mediterranea_S004067      TAGTTGGTAAGGTAAAGGCTTACCAAGGCAACGATCTCTAGCTGTTCTGAGAGGAAGATC
Alteromonas_macleodii_S000014590      TAGTTGGTAAGGTAACGGCTTACCAAGGCGACGATCTCTAGCTGTTCTGAGAGGAAGATC
Alteromonas_gracilis_S004263718       TAGTTGGTAAGGTAACGGCTTACCAAGGCGACGATCTCTAGCTGTTCTGAGAGGAAGATC
                                      *************** ************* ******************************

Alteromonas_mediterranea_S004067      AGCCACACTGGAACTGAGACACGGTCCAGACTCCTACGGGAGGCAGCAGTGGGGAATATT
Alteromonas_macleodii_S000014590      AGCCACACTGGGACTGAGACACGGCCCAGACTCCTACGGGAGGCAGCAGTGGGGAATATT
Alteromonas_gracilis_S004263718       AGCCACACTGGGACTGAGACACGGCCCAGACTCCTACGGGAGGCAGCAGTGGGGAATATT
                                      *********** ************ ***********************************

Alteromonas_mediterranea_S004067      GCACAATGGGGGAAACCCTGATGCAGCCATGCCGCGTGTGTGAAGAAGGCCTTCGGGTTG
Alteromonas_macleodii_S000014590      GCACAATGGGGGAAACCCTGATGCAGCCATGCCGCGTGTGTGAAGAAGGCCTTCGGGTTG
Alteromonas_gracilis_S004263718       GCACAATGGGGGAAACCCTGATGCAGCCATGCCGCGTGTGTGAAGAAGGCCTTCGGGTTG
                                      ************************************************************
```

```
Alteromonas_mediterranea_S004067       TAAAGCACTTTCAGTTGTGAGGAAAAGTTAGTAGTTAATACCTGCTAGCCGTGACGTTAA
Alteromonas_macleodii_S000014590       TAAAGCACTTTCAGTTGTGAGGAAAAGTTAGTAGTTAATACCTGCTAGCCGTGACGTTAA
Alteromonas_gracilis_S004263718        TAAAGCACTTTCAGTTGTGAGGAAAAGTTAGTAGTTAATACCTGCTAGCCGTGACGTTAA
                                       ************************************************************

Alteromonas_mediterranea_S004067       CAACAGAAGAAGCACCGGCTAACTCCGTGCCAGCAGCCGCGGTAATACGGAGGGTGCGAG
Alteromonas_macleodii_S000014590       CAACAGAAGAAGCACCGGCTAACTCCGTGCCAGCAGCCGCGGTAATACGGAGGGTGCGAG
Alteromonas_gracilis_S004263718        CAACAGAAGAAGCACCGGCTAACTCCGTGCCAGCAGCCGCGGTAATACGGAGGGTGCGAG
                                       ************************************************************

Alteromonas_mediterranea_S004067       CGTTAATCGGAATTACTGGGCGTAAAGCGCACGCAGGCGGTTTGTTAAGCTAGATGTGAA
Alteromonas_macleodii_S000014590       CGTTAATCGGAATTACTGGGCGTAAAGCGCACGCAGGCGGTTTGTTAAGCTAGATGTGAA
Alteromonas_gracilis_S004263718        CGTTAATCGGAATTACTGGGCGTAAAGCGCACGCAGGCGGTTTGTTAAGCTAGATGTGAA
                                       ************************************************************

Alteromonas_mediterranea_S004067       AGCCCCGGGCTCAACCTGGGATGGTCATTTAGAACTGGCAGACTAGAGTCTTGGAGAGGG
Alteromonas_macleodii_S000014590       AGCCCCGGGCTCAACCTGGGATGGTCATTTAGAACTGGCAGACTAGAGTCTTGGAGAGGG
Alteromonas_gracilis_S004263718        AGCCCCGGGCTCAACCTGGGATGGTCATTTAGAACTGGCAGACTAGAGTCTTGGAGAGGG
                                       ************************************************************

Alteromonas_mediterranea_S004067       GAGTGGAATTCCAGGTGTAGCGGTGAAATGCGTAGATATCTGGAGGAACATCAGTGGCGA
Alteromonas_macleodii_S000014590       GAGTGGAATTCCAGGTGTAGCGGTGAAATGCGTAGATATCTGGAGGAACATCAGTGGCGA
Alteromonas_gracilis_S004263718        GAGTGGAATTCCAGGTGTAGCGGTGAAATGCGTAGATATCTGGAGGAACATCAGTGGCGA
                                       ************************************************************

Alteromonas_mediterranea_S004067       AGGCGACTCCCTGGCCAAAGACTGACGCTCATGTGCGAAAGTGTGGGTAGCGAACAGGAT
Alteromonas_macleodii_S000014590       AGGCGACTCCCTGGCCAAAGACTGACGCTCATGTGCGAAAGTGTGGGTAGCGAACAGGAT
Alteromonas_gracilis_S004263718        AGGCGACTCCCTGGCCAAAGACTGACGCTCATGTGCGAAAGTGTGGGTAGCGAACAGGAT
                                       ************************************************************

Alteromonas_mediterranea_S004067       TAGATACCCTGGTAGTCCACACCGTAAACGCTGTCTACTAGCTGTTTG-TGACTTTAAGT
Alteromonas_macleodii_S000014590       TAGATACCCTGGTAGTCCACACCGTAAACGCTGTCTACTAGCTGTGTGTTGTCTCTAAGA
Alteromonas_gracilis_S004263718        TAGATACCCTGGTAGTCCACACCGTAAACGCTGTCTACTAGCTGTGTG-TGCCTTTAAGG
                                       ********************************************* ** ** ** **** 

Alteromonas_mediterranea_S004067       CGTGAGTAGCGAAGCTAACGCGATAAGTAGACCGCCTGGGGAGTACGGCCGCAAGGTTAA
Alteromonas_macleodii_S000014590       CGTGCGTAGCGAAGCTAACGCGCTAAGTAGACCGCCTGGGGAGTACGGCCGCAAGGTTAA
Alteromonas_gracilis_S004263718        CGTGCGTAGCGAAGCTAACGCGATAAGTAGACCGCCTGGGGAGTACGGCCGCAAGGTTAA
                                       **** ***************** *************************************
```

```
Alteromonas_mediterranea_S004067      AACTCAAATGAATTGACGGGGGCCCGCACAAGCGGTGGAGCATGTGGTTTAATTCGATGC
Alteromonas_macleodii_S000014590      AACTCAAATGAATTGACGGGGGCCCGCACAAGCGGTGGAGCATGTGGTTTAATTCGATGC
Alteromonas_gracilis_S004263718       AACTCAAATGAATTGACGGGGGCCCGCACAAGCGGTGGAGCATGTGGTTTAATTCGATGC
                                      ************************************************************

Alteromonas_mediterranea_S004067      AACGCGAAGAACCTTACCTACACTTGACATGCAGAGAACTTTCCAGAGATGGATTGGTGC
Alteromonas_macleodii_S000014590      AACGCGAAGAACCTTACCTACACTTGACATGCTGAGAACTTACTAGAGATAGTTTCGTGC
Alteromonas_gracilis_S004263718       AACGCGAAGAACCTTACCTACACTTGACATGCTGAGAACTTTCTAGAGATAGATTGGTGC
                                      ******************************** ******** * ****** * ** ****

Alteromonas_mediterranea_S004067      CTTCGGGAACTCTGACACAGGTGCTGCATGGCTGTCGTCAGCTCGTGTCGTGAGATGTTG
Alteromonas_macleodii_S000014590      CTTCGGGAACTCAGACACAGGTGCTGCATGGCTGTCGTCAGCTCGTGTCGTGAGATGTTG
Alteromonas_gracilis_S004263718       CTTCGGGAACTCAGACACAGGTGCTGCATGGCTGTCGTCAGCTCGTGTCGTGAGATGTTG
                                      ************ ***********************************************

Alteromonas_mediterranea_S004067      GGTTAAGTCCCGCAACGAGCGCAACCCTTGTCCTTAGTTGCCAGCCTTAAGTTGGGCACT
Alteromonas_macleodii_S000014590      GGTTAAGTCCCGCAACGAGCGCAACCCTTGTCCTTAGTTGCCAGCCTTAAGTTGGGCACT
Alteromonas_gracilis_S004263718       GGTTAAGTCCCGCAACGAGCGCAACCCTTGTCCTTAGTTGCCAGCATTCAGTTGGGCACT
                                      ********************************************* ** ***********

Alteromonas_mediterranea_S004067      CTAAGGAGACTGCCGGTGACAAACCGGAGGAAGGTGGGGACGACGTCAAGTCATCATGGC
Alteromonas_macleodii_S000014590      CTAAGGAGACTGCCGGTGACAAACCGGAGGAAGGTGGGGACGACGTCAAGTCATCATGGC
Alteromonas_gracilis_S004263718       CTAAGGAGACTGCCGGTGACAAACCGGAGGAAGGTGGGGACGACGTCAAGTCATCATGGC
                                      ************************************************************

Alteromonas_mediterranea_S004067      CCTTACGTGTAGGGCTACACACGTGCTACAATGGCATTTACAGAGGGAAGCGAG-ACAGT
Alteromonas_macleodii_S000014590      CCTTACGTGTAGGGCTACACACGTGCTACAATGGCATTTACAGAGGGAAGCGAG-ACAGT
Alteromonas_gracilis_S004263718       CCTTACGTGTAGGGCTACACACGTGCTACAATGGCATTTACAGAGGGAAGCGAGAACAGT
                                      ****************************************************** *****

Alteromonas_mediterranea_S004067      GATGTGGAGCGGACCCCTTAAAGAATGTCGTAGTCCGGATTGGAGTCTGCAACTCGACTC
Alteromonas_macleodii_S000014590      GATGTGGAGCGGACCCCTTAAAGAATGTCGTAGTCCGGATTGGAGTCTGCAACTCGACTC
Alteromonas_gracilis_S004263718       GATGTGGAGCGGACCCCTTAAAGAATGTCGTAGTCCGGATTGGAGTC-GCAACTCGACTC
                                      *********************************************** ************

Alteromonas_mediterranea_S004067      CATGAAGTCGGAATCGCTAGTAATCGCAGGTCAGAATACTGCGGTGAATACGTTCCCGGG
Alteromonas_macleodii_S000014590      CATGAAGTCGGAATCGCTAGTAATCGCAGGTCAGAATACTGCGGTGAATACGTTCCCGGG
Alteromonas_gracilis_S004263718       CATGAAGTCGGAATCGCTAGTAATCGCAGGTCAGAATACTGCGGTGAATACGTTCCCGGG
                                      ************************************************************

Alteromonas_mediterranea_S004067      CCTTGTACACACCGCCCGTCACACCATGGGAGTGGGATGCAAAAGAAGTAGTTAGTCTAA
Alteromonas_macleodii_S000014590      CCTTGTACACACCGCCCGTCACACCATGGGAGTGGGATGCAAAAGAAGTAGTTAGTCTAA
Alteromonas_gracilis_S004263718       CCTTGTACACACCGCCCGTCMCACCATGGGAGT-GGATGCAAAAGAAGTAGTTAGTCTAA
```

```
                                      ******************** ************ **************************

Alteromonas_mediterranea_S004067      CCTTCGGGAGGACGATTACCACTTTGTTCAATTTGTTTCATGACTGGGGTGAAGTCGTAA
Alteromonas_macleodii_S000014590      CCTTCGGGAGGACGATTACCACTTTG-------TGTTTCATGACTGGGGTGAAGTCGTAA
Alteromonas_gracilis_S004263718       CCTTCGGGAGGACGATAACCACTTTG----------------------------------
                                      **************** *********

Alteromonas_mediterranea_S004067      CAAGGTAACCCTAGGGGAACCTGGGGTTGGATCACCTCCTTA
Alteromonas_macleodii_S000014590      CAAGGTAACCCTAGGGGAACCT--------------------
Alteromonas_gracilis_S004263718       ------------------------------------------

================================================================================================

CLUSTAL W (1.81) multiple sequence alignment

Arcobacter_cloacae_S003721995       TAACACATGCAAGTCGAACGAGAACGGGATATAGCTTGCTATATTTGTCAGCTAAGTGGC
Arcobacter_defluvii_S002233781      TAACACATGCAAGTCGAACGAGAACGGATTATAGCTTGCTATAATTGTCAGCTAAGTGGC
                                    ***************************  ************** ****************

Arcobacter_cloacae_S003721995       GCACGGGTGAGTAATGTATAGATAACCTGCCCTTAAGAAAGGAATAACAGTTGGAAACGA
Arcobacter_defluvii_S002233781      GCACGGGTGAGTAATGTATAGATAACCTGCCCTCTAGAAAGGAATAACAGATGGAAACGT
                                    *********************************  *************** ********

Arcobacter_cloacae_S003721995       CTGCTAATGCCCTATATGCCTTTAATACATAAGTATGCAAGGGAAACGCTTTAGTGCTTA
Arcobacter_defluvii_S002233781      CTGCTAATGCCCTATATGCCTTTAATACATAAGTATGCAAGGGAAACGCTTTAGTGCTAG
                                    **********************************************************

Arcobacter_cloacae_S003721995       AGGATGGGTCTGTATGGTATCAGCTTGTTGGTGAGGTAATGGCTCACCAAGGCTATGACG
Arcobacter_defluvii_S002233781      AGGATGGGTCTGTATGGTATCAGCTTGTTGGTGAGGTAATGGCTCACCAAGGCAATGACG
                                    ***************************************************** ******

Arcobacter_cloacae_S003721995       CCTAACTGGTTTGAGAGGATGATCAGTCACACTGGAACTGAGACACGGTCCAGACTCCTA
Arcobacter_defluvii_S002233781      CCTAACTGGTTTGAGAGGATGATCAGTCACACTGGAACTGAGACACGGTCCAGACTCCTA
                                    ************************************************************

Arcobacter_cloacae_S003721995       CGGGAGGCAGCAGTGGGGAATATTGCACAATGGACGAAAGTCTGATGCAGCAACGCCGCG
Arcobacter_defluvii_S002233781      CGGGAGGCAGCAGTGGGGAATATTGCACAATGGACGAAAGTCTGATGCAGCAACGCCGCG
                                    ************************************************************

Arcobacter_cloacae_S003721995       TGGAGGATGACACATTTCGGTGCGTAAACTCCTTTTATATAGGAAGATAATGACGGTACT
Arcobacter_defluvii_S002233781      TGGAGGATGACACATTTCGGTGCGTAAACTCCTTTTATATAGGAAGATAATGACGGTACT
                                    ************************************************************
```

```
Arcobacter_cloacae_S003721995        ATATGAATAAGCACCGGCTAACTCCGTGCCAGCAGCCGCGGTAATACGGAGGGTGCAAGC
Arcobacter_defluvii_S002233781       ATATGAATAAGCACCGGCTAACTCCGTGCCAGCAGCCGCGGTAATACGGAGGGTGCAAGC
                                     ************************************************************

Arcobacter_cloacae_S003721995        GTTACTCGGAATCACTGGGCGTAAAGAGCGTGTAGGCGGGTATATAAGTCAGAAGTGAAA
Arcobacter_defluvii_S002233781       GTTACTCGGAATCACTGGGCGTAAAGAGCGTGTAGGCGGGTATATAAGTCAGAAGTGAAA
                                     ************************************************************

Arcobacter_cloacae_S003721995        TCCAATAGCTTAACTATTGAACTGCTTTTGAAACTGTATACCTAGAATGTGGGAGAGGTA
Arcobacter_defluvii_S002233781       TCCAATAGCTTAACTATTGAACTGCTTTTGAAACTGTATACCTAGAATGTGGGAGAGGTA
                                     ************************************************************

Arcobacter_cloacae_S003721995        GATGGAATTTCTGGTGTAGGGGTAAAATCCGTAGAGATCAGAAGGAATACCGATTGCGAA
Arcobacter_defluvii_S002233781       GATGGAATTTCTGGTGTAGGGGTAAAATCCGTAGAGATCAGAAGGAATACCGATTGCGAA
                                     ************************************************************

Arcobacter_cloacae_S003721995        GGCGATCTACTGGAACATTATTGACGCTGAGACGCGAAAGCGTGGGGAGCAAACAGGATT
Arcobacter_defluvii_S002233781       GGCGATCTACTGGAACATTATTGACGCTGAGACGCGAAAGCGTGGGGAGCAAACAGGATT
                                     ************************************************************

Arcobacter_cloacae_S003721995        AGATACCCTGGTAGTCCACGCCCTAAACGATGTACACTAGTTGTTGTGAGGCTCGACCTT
Arcobacter_defluvii_S002233781       AGATACCCTGGTAGTCCACGCCCTAAACGATGTACACTAGTTGTTGTGAGGCTCGACCTT
                                     ************************************************************

Arcobacter_cloacae_S003721995        GCAGTAATGCAGTTAACACATTAAGTGTACCGCCTGGGGAGTACGGTCGCAAGATTAAAA
Arcobacter_defluvii_S002233781       GCAGTAATGCAGTTAACACATTAAGTGTACCGCCTGGGGAGTACGGTCGCAAGATTAAAA
                                     ************************************************************

Arcobacter_cloacae_S003721995        CTCAAAGGAATAGACGGGGACCCGCACAAGCGGTGGAGCATGTGGTTTAATTCGACGATA
Arcobacter_defluvii_S002233781       CTCAAAGGAATAGACGGGGACCCGCACAAGCGGTGGAGCATGTGGTTTAATTCGACGATA
                                     ************************************************************

Arcobacter_cloacae_S003721995        CACGAAGAACCTTACCTGGACTTGACATAGTAAGAACTTTCTAGAGATAGATTGGTGTCT
Arcobacter_defluvii_S002233781       CACGAAGAACCTTACCTGGACTTGACATAGTAAGAACTTTCTAGAGATAGATTGGTGTCT
                                     ************************************************************

Arcobacter_cloacae_S003721995        GCTTGCAGAAACTTATATACAGGTGCTGCACGGCTGTCGTCAGCTCGTGTCGTGAGATGT
Arcobacter_defluvii_S002233781       GCTTGCAGAAACTTATATACAGGTGCTGCACGGCTGTCGTCAGCTCGTGTCGTGAGATGT
                                     ************************************************************

Arcobacter_cloacae_S003721995        TGGGTTAAGTCCCGCAACGAGCGCAACCCTCGTCATTAGTTGCTAACAGTTCGGCTGAGA
Arcobacter_defluvii_S002233781       TGGGTTAAGTCCCGCAACGAGCGCAACCCTCGTCATTAGTTGCTAACAGTTCGGCTGAGA
                                     ************************************************************
```

```
Arcobacter_cloacae_S003721995       ACTCTAATGAGACTGCCTACGCAAGTAGGAGGAAGGTGAGGACGACGTCAAGTCATCATG
Arcobacter_defluvii_S002233781      ACTCTAATGAGACTGCCTACGCAAGTAGGAGGAAGGTGAGGACGACGTCAAGTCATCATG
                                    ************************************************************

Arcobacter_cloacae_S003721995       GCCCTTACGTCCAGGGCTACACACGTGCTACAATGGGGTATACAAAGAGCAGCAATACGG
Arcobacter_defluvii_S002233781      GCCCTTACGTCCAGGGCTACACACGTGCTACAATGGGGTATACAAAGAGCAGCAATACAG
                                    ********************************************************** *

Arcobacter_cloacae_S003721995       TGACGTGGAGCAAATCTCAAAAATATCTCCCAGTTCGGATTGTAGTCTGCAACTCGACTA
Arcobacter_defluvii_S002233781      TGATGTGGAGCAAATCTCAAAAATATCTCCCAGTTCGGATTGTAGTCTGCAACTCGACTA
                                    *** ********************************************************

Arcobacter_cloacae_S003721995       CATGAAGTTGGAATCGCTAGTAATCGTAGATCAGCTATGCTACGGTGAATACGTTCCCGG
Arcobacter_defluvii_S002233781      CATGAAGTTGGAATCGCTAGTAATCGTAGATCAGCTATGCTACGGTGAATACGTTCCCGG
                                    ************************************************************

Arcobacter_cloacae_S003721995       GTCTTGTACTCACCGCCCGTCACACCATGGGAGTTGAACTCATTCGAAGCGGGGATGCTA
Arcobacter_defluvii_S002233781      GTCTTGTACTCACCGCCCGTCACACCATGGGAGTTGAACTCATTCGAAGCGGGGATGCTA
                                    ************************************************************

Arcobacter_cloacae_S003721995       AAGTAGCTACCTTCCACAGTGG
Arcobacter_defluvii_S002233781      AAGTAGCTACCTTCCACAGTGG
                                    **********************
==================================================================================================
CLUSTAL W (1.81) multiple sequence alignment

Bradyrhizobium_lupini_S000014939      ------------------AGAGTTTGATNNTGGCTCAGAGCGAACGCTGGCGGCAGGCTT
Bradyrhizobium_ganzhouense_S0039      ------------------------------------------------------------
Bradyrhizobium_cytisi_S001743519      TCCGAATTCGTCGACAACAGAGTTTGATCCTGGCTCAGAGCGAACGCTGGCGGCAGGCTT


Bradyrhizobium_lupini_S000014939      AACACATGCAAGTCGAGCGGGCGTAGCAATACGTCAGCGGCAGACGGGTGAGTAACGCGT
Bradyrhizobium_ganzhouense_S0039      -----ATGCAAGTCGAGCGGGCGTAGCAATACGTCAGCGGCAGACGGGTGAGTAACGCGT
Bradyrhizobium_cytisi_S001743519      AACACATGCAAGTCGAGCGGGCGTAGCAATACGTCAGCGGCAGACGGGTGAGTAACGCGT
                                           *******************************************************

Bradyrhizobium_lupini_S000014939      GGGAACGTACCTTTTGGTTCGGAACAACACAGGGAAACTTGTGCTAATACCGGATAAGCC
Bradyrhizobium_ganzhouense_S0039      GGGAACGTACCTTTTGGTTCGGAACAACACAGGGAAACTTGTGCTAATACCGGATAAGCC
Bradyrhizobium_cytisi_S001743519      GGGAACATACCTTTTGGTTCGGAACAACACAGGGAAACTTGTGCTAATACCGGATAAGCC
                                      ****** *****************************************************

Bradyrhizobium_lupini_S000014939      CTTACGGGGAAAGATTTATCGCCGAAAGATCGGCCCGCGTCTGATTAGCTAGTTGGTGAG
```

```
Bradyrhizobium_ganzhouense_S0039      CTTACGGGGAAAGATTTATCGCCGAAAGATCGGCCCGCGTCTGATTAGCTAGTTGGTAGG
Bradyrhizobium_cytisi_S001743519      CTTACGGGGAAAGATTTATCGCCGAAAGATTGGCCCGCGTCTGATTAGCTAGTTGGTAGG
                                      ****************************** **************************  *

Bradyrhizobium_lupini_S000014939      GTAATGGCTCACCAAGGCGACGATCAGTAGCTGGTCTGAGAGGATGATCAGCCACATTGG
Bradyrhizobium_ganzhouense_S0039      GTAATGGCCTACCAAGGCGACGATCAGTAGCTGGTCTGAGAGGATGATCAGCCACATTGG
Bradyrhizobium_cytisi_S001743519      GTAATGGCCTACCAAGGCGACGATCAGTAGCTGGTCTGAGAGGATGATCAGCCACATTGG
                                      ********  **************************************************

Bradyrhizobium_lupini_S000014939      GACTGAGACACGGCCCAAACTCCTACGGGAGGCAGCAGTGGGGAATATTGGACAATGGGG
Bradyrhizobium_ganzhouense_S0039      GACTGAGACACGGCCCAAACTCCTACGGGAGGCAGCAGTGGGGAATATTGGACAATGGGG
Bradyrhizobium_cytisi_S001743519      GACTGAGACACGGCCCAAACTCCTACGGGAGGCAGCAGTGGGGAATATTGGACAATGGGG
                                      ************************************************************

Bradyrhizobium_lupini_S000014939      GCAACCCTGATCCAGCCATGCCGCGTGAGTGATGAAGGCCCTAGGGTTGTAAAGCTCTTT
Bradyrhizobium_ganzhouense_S0039      GCAACCCTGATCCAGCCATGCCGCGTGAGTGATGAAGGCCCTAGGGTTGTAAAGCTCTTT
Bradyrhizobium_cytisi_S001743519      GCAACCCTGATCCAGCCATGCCGCGTGAGTGATGAAGGCCCTAGGGTTGTAAAGCTCTTT
                                      ************************************************************

Bradyrhizobium_lupini_S000014939      TGTGCGGGAAGATAATGACGGTACCGCAAGAATAAGCCCCGGCTAACTTCGTGCCAGCAG
Bradyrhizobium_ganzhouense_S0039      TGTGCGGGAAGATAATGACGGTACCGCAAGAATAAGCCCCGGCTAACTTCGTGCCAGCAG
Bradyrhizobium_cytisi_S001743519      TGTGCGGGAAGATAATGACGGTACCGCAAGAATAAGCCCCGGCTAACTTCGTGCCAGCAG
                                      ************************************************************

Bradyrhizobium_lupini_S000014939      CCGCGGTAATACGAAGGGGGCTAGCGTTGCTCGGAATCACTGGGCGTAAAGGGTGCGTAG
Bradyrhizobium_ganzhouense_S0039      CCGCGGTAATACGAAGGGGGCTAGCGTTGCTCGGAATCACTGGGCGTAAAGGGTGCGTAG
Bradyrhizobium_cytisi_S001743519      CCGCGGTAATACGAAGGGGGCTAGCGTTGCTCGGAATCACTGGGCGTAAAGGGTGCGTAG
                                      ************************************************************

Bradyrhizobium_lupini_S000014939      GCGGGTCTTTAAGTCAGGGGTGAAATCCTGGAGCTCAACTCCAGAACTGCCTTTGATACT
Bradyrhizobium_ganzhouense_S0039      GCGGGTTTTTAAGTCAGGGGTGAAATCCTGGAGCTCAACTCCAGAACTGCCTTTGATACT
Bradyrhizobium_cytisi_S001743519      GCGGGTTTTTAAGTCAGGGGTGAAATCCTGGAGCTCAACTCCAGAACTGCCTTTGATACT
                                      ****** *****************************************************

Bradyrhizobium_lupini_S000014939      GAGGATCTTGAGTTCGGGAGAGGTGAGTGGAACTGCGAGTGTAGAGGTGAAATTCGTAGA
Bradyrhizobium_ganzhouense_S0039      GAAGATCTTGAGTCCGGGAGAGGTGAGTGGAACTGCGAGTGTAGAGGTGAAATTCGTAGA
Bradyrhizobium_cytisi_S001743519      GAAGATCTTGAGTCCGGGAGAGGTGAGTGGAACTGCGAGTGTAGAGGTGAAATTCGTAGA
                                      ** ********** **********************************************

Bradyrhizobium_lupini_S000014939      TATTCGCAAGAACACCAGTGGCGAAGGCGGCTCACTGGCCCGATACTGACGCTGAGGCAC
Bradyrhizobium_ganzhouense_S0039      TATTCGCAAGAACACCAGTGGCGAAGGCGGCTCACTGGCCCGGTACTGACGCTGAGGCAC
Bradyrhizobium_cytisi_S001743519      TATTCGCAAGAACACCAGTGGCGAAGGCGGCTCACTGGCCCGGTACTGACGCTGAGGCAC
                                      ***************************************** ******************
```

```
Bradyrhizobium_lupini_S000014939      GAAAGCGTGGGGAGCAAACAGGATTAGATACCCTGGTAGTCCACGCCGTAAACGATGAAT
Bradyrhizobium_ganzhouense_S0039      GAAAGCGTGGGGAGCAAACAGGATTAGATACCCTGGTAGTCCACGCCGTAAACGATGAAT
Bradyrhizobium_cytisi_S001743519      GAAAGCGTGGGGAGCAAACAGGATTAGATACCCTGGTAGTCCACGCCGTAAACGATGAAT
                                      ************************************************************

Bradyrhizobium_lupini_S000014939      GCCAGCCGTTAGTGGGTTTACTCACTAGTGGCGCAGCTAACGCTTTAAGCATTCCGCCTG
Bradyrhizobium_ganzhouense_S0039      GCCAGCCGTTAGTGGGTTTACTCACTAGTGGCGCAGCTAACGCTTTAAGCATTCCGCCTG
Bradyrhizobium_cytisi_S001743519      GCCAGCCGTTAGTGGGTTTACTCACTAGTGGCGCAGCTAACGCTTTAAGCATTCCGCCTG
                                      ************************************************************

Bradyrhizobium_lupini_S000014939      GGGAGTACGGTCGCAAGATTAAAACTCAAAGGAATTGACGGGGGCCCGCACAAGCGGTGG
Bradyrhizobium_ganzhouense_S0039      GGGAGTACGGTCGCAAGATTAAAACTCAAAGGAATTGACGGGGGCCCGCACAAGCGGTGG
Bradyrhizobium_cytisi_S001743519      GGGAGTACGGTCGCAAGATTAAAACTCAAAGGAATTGACGGGGGCCCGCACAAGCGGTGG
                                      ************************************************************

Bradyrhizobium_lupini_S000014939      AGCATGTGGTTTAATTCGACGCAACGCGCAGAACCTTACCAGCCCTTGACATGTCCAGGA
Bradyrhizobium_ganzhouense_S0039      AGCATGTGGTTTAATTCGACGCAACGCGCAGAACCTTACCAGCCCTTGACATGTCCAGGA
Bradyrhizobium_cytisi_S001743519      AGCATGTGGTTTAATTCGACGCAACGCGCAGAACCTTACCAGCCCTTGACATGTCCAGGA
                                      ************************************************************

Bradyrhizobium_lupini_S000014939      CCGGTCGCAGAGATGTGACCTTCTCTTCGGAGCCTGGAGCACAGGTGCTGCATGGCTGTC
Bradyrhizobium_ganzhouense_S0039      CCGGTCGCAGAGATGTGACCTTCTCTTCGGAGCCTGGAACACAGGTGCTGCATGGCTGTC
Bradyrhizobium_cytisi_S001743519      CCGGTCGCAGAGATGTGACCTTCTCTTCGGAGCCTGGAGCACAGGTGCTGCATGGCTGTC
                                      ************************************** *********************

Bradyrhizobium_lupini_S000014939      GTCAGCTCGTGTCGTGAGATGTTGGGTTAAGTCCCGCAACGAGCGCAACCCCCGTCCTTA
Bradyrhizobium_ganzhouense_S0039      GTCAGCTCGTGTCGTGAGATGTTGGGTTAAGTCCCGCAACGAGCGCAACCCCCGTCCTTA
Bradyrhizobium_cytisi_S001743519      GTCAGCTCGTGTCGTGAGATGTTGGGTTAAGTCCCGCAACGAGCGCAACCCCCGTCCTTA
                                      ************************************************************

Bradyrhizobium_lupini_S000014939      GTTGCTACCATTTAGTTGAGCACTCTAAGGAGACTGCCGGTCATAAGCCGCGAGGAAGGT
Bradyrhizobium_ganzhouense_S0039      GTTGCTACCATTTAGTTGAGCACTCTAAGGAGACTGCCGGTGATAAGCCGCGAGGAAGGT
Bradyrhizobium_cytisi_S001743519      GTTGCTACCATTTAGTTGAGCACTCTAAGGAGACTGCCGGTGATAAGCCGCGAGGAAGGT
                                      ***************************************** ******************

Bradyrhizobium_lupini_S000014939      GGGGATGACGTCAAGTCCTCATGGCCCTTACGGGCTGGGCTACACACGTGCTACAATGGC
Bradyrhizobium_ganzhouense_S0039      GGGGATGACGTCAAGTCCTCATGGCCCTTACGGGCTGGGCTACACACGTGCTACAATGGC
Bradyrhizobium_cytisi_S001743519      GGGGATGACGTCAAGTCCTCATGGCCCTTACGGGCTGGGCTACACACGTGCTACAATGGC
                                      ************************************************************

Bradyrhizobium_lupini_S000014939      GGTGACAATGGGATGCTAAGGGGCGACCCTTCGCAAATCTCAAAAAGCCGTCTCAGTTCG
Bradyrhizobium_ganzhouense_S0039      GGTGACAATGGGATGCTAAGGGGCGACCCTTCGCAAATCTCAAAAAGCCGTCTCAGTTCG
```

```
Bradyrhizobium_cytisi_S001743519      GGTGACAATGGGACGCTAAGGGGCAACCCTTCGCAAATCTCAAAAAGCCGTCTCAGTTCG
                                      ************* ********** ***********************************

Bradyrhizobium_lupini_S000014939      GATTGGGCTCTGCAACTCGAGCCCATGAAGTTGGAATCGCTAGTAATCGTGGATCAGCAC
Bradyrhizobium_ganzhouense_S0039      GATTGGGCTCTGCAACTCGAGCCCATGAAGTTGGAATCGCTAGTAATCGTGGATCAGCAC
Bradyrhizobium_cytisi_S001743519      GATTGGGCTCTGCAACTCGAGCCCATGAAGTTGGAATCGCTAGTAATCGTGGATCAGCAC
                                      ************************************************************

Bradyrhizobium_lupini_S000014939      GCCACGGTGAATACGTTCCCGGGCCTTGTACACACCGCCCGTCACACCATGGGAGTTGGT
Bradyrhizobium_ganzhouense_S0039      GCCACGGTGAATACGTTCCCGGGCCTTGTACACACCGCCCGTCACACCATGGGAGTTGGT
Bradyrhizobium_cytisi_S001743519      GCCACGGTGAATACGTTCCCGGGCCTTGTACACACCGCCCGTCACACCATGGGAGTTGGT
                                      ************************************************************

Bradyrhizobium_lupini_S000014939      TTTACCTGAAGACGGTGCGCTAACCCGCAAGGGAGGCAGCCGGCCACGGTAGGGTCAGCG
Bradyrhizobium_ganzhouense_S0039      TTTACCTGAAGACGGTGCGCTAACCAGCAATGGAGGCAGCCGGCCACG------------
Bradyrhizobium_cytisi_S001743519      TTTACCTGAAGACGGTGCGCTAACCAGCAATGGAGGCAGCCGGCCACGGTAGGGTCAGCG
                                      ************************* **** *****************

Bradyrhizobium_lupini_S000014939      ACTGGGGTGAAGTCGTAACAAGGTAGCCGTAGGGGAACCTGCGGCTGGATCACCTCCTTT
Bradyrhizobium_ganzhouense_S0039      ------------------------------------------------------------
Bradyrhizobium_cytisi_S001743519      ACTGGGGTGAAGTCGTAACAAGGTAGCCGTAGGGGAACCTGCGGCTGGATCACCTCCTTA


Bradyrhizobium_lupini_S000014939      NN--------------
Bradyrhizobium_ganzhouense_S0039      ----------------
Bradyrhizobium_cytisi_S001743519      AGCTTGGATCCCGGGA

==================================================================================================
CLUSTAL W (1.81) multiple sequence alignment

Burkholderia_fungorum_S000389260      -------------------------------GCGGCATGCCTTACACATGCAAGTCGAAC
Burkholderia_phytofirmans_S00054      AGAGTTTGATCCTGGCTCAGATTGAACGCTGGCGGCATGCCTTACACATGCAAGTCGAAC
                                                                     *****************************

Burkholderia_fungorum_S000389260      GGCAGCACGGGGGCAA-CCCTGGTGGCGAGTGGCGAACGGGTGAGTAATACATCGGAACG
Burkholderia_phytofirmans_S00054      GGCAGCACGGGGGCAACCCCTGGTGGCGAGTGGCGAACGGGTGAGTAATACATCGGAACG
                                      **************** *******************************************

Burkholderia_fungorum_S000389260      TGTCCTGTAGTGGGGGATAGCCCGGCGAAAGCCGGATTAATACCGCATACGCTCTACGGA
Burkholderia_phytofirmans_S00054      TGTCCTGTAGTGGGGGATAGCCCGGCGAAAGCCGGATTAATACCGCATACGATCTGTGGA
                                      *************************************************** ***  ***

Burkholderia_fungorum_S000389260      GGAAAGGGGGGGATCTTAGGACCTCCCGCTACAGGGGCGGCCGATGGCAGATTAGCTAGT
```

```
Burkholderia_phytofirmans_S00054      TGAAAGCGGGGGATCTTAGGACCTCGCGCTACAGGGGCGGCCGATGGCAGATTAGCTAGT
                                       ***** ****************** **********************************

Burkholderia_fungorum_S000389260      TGGTGGGGTAAAGGCCTACCAAGGCGACGATCTGTAGCTGGTCTGAGAGGACGACCAGCC
Burkholderia_phytofirmans_S00054      TGGTGGGGTAAAGGCCTACCAAGGCGACGATCTGTAGCTGGTCTGAGAGGACGACCAGCC
                                      ************************************************************

Burkholderia_fungorum_S000389260      ACACTGGGACTGAGACACGGCCCAGACTCCTACGGGAGGCAGCAGTGGGGAATTTTGGAC
Burkholderia_phytofirmans_S00054      ACACTGGGACTGAGACACGGCCCAGACTCCTACGGGAGGCAGCAGTGGGGAATTTTGGAC
                                      ************************************************************

Burkholderia_fungorum_S000389260      AATGGGGGCAACCCTGATCCAGCAATGCCGCGTGTGTGAAGAAGGCCTTCGGGTTGTAAA
Burkholderia_phytofirmans_S00054      AATGGGCGCAAGCCTGATCCAGCAATGCCGCGTGTGTGAAGAAGGCCTTCGGGTTGTAAA
                                      ****** **** ************************************************

Burkholderia_fungorum_S000389260      GCACTTTTGTCCGGAAAGAAAACGCCGTGGTTAATACCCGTGGCGGATGACGGTACCGGA
Burkholderia_phytofirmans_S00054      GCACTTTTGTCCGGAAAGAAAACCTCTTGGTTAATACCTGAGGGGGATGACGGTACCGGA
                                      ***********************  * *********** * ** ****************

Burkholderia_fungorum_S000389260      AGAATAAGCACCGGCTAACTACGTGCCAGCAGCCGCGGTAATACGTAGGGTGCAAGCGTT
Burkholderia_phytofirmans_S00054      AGAATAAGCACCGGCTAACTACGTGCCAGCAGCCGCGGTAATACGTAGGGTGCAAGCGTT
                                      ************************************************************

Burkholderia_fungorum_S000389260      AATCGGAATTACTGGGCGTAAAGCGTGCGCAGGCGGTCCGCTAAGACAGATGTGAAATCC
Burkholderia_phytofirmans_S00054      AATCGGAATTACTGGGCGTAAAGCGTGCGCAGGCGGTTCGCTAAGACAGATGTGAAATCC
                                      ************************************* **********************

Burkholderia_fungorum_S000389260      CCGGGCTTAACCTGGGAACTGCATTTGTGACTGGCGGGCTAGAGTATGGCAGAGGGGGGT
Burkholderia_phytofirmans_S00054      CCGGGCTTAACCTGGGAACTGCATTTGTGACTGGCGGGCTAGAGTATGGCAGAGGGGGGT
                                      ************************************************************

Burkholderia_fungorum_S000389260      AGAATTCCACGTGTAGCAGTGAAATGCGTAGAGATGTGGAGGAATACCGATGGCGAAGGC
Burkholderia_phytofirmans_S00054      AGAATTCCACGTGTAGCAGTGAAATGCGTAGAGATGTGGAGGAATACCGATGGCGAAGGC
                                      ************************************************************

Burkholderia_fungorum_S000389260      AGCCCCCTGGGCCAATACTGACGCTCATGCACGAAAGCGTGGGGAGCAAACAGGATTAGA
Burkholderia_phytofirmans_S00054      AGCCCCCTGGGCCAATACTGACGCTCATGCACGAAAGCGTGGGGAGCAAACAGGATTAGA
                                      ************************************************************

Burkholderia_fungorum_S000389260      TACCCTGGTAGTCCACGCCCTAAACGATGTCAACTAGTTGTCGGGTCTTCATTGACTTGG
Burkholderia_phytofirmans_S00054      TACCCTGGTAGTCCACGCCCTAAACGATGTCAACTAGTTGTCGGGTCTTCATTGACTTGG
                                      ************************************************************
```

```
Burkholderia_fungorum_S000389260      TAACGTAGCTAACGCGTGAAGTTGACCGCCTGGGGAGTACGGTCGCAAGATTAAAACTCA
Burkholderia_phytofirmans_S00054      TAACGTAGCTAACGCGTGAAGTTGACCGCCTGGGGAGTACGGTCGCAAGATTAAAACTCA
                                      ************************************************************

Burkholderia_fungorum_S000389260      AAGGAATTGACGGGGACCCGCACAAGCGGTGGATGATGTGGATTAATTCGATGCAACGCG
Burkholderia_phytofirmans_S00054      AAGGAATTGACGGGGACCCGCACAAGCGGTGGATGATGTGGATTAATTCGATGCAACGCG
                                      ************************************************************

Burkholderia_fungorum_S000389260      AAAAACCTTACCTACCCTTGACATGTATGGAATCCTGCTGAGAGGTGGGAGTGCCCGAAA
Burkholderia_phytofirmans_S00054      AAAAACCTTACCTACCCTTGACATGTATGGAATCCTGCTGAGAGGTGGGAGTGCCCGAAA
                                      ************************************************************

Burkholderia_fungorum_S000389260      GGGAGCCATAACACAGGTGCTGCATGGCTGTCGTCAGCTCGTGTCGTGAGATGTTGGGTT
Burkholderia_phytofirmans_S00054      GGGAGCCATAACACAGGTGCTGCATGGCTGTCGTCAGCTCGTGTCGTGAGATGTTGGGTT
                                      ************************************************************

Burkholderia_fungorum_S000389260      AAGTCCCGCAACGAGCGCAACCCTTGTCCCTAGTTGCTACGCAAGAGCACTCTAGGGAGA
Burkholderia_phytofirmans_S00054      AAGTCCCGCAACGAGCGCAACCCTTGTCCCTAGTTGCTACGCAAGAGCACTCTAGGGAGA
                                      ************************************************************

Burkholderia_fungorum_S000389260      CTGCCGGTGACAAACCGGAGGAAGGTGGGGATGACGTCAAGTCCTCATGGCCCTTATGGG
Burkholderia_phytofirmans_S00054      CTGCCGGTGACAAACCGGAGGAAGGTGGGGATGACGTCAAGTCCTCATGGCCCTTATGGG
                                      ************************************************************

Burkholderia_fungorum_S000389260      TAGGGCTTCACACGTCATACAATGGTCGGAACAGAGGGTCGCCAACCCGCGAGGGGGAGC
Burkholderia_phytofirmans_S00054      TAGGGCTTCACACGTCATACAATGGTCGGAACAGAGGGTCGCCAACCCGCGAGGGGGAGC
                                      ************************************************************

Burkholderia_fungorum_S000389260      CAATCCCAGAAAACCGATCGTAGTCCGGATCGCACTCTGCAACTCGAGTGCGTGAAGCTG
Burkholderia_phytofirmans_S00054      CAATCCCAGAAAACCGATCGTAGTCCGGATCGCACTCTGCAACTCGAGTGCGTGAAGCTG
                                      ************************************************************

Burkholderia_fungorum_S000389260      GAATCGCTAGTAATCGCGGATCAGCATGCCGCGGTGAATACGTTCCCGGGTCTTGTACAC
Burkholderia_phytofirmans_S00054      GAATCGCTAGTAATCGCGGATCAGCATGCCGCGGTGAATACGTTCCCGGGTCTTGTACAC
                                      ************************************************************

Burkholderia_fungorum_S000389260      ACCGCCCGTCACACCATGGGAGTGGGTTTTACCAGAAGTGGCTAGTCTAACCGCAAGGAG
Burkholderia_phytofirmans_S00054      ACCGCCCGTCACACCATGGGAGTGGGTTTTACCAGAAGTGGCTAGTCTAACCGCAAGGAG
                                      ************************************************************

Burkholderia_fungorum_S000389260      GACGGTCACCACGGTAGGATTCATGACTGGGGTGAAGTCGTAACAAGGTAGCCGTATCGG
Burkholderia_phytofirmans_S00054      GACGGTCACCACGGTAGGATTCATGACTGGGGTGAAGTCG-AACAAGGA-----------
                                      **************************************** *******
```

```
Burkholderia_fungorum_S000389260      AAGG
Burkholderia_phytofirmans_S00054      ----

=============================================================================================================
CLUSTAL W (1.81) multiple sequence alignment

Epibacterium_ulvae_S003278975      -----------------------------GC-AGTCGAGCGCGCCCTTCGGGGTGAGCGG
Shimia_marina_S000515801           AACGAACGCTGGCGGCAGGCCTAACACATGCAAGTCGAGCGCGCCCTTCGGGGTGAGCGG
                                                                ** ****************************


Epibacterium_ulvae_S003278975      CGGACGGGTTAGTAACGCGTGGGAATATACCCTTTGGTAAGGAATAGCCTCTGGAAACGG
Shimia_marina_S000515801           CGGACGGGTTAGTAACGCGTGGGAACGTACCCAGATCTACGGAATAGCCTTTGGAAACGA
                                   *************************  *****     ** ********** ******** 

Epibacterium_ulvae_S003278975      AGAGTAATACCTTATGTGCCCTTCGGGGGAAAGATTTATCGCCAAAGGATTAGCCCGCGT
Shimia_marina_S000515801           AGAGTAATACCGTATACGCCCTTCGGGGGAAAGATTTATCGGATTTGGATCGGCCCGCGT
                                   *********** ***  ************************     ****  ********

Epibacterium_ulvae_S003278975      TAGATTAGATAGTTGGTGGGGTAATGGCCTACCAAGTCTACGATCTATAGCTGGTTTTAG
Shimia_marina_S000515801           TAGATTAGATAGTTGGTGGGGTAACGGCCTACCAAGTCTACGATCTATAGCTGGTTTTAG
                                   ************************ ***********************************

Epibacterium_ulvae_S003278975      AGGATGATCAGCAACACTGGGACTGAGACACGGCCCAGACTCCTACGGGAGGCAGCAGTG
Shimia_marina_S000515801           AGGATGATCAGCAACACTGGGACTGAGACACGGCCCAGACTCCTACGGGAGGCAGCAGTG
                                   ************************************************************

Epibacterium_ulvae_S003278975      GGGAATCTTGGACAATGGGGGCAACCCTGATCCAGCCATGCCGCGTGAGCGATGAAGGCC
Shimia_marina_S000515801           GGGAATCTTGGACAATGGGCGCAAGCCTGATCCAGCCATGCCGCGTGAGTGATGAAGGCC
                                   ******************* **** ************************ **********

Epibacterium_ulvae_S003278975      TTAGGGTCGTAAAACTCTTTCGCCAGAGATGATAATGACAGTATCTGGTAAAGAAACCCC
Shimia_marina_S000515801           TTAGGGTCGTAAAGCTCTTTCGCCAGGGATGATAATGACAGTACCTGGTAAAGAAACCCC
                                   ************* ************ **************** ****************

Epibacterium_ulvae_S003278975      GGCTAACTCCGTGCCAGCAGCCGCGGTAATACGGAGGGGGTTAGCGTTGTTCGGAATTAC
Shimia_marina_S000515801           GGCTAACTCCGTGCCAGCAGCCGCGGTAATACGGAGGGGGTTAGCGTTGTTCGGAATTAC
                                   ************************************************************

Epibacterium_ulvae_S003278975      TGGGCGTAAAGCGCACGTAGGCGGATTGGAAAGTTAGAGGTGAAATCCCGGGGCTCAACC
Shimia_marina_S000515801           TGGGCGTAAAGCGCGCGTAGGCGGGTTGTTAAGTTAGAGGTGAAATCCCGGGGCTCAACC
                                   ************** ********* ***  ******************************
```

```
Epibacterium_ulvae_S003278975      CCGGAACTGCCTTTAAAACTCCCAGTCTAGAGTTCGAGAGAGGTGAGTGGAATTCCGAGT
Shimia_marina_S000515801           CCGGAACTGCCTTTAATACTGGCAACCTAGAGTTCGAGAGAGGTGAGTGGAATTCCGAGT
                                   **************** ***  **  **********************************

Epibacterium_ulvae_S003278975      GTAGAGGTGAAATTCGTAGATATTCGGAGGAACACCAGTGGCGAAGGCGGCTCACTGGCT
Shimia_marina_S000515801           GTAGAGGTGAAATTCGTAGATATTCGGAGGAACACCAGTGGCGAAGGCGGCTCACTGGCT
                                   ************************************************************

Epibacterium_ulvae_S003278975      CGATACTGACGCTGAGGTGCGAAAGTGTGGGGAGCAAACAGGATTAGATACCCTGGTAGT
Shimia_marina_S000515801           CGATACTGACGCTGAGGTGCGAAAGTGTGGGGAGCAAACAGGATTAGATACCCTGGTAGT
                                   ************************************************************

Epibacterium_ulvae_S003278975      CCACACCGTAAACGATGAATGCCAGTCGTCGGGTAGCATGCTATTCGGTGACACACCTAA
Shimia_marina_S000515801           CCACACCGTAAACGATGAATGCCAGTCGTCGGGTAGCATGCTATTCGGTGACACACCTAA
                                   ************************************************************

Epibacterium_ulvae_S003278975      CGGATTAAGCATTCCGCCTGGGGAGTACGGTCGCAAGATTAAAACTCAAAGGAATTGACG
Shimia_marina_S000515801           CGGATTAAGCATTCCGCCTGGGGAGTACGGTCGCAAGATTAAAACTCAAAGGAATTGACG
                                   ************************************************************

Epibacterium_ulvae_S003278975      GGGGCCCGCACAAGCGGTGGAGCATGTGGTTTAATTCGAAGCAACGCGCAGAACCTTACC
Shimia_marina_S000515801           GGGGCCCGCACAAGCGGTGGAGCATGTGGTTTAATTCGAAGCAACGCGCAGAACCTTACC
                                   ************************************************************

Epibacterium_ulvae_S003278975      AACCCTTGACATCCTAGGACAACTCCAGAGATGGAGCCTTCCTTCGGGACCTAGTGACAG
Shimia_marina_S000515801           AACCCTTGACATCCTAGGACAACTCCAGAGATGGAGCCTTCCTTCGGGACCTAGTGACAG
                                   ************************************************************

Epibacterium_ulvae_S003278975      GTGCTGCATGGCTGTCGTCAGCTCGTGTCGTGAGATGTTCGGTTAAGTCCGGCAACGAGC
Shimia_marina_S000515801           GTGCTGCATGGCTGTCGTCAGCTCGTGTCGTGAGATGTTCGGTTAAGTCCGGCAACGAGC
                                   ************************************************************

Epibacterium_ulvae_S003278975      GCAACCCACATCTTTAGTTGCCAGCAGTTCGGCTGGGCACTCTAGAGAAACTGCCCGTGA
Shimia_marina_S000515801           GCAACCCACATCCTTAGTTGCCAGCAGTTCGGCTGGGCACTCTAGGGAAACTGCCCGTGA
                                   ************ ******************************** **************

Epibacterium_ulvae_S003278975      TAAGCGGGAGGAAGGTGTGGATGACGTCAAGTCCTCATGGCCCTTACGGGTTGGGCTACA
Shimia_marina_S000515801           TAAGCGGGAGGAAGGTGTGGATGACGTCAAGTCCTCATGGCCCTTACGGGTTGGGCTACA
                                   ************************************************************

Epibacterium_ulvae_S003278975      CACGTGCTACAATGGCATCTACAGTGGGTTAATCCCCAAAAGATGTCTCAGTTCGGATTG
Shimia_marina_S000515801           CACGTGCTACAATGGCAGTGACAATGGGTTAATCCCCAAAAACTGTCTCAGTTCGGATTG
```

```
                                    *****************   *** *****************  *****************

Epibacterium_ulvae_S003278975       TTCTCTGCAACTCGAGAGCATGAAGTCGGAATCGCTAGTAATCGCGTAACAGCATGACGC
Shimia_marina_S000515801            GGGTCTGCAACTCGACCCCATGAAGTCGGAATCGCTAGTAATCGCGTAACAGCATGACGC
                                       ************   ******************************************

Epibacterium_ulvae_S003278975       GGTGAATACGTTCCCGGGCCTTGTACACACCGCCCGTCACACCATGGGAGTTGGGTCTAC
Shimia_marina_S000515801            GGTGAATACGTTCCCGGGCCTTGTACACACCGCCCGTCACACCATGGGAGTTGGTTCTAC
                                    ****************************************************** *****


Epibacterium_ulvae_S003278975       CCGACGGCCGTGCGCCAACCTTCGAGGGGCATCGAC------------------------
Shimia_marina_S000515801            CCGACGGCCGTGCGCTAACCTTTTGGAGGCAGCGGACCACGGTAGGATCAGCGACTGGGG
                                    *************** ******   * **** **

Epibacterium_ulvae_S003278975       --
Shimia_marina_S000515801            TG

======================================================================================
CLUSTAL W (1.81) multiple sequence alignment

Pseudoalteromonas_mariniglutinos      --------------------------------------------CACATGCAAGTCGAGC
Pseudoalteromonas_arabiensis_S00      -GAGTTTGATCCTGGCTCAGATTGAACGCTGGCGGCAGGCCTAACACATGCAAGTCGAGC
Pseudoalteromonas_lipolytica_S00      AGAGTTTGATCCTGGCTCAGATTGAACGCTGGCGGCAGGCCTAACACATGCAAGTCGAGC
Pseudoalteromonas_donghaensis_S0      AGAGTTTGATCATGGCTCAGATTGAACGCTGGCGGCAGGCCTAACACATGCAAGTCGAGC
                                                                                  ****************

Pseudoalteromonas_mariniglutinos      GGTAACATTTC-TAGCTTGCTA-GAAGATGACGAGCGGCGGACGGGTGAGTAATGCTTGG
Pseudoalteromonas_arabiensis_S00      GGAAACGAAGAGGAGCTTGCTCCTTTGGCGTCGAGCGGCGGACGGGTGAGTAATGCTTGG
Pseudoalteromonas_lipolytica_S00      GGTAACAGAGAGTAGCTTGCTACTCTGCTGACGAGCGGCGGACGGGTGAGTAATGCTTGG
Pseudoalteromonas_donghaensis_S0      GGTAACAGAGAGTAGCTTGCTACTCTGCTGACGAGCGGCGGACGGGTGAGTAATGCTTGG
                                      ** ***       ********     *  * *****************************

Pseudoalteromonas_mariniglutinos      GAATATGCCTTTTGGTGGGGGACAACAGTTGGAAACGACTGCTAATACCGCATGATGTCT
Pseudoalteromonas_arabiensis_S00      GAATGTGCCTTATGGTGGGGGACAACAGTTGGAAACGACTGCTAATACCGCATAATGTCT
Pseudoalteromonas_lipolytica_S00      GAATGTACCTTATGGTGGGGGACAACAGTTGGAAACGACTGCTAATACCGCATAATGTCT
Pseudoalteromonas_donghaensis_S0      GAATGTACCTTATGGTGGGGGACAACAGTTGGAAACGACTGCTAATACCGCATAATGTCT
                                      **** * **** ***************************************** ******

Pseudoalteromonas_mariniglutinos      ACGGACCAAAGTGGGGGACCTTCGGGCCTCACGCCAAAAGATTAGCCCAAGTGGGATTAG
Pseudoalteromonas_arabiensis_S00      TCGGACCAAAGTGGGGGACCTTCGGGCCTCACGCCATAAGATCAGCCCAAGTGGGATTAG
Pseudoalteromonas_lipolytica_S00      TCGGACCAAAGCGGGGGACCTTCGGGCCTCGCGCCATAAGATCAGCCCAAGTGGGATTAG
```

```
Pseudoalteromonas_donghaensis_S0      TCGGACCAAAGCGGGGGACCTTCGGGCCTCGCGCCATAAGATCAGCCCAAGTGGGATTAG
                                       ********** ****************** ***** ***** *****************

Pseudoalteromonas_mariniglutinos      CTAGTTGGTAAGGTAATGGCTTACCAAGGCGACGATCCCTAGCTGGTTTGAGAGGATGAT
Pseudoalteromonas_arabiensis_S00      CTAGTTGGTAAGGTAATGGCTTACCAAGGCGACGATCCCTAGCTGGTTTGAGAGGATGAT
Pseudoalteromonas_lipolytica_S00      CTAGTTGGTGAGGTAATGGCTCACCAAGGCGACGATCCCTAGCTGGTTTGAGAGGATGAT
Pseudoalteromonas_donghaensis_S0      CTAGTTGGTGAGGTAATGGCTCACCAAGGCGACGATCCCTAGCTGGTTTGAGAGGATGAT
                                      ********* *********** **************************************

Pseudoalteromonas_mariniglutinos      CAGCCACACTGGGACTGAGACACGGCCCAGACTCCTACGGGAGGCAGCAGTGGGGAATAT
Pseudoalteromonas_arabiensis_S00      CAGCCACACTGGGACTGAGACACGGCCCAGACTCCTACGGGAGGCAGCAGTGGGGAATAT
Pseudoalteromonas_lipolytica_S00      CAGCCACACTGGGACTGAGACACGGCCCAGACTCCTACGGGAGGCAGCAGTGGGGAATAT
Pseudoalteromonas_donghaensis_S0      CAGCCACACTGGGACTGAGACACGGCCCAGACTCCTACGGGAGGCAGCAGTGGGGAATAT
                                      ************************************************************

Pseudoalteromonas_mariniglutinos      TGCACAATGGGCGCAAGCCTGATGCAGCCATGCCGCGTGTGTGAAGAAGGCCTTCGGGTT
Pseudoalteromonas_arabiensis_S00      TGCACAATGGGCGCAAGCCTGATGCAGCCATGCCGCGTGTGTGAAGAAGGCCTTCGGGTT
Pseudoalteromonas_lipolytica_S00      TGCACAATGGGCGCAAGCCTGATGCAGCCATGCCGCGTGTGTGAAGAAGGCCTTCGGGTT
Pseudoalteromonas_donghaensis_S0      TGCACAATGGGCGCAAGCCTGATGCAGCCATGCCGCGTGTGTGAAGAAGGCCTTCGGGTT
                                      ************************************************************

Pseudoalteromonas_mariniglutinos      GTAAAGCACTTTCAGTAAGGAGGAAAGGTTAAGTGTTAATAGCACTTAGCTGTGACGTTA
Pseudoalteromonas_arabiensis_S00      GTAAAGCACTTTCAGTAAGGAGGAAAGGTTAAGTGTTAATAGCACTTAGCTGTGACGTTA
Pseudoalteromonas_lipolytica_S00      GTAAAGCACTTTCAGTAAGGAGGAAAGGTTGGATGTTAATAGCATTCAGCTGTGACGTTA
Pseudoalteromonas_donghaensis_S0      GTAAAGCACYTTCAGTAAGGAGGAAAGGTTGGATGTTAATAGCATTCAGCTGTGACGTTA
                                      ********* ********************   *********** * *************

Pseudoalteromonas_mariniglutinos      CTTACAGAAGAAGCACCGGCTAACTCCGTGCCAGCAGCCGCGGTAATACGGAGGGTGCGA
Pseudoalteromonas_arabiensis_S00      CTTACAGAAGAAGCACCGGCTAACTCCGTGCCAGCAGCCGCGGTAATACGGAGGGTGCGA
Pseudoalteromonas_lipolytica_S00      CTTACAGAAGAAGCACCGGCTAACTCCGTGCCAGCAGCCGCGGTAATACGGAGGGTGCGA
Pseudoalteromonas_donghaensis_S0      CTTACAGAAGAAGCACCGGCTAACTCCGTGCCAGCAGCCGCSGTAATACGGAGGGTGCGA
                                      ***************************************** ******************

Pseudoalteromonas_mariniglutinos      GCGTTAATCGGAATTACTGGGCGTAAAGCGTACGCAGGCGGTTTGTTAAGCGAGATGTGA
Pseudoalteromonas_arabiensis_S00      GCGTTAATCGGAATTACTGGGCGTAAAGCGTACGCAGGCGGTTTGTTAAGCGAGATGTGA
Pseudoalteromonas_lipolytica_S00      GCGTTAATCGGAATTACTGGGCGTAAAGCGTACGCAGGCGGTTTGTTAAGCGAGATGTGA
Pseudoalteromonas_donghaensis_S0      GCGTTAATCGGAATTACTGGGCGTAAAGCGTACGCAGGCGGTTTGTTAAGCGAGATGTGA
                                      ************************************************************

Pseudoalteromonas_mariniglutinos      AAGCCCCGGGCTCAACCTGGGAACTGCATTTCGAACTGGCAAACTAGAGTGTGATAGAGG
Pseudoalteromonas_arabiensis_S00      AAGCCCCGGGCTCAACCTGGGAACTGCATTTCGAACTGGCAAACTAGAGTGTGATAGAGG
Pseudoalteromonas_lipolytica_S00      AAGCCCCGGGCTCAACCTGGGAACTGCATTTCGAACTGGCAAACTAGAGTGTGATAGAGG
Pseudoalteromonas_donghaensis_S0      AAGCCCCGGGCTCAACCTGGGAACTGCATTTCGAACTGGCAAACTAGAGTGTGATAGAGG
```

```
                                             ************************************************************

Pseudoalteromonas_mariniglutinos             GTGGTAGAATTTCAGGTGTAGCGGTGAAATGCGTAGAGATCTGAAGGAATACCGATGGCG
Pseudoalteromonas_arabiensis_S00             GTGGTAGAATTTCAGGTGTAGCGGTGAAATGCGTAGAGATCTGAAGGAATACCGATGGCG
Pseudoalteromonas_lipolytica_S00             GTGGTAGAATTTCAGGTGTAGCGGTGAAATGCGTAGAGATCTGAAGGAATACCGATGGCG
Pseudoalteromonas_donghaensis_S0             GTGGTAGAATTTCAGGTGTAGCGGTGAAATGCGTAGAGATCTGAAGGAATACCGATGGCG
                                             ************************************************************

Pseudoalteromonas_mariniglutinos             AAGGCAGCCACCTGGGTCAACACTGACGCTCATGTACGAAAGCGTGGGGAGCAAACAGGA
Pseudoalteromonas_arabiensis_S00             AAGGCAGCCACCTGGGTCAACACTGACGCTCATGTACGAAAGCGTGGGGAGCAAACAGGA
Pseudoalteromonas_lipolytica_S00             AAGGCAGCCACCTGGGTCAACACTGACGCTCATGTACGAAAGCGTGGGGAGCAAACAGGA
Pseudoalteromonas_donghaensis_S0             AAGGCAGCCACCTGGGTCAACACTGACGCTCATGTACGAAAGCGTGGGGAGCAAACAGGA
                                             ************************************************************

Pseudoalteromonas_mariniglutinos             TTAGATACCCTGGTAGTCCACGCCGTAAACGATGTCTACTAGAAGCTCGGTTCCTCGGAA
Pseudoalteromonas_arabiensis_S00             TTAGATACCCTGGTAGTCCACGCCGTAAACGATGTCTACTAGAAGCTCGACTCTTCGGAG
Pseudoalteromonas_lipolytica_S00             TTAGATACCCTGGTAGTCCACGCCGTAAACGATGTCTACTAGAAGCTCGGTTCCTCGGAA
Pseudoalteromonas_donghaensis_S0             TTAGATACCCTGGTAGTCCACGCCGTAAACGATGTCTACTAGAAGCTCGGTTCCTCGGAA
                                             *************************************************  ** ***** 

Pseudoalteromonas_mariniglutinos             CTGTTTTTCAAAGCTAACGCATTAAGTAGACCGCCTGGGGAGTACGGCCGCAAGGTTAAA
Pseudoalteromonas_arabiensis_S00             TTGTTTTTCAAAGCTAACGCATTAAGTAGACCGCCTGGGGAGTACGGCCGCAAGGTTAAA
Pseudoalteromonas_lipolytica_S00             CTGTTTTTCAAAGCTAACGCATTAAGTAGACCGCCTGGGGAGTACGGCCGCAAGGTTAAA
Pseudoalteromonas_donghaensis_S0             CTGTTTTTCAAAGCTAACGCATTAAGTAGACCGCCTGGGGAGTACGGCCGCAAGGTTAAA
                                              ***********************************************************

Pseudoalteromonas_mariniglutinos             ACTCAAATGAATTGACGGGGGCCCGCACAAGCGGTGGAGCATGTGGTTTAATTCGATGCA
Pseudoalteromonas_arabiensis_S00             ACTCAAATGAATTGACGGGGGCCCGCACAAGCGGTGGAGCATGTGGTTTAATTCGATGCA
Pseudoalteromonas_lipolytica_S00             ACTCAAATGAATTGACGGGGGCCCGCACAAGCGGTGGAGCATGTGGTTTAATTCGATGCA
Pseudoalteromonas_donghaensis_S0             ACTCAAATGAATTGACGGGGGCCCGCACAAGCGGTGGAGCATGTGGTTTAATTCGATGCA
                                             ************************************************************

Pseudoalteromonas_mariniglutinos             ACGCGAAGAACCTTACCTACACTTGACATACAGAGAACTTACTAGAGATGGTTTGGTGCC
Pseudoalteromonas_arabiensis_S00             ACGCGAAGAACCTTACCTACACTTGACATACAGCGAACTTACTAGAGATAGTTTGGTGCC
Pseudoalteromonas_lipolytica_S00             ACGCGAAGAACCTTACCTACACTTGACATACAGAGAACTTACCAGAGATGGTTTGGTGCC
Pseudoalteromonas_donghaensis_S0             ACGCGAAGAACCTTACCTACACTTGACATACAGAGAACTTACCAGAGATGGTTTGGTGCC
                                             ********************************* ******** ****** **********

Pseudoalteromonas_mariniglutinos             TTCGGGAACTCTGATACAGGTGCTGCATGGCTGTCGTCAGCTCGTGTTGTGAGATGTTGG
Pseudoalteromonas_arabiensis_S00             TTCGGGAACGCTGATACAGGTGCTGCATGGCTGTCGTCAGCTCGTGTTGTGAGATGTTGG
Pseudoalteromonas_lipolytica_S00             TTCGGGAACTCTGATACAGGTGCTGCATGGCTGTCGTCAGCTCGTGTTGTGAGATGTTGG
Pseudoalteromonas_donghaensis_S0             TTCGGGAACTCTGATACAGGTGCTGCATGGCTGTCGTCAGCTCGTGTTGTGARATGTTGG
                                             ********* ****************************************** *******
```

```
Pseudoalteromonas_mariniglutinos      GTTAAGTCCCGCAACGAGCGCAACCCCTATCCTTAGTTGCTAGCAGGTAATGCTGAGAAC
Pseudoalteromonas_arabiensis_S00      GTTAAGTCCCGCAACGAGCGCAACCCCTATCCTTAGTTGCCAGC-GATTCGGTCGGGAAC
Pseudoalteromonas_lipolytica_S00      GTTAAGTCCCGCAACGAGCGCAACCCCTATCCTTAGTTGCCAGC-GATTCGGTCGGGAAC
Pseudoalteromonas_donghaensis_S0      GTTAAGTCCCGCAACGAGCGCAACCCCTATCCTTAGTTGCTAGCAGGTAATGCTGAGAAC
                                      **************************************** *** * *   *  * ****

Pseudoalteromonas_mariniglutinos      TCTAAGGAGACTGCCGGTGATAAACCGGAGGAAGGTGGGGACGACGTCAAGTCATCATGG
Pseudoalteromonas_arabiensis_S00      TCTAAGGAGACTGCCGGTGATAAACCGGAGGAAGGTGGGGACGACGTCAAGTCATCATGG
Pseudoalteromonas_lipolytica_S00      TCTAAGGAGACTGCCGGTGATAAACCGGAGGAAGGTGGGGACGACGTCAAGTCATCATGG
Pseudoalteromonas_donghaensis_S0      TCTAAGGAGACTGCCGGTGATAAACCGGAGGAAGGTGGGGACGACGTCAAGTCATCATGG
                                      ************************************************************

Pseudoalteromonas_mariniglutinos      CCCTTACGTGTAGGGCTACACACGTGCTACAATGGCGCATACAGAGTGCTGCGAACTCGC
Pseudoalteromonas_arabiensis_S00      CCCTTACGTGTAGGGCTACACACGTGCTACAATGGCGCATACAGAGTGCTGCGAACCTGC
Pseudoalteromonas_lipolytica_S00      CCCTTACGTGTAGGGCTACACACGTGCTACAATGGCGCATACAGAGTGCTGCGAACCTGC
Pseudoalteromonas_donghaensis_S0      CCCTTACGTGTAGGGCTACACACGTGCTACAATGGCGCATACAGAGTGCTGCGAACCTGC
                                      ********************************************************  **

Pseudoalteromonas_mariniglutinos      GAGAGTAAGCGAATCACTTAAAGTGCGTCGTAGTCCGGATTGGAGTCTGCAACTCGACTC
Pseudoalteromonas_arabiensis_S00      GAGGGTAAGCGAATCACTTAAAGTGCGTCGTAGTCCGGATTGGAGTCTGCAACTCGACTC
Pseudoalteromonas_lipolytica_S00      GAGGGTAAGCGAATCACTTAAAGTGCGTCGTAGTCCGGATTGGAGTCTGCAACTCGACTC
Pseudoalteromonas_donghaensis_S0      GAGGGTAAGCGAATCACTTAAAGTGCGTCGTAGTCCGGATTGGAGTCTGCAACTCGACTC
                                      *** ********************************************************

Pseudoalteromonas_mariniglutinos      CATGAAGTCGGAATCGCTAGTAATCGCGTATCAGAATGACGCGGTGAATACGTTCCCGGG
Pseudoalteromonas_arabiensis_S00      CATGAAGTCGGAATCGCTAGTAATCGCATATCAGAATGATGCGGTGAATACGTTCCCGGG
Pseudoalteromonas_lipolytica_S00      CATGAAGTCGGAATCGCTAGTAATCGCGTATCAGAATGACGCGGTGAATACGTTCCCGGG
Pseudoalteromonas_donghaensis_S0      CATGAAGTCGGAATCGCTAGTAATCGCGTATCAGAATGACGCGGTGAATACATTCCCGGG
                                      *************************** *********** *********** ********

Pseudoalteromonas_mariniglutinos      CCTTGTACACACCGCCCGTCACACCATGGGAGTGGGTTGCTCCAGAAGTAGATAGTCTAA
Pseudoalteromonas_arabiensis_S00      CCTTGTACACACCGCCCGTCACACCATGGGAGTGGGTTGCTCCAGAAGTGGATAGTCTAA
Pseudoalteromonas_lipolytica_S00      CCTTGTACACACCGCCCGTCACACCATGGGAGTGGGTTGCTCCAGAAGTGGATAGTCTAA
Pseudoalteromonas_donghaensis_S0      CCTTGTACACACCGCCCGTCACACCATGGGAGTGGGTTGCTCCAGAAGTGGATAGTCTAA
                                      ************************************************* **********

Pseudoalteromonas_mariniglutinos      CCCTCGGGAGGACGTTTACCACGGAGTGATTCATGACTGGGGTGAAGTCGTAACAAGGTA
Pseudoalteromonas_arabiensis_S00      CCTTCGGGAGGACGTTCACCACGGAGTGATTCATGACTGGGGTGAAGTCGTAACAAGGTA
Pseudoalteromonas_lipolytica_S00      CCTTCGGGAGGACGTTCACCACGGAGTGATTCATGACTGGGGTGAAGTCGTAACAAGGTA
Pseudoalteromonas_donghaensis_S0      CCTTCGGGAGGACGTTCACCACGGAGTGATTCATNANNGGGGTGAAGTCGTAACAAGGTA
                                      ** ************ ***************** *  ***********************
```

```
Pseudoalteromonas_mariniglutinos      GCCCTAGGGGAACCTGGGGCTGGATCACT
Pseudoalteromonas_arabiensis_S00      GCC--------------------------
Pseudoalteromonas_lipolytica_S00      ACCG-------------------------
Pseudoalteromonas_donghaensis_S0      ACCGTA-----------------------
                                       **

========================================================================================================
CLUSTAL W (1.81) multiple sequence alignment

Terrisporobacter_glycolicus_S000      -----------------------AACGCTGGCGGCGTGCCTAACACATGCAAGTCGAGCG
Terrisporobacter_mayombei_S00230      GAGTTTGATCCTGGCTCAGGATGAACGCTGGCGGCGTGCCTAACACATGCAAGTCGAGCG
                                                             *************************************

Terrisporobacter_glycolicus_S000      ATTCACTTCGGTGAAGAGCGGCGGACGGGTGAGTAACGCGTGGGTAACCTGCCTCATACA
Terrisporobacter_mayombei_S00230      ATTCTCTTCGGAGAAGAGCGGCGGACGGGTGAGTAACGCGTGGGTAACCTGCCTCATACA
                                      **** ****** ************************************************

Terrisporobacter_glycolicus_S000      CATGGATAACATACCGAAAGGTATGCTAATACAGGATAATATAAGAGATTCACATGTATT
Terrisporobacter_mayombei_S00230      CATGGATAACATACCGAAAGGTATGCTAATACAGGATGATATAAGAGATTCACATGGATT
                                      ************************************* ****************** ***

Terrisporobacter_glycolicus_S000      TCTTATCAAAGCTCCGGCGGTATGAGATGGACCCGCGTCTGATTAGCTAGTTGGTAAGGT
Terrisporobacter_mayombei_S00230      TCTTATCAAAGCTCCGGCGGTATGAGATGGACCCGCGTCTGATTAGCTAGTTGGTAAGGT
                                      ************************************************************

Terrisporobacter_glycolicus_S000      AACGGCTTACCAAGGCGACGATCAGTAGCCGACCTGAGAGGGTGATCGGCCACATTGGAA
Terrisporobacter_mayombei_S00230      AATGGCTTACCAAGGCGACGATCAGTAGCCGACCTGAGAGGGTGATCGGCCACATTGGAA
                                      ** *********************************************************

Terrisporobacter_glycolicus_S000      CTGAGACACGGTCCAAACTCCTACGGGAGGCAGCAGTGGGGAATATTGCACAATGGGCGA
Terrisporobacter_mayombei_S00230      CTGAGACACGGTCCAAACTCCTACGGGAGGCAGCAGTGGGGAATATTGCACAATGGGCGA
                                      ************************************************************

Terrisporobacter_glycolicus_S000      AAGCCTGATGCAGCAACGCCGCGTGAGTGATGAAGGCCTTCGGGTCGTAAAACTCTGTCC
Terrisporobacter_mayombei_S00230      AAGCCTGATGCAGCAACGCCGCGTGAGTGATGAAGGCCTTCGGGTCGTAAAACTCTGTCC
                                      ************************************************************

Terrisporobacter_glycolicus_S000      TCAAGGAAGATAATGACGGTACTTGAGGAGGAAGCCCCGGCTAACTACGTGCCAGCAGCC
Terrisporobacter_mayombei_S00230      TCAAGGAAGATAATGACGGTACTTGAGGAGGAAGCCCCGGCTAACTACGTGCCAGCAGCC
                                      ************************************************************

Terrisporobacter_glycolicus_S000      GCGGTAATACGTAGGGGGCTAGCGTTATCCGGATTTACTGGGCGTAAAGGGTGCGTAGGT
Terrisporobacter_mayombei_S00230      GCGGTAATACGTAGGGGGCTAGCGTTATCCGGATTTACTGGGCGTAAAGGGTGCGTAGGT
```

```
                                          ************************************************************

Terrisporobacter_glycolicus_S000          GGTTTCTTAAGTCAGGAGTGAAAGGCTACGGCTCAACCGTAGTAAGCTCTTGAAACTGGG
Terrisporobacter_mayombei_S00230          GGTTTCTTAAGTCAGGAGTGAAAGGCTACGGCTCAACCGTAGTAAGCTCTTGAAACTGGG
                                          ************************************************************

Terrisporobacter_glycolicus_S000          AGACTTGAGTGCAGGAGAGGAAAGTGGAATTCCTAGTGTAGCGGTGAAATGCGTAGATAT
Terrisporobacter_mayombei_S00230          AGACTTGAGTGCAGGAGAGGAAAGTGGAATTCCTAGTGTAGCGGTGAAATGCGTAGATAT
                                          ************************************************************

Terrisporobacter_glycolicus_S000          TAGGAGGAACACCAGTAGCGAAGGCGGCTTTCTGGACTGTAACTGACACTGAGGCACGAA
Terrisporobacter_mayombei_S00230          TAGGAGGAACACCAGTAGCGAAGGCGGCTTTCTGGACTGTAACTGACACTGAGGCACGAA
                                          ************************************************************

Terrisporobacter_glycolicus_S000          AGCGTGGGGAGCGAACAGGATTAGATACCCTGGTAGTCCACGCCGTAAACGATGAGTACT
Terrisporobacter_mayombei_S00230          AGCGTGGGGAGCGAACAGGATTAGATACCCTGGTAGTCCACGCCGTAAACGATGAGTACT
                                          ************************************************************

Terrisporobacter_glycolicus_S000          AGGTGTCGGGGGTTACCCCCCTCGGTGCCGCACGTAACGCATTAAGTACTCCGCCTGGGG
Terrisporobacter_mayombei_S00230          AGGTGTCGGGGGTTACCCCCCTCGGTGCCGCAGCTAACGCATTAAGTACTCCGCCTGGGG
                                          ********************************  **************************

Terrisporobacter_glycolicus_S000          AGTACGCTCGCAAGAGTGAAACTCAAAGGAATTGACGGGGACCCGCACAAGTAGCGGAGC
Terrisporobacter_mayombei_S00230          AGTACGCTCGCAAGAGTGAAACTCAAAGGAATTGACGGGGACCCGCACAAGTAGCGGAGC
                                          ************************************************************

Terrisporobacter_glycolicus_S000          ATGTGGTTTAATTCGAAGCAACGCGAAGAACCTTACCTAAGCTTGACATCCTTTTGACCG
Terrisporobacter_mayombei_S00230          ATGTGGTTTAATTCGAAGCAACGCGAAGAACCTTACCTAAGCTTGACATCCTTTTGACCG
                                          ************************************************************

Terrisporobacter_glycolicus_S000          ATGCCTAATCGCATTTTTCCCTTCGGGGACAGAAGTGACAGGTGGTGCATGGTTGTCGTC
Terrisporobacter_mayombei_S00230          ATGCCTAATCGCATCTTTCCCTTCGGGGACAGAAGTGACAGGTGGTGCATGGTTGTCGTC
                                          ************** *********************************************

Terrisporobacter_glycolicus_S000          AGCTCGTGTCGTGAGATGTTGGGTTAAGTCCCGCAACGAGCGCAACCCTTGCCTTTAGTT
Terrisporobacter_mayombei_S00230          AGCTCGTGTCGTGAGATGTTGGGTTAAGTCCCGCAACGAGCGCAACCCTTGCCTTTAGTT
                                          ************************************************************

Terrisporobacter_glycolicus_S000          GCCAGCATTAAGTTGGGCACTCTAGAGGGACTGCCAGGGATAACCTGGAGGAAGGTGGGG
Terrisporobacter_mayombei_S00230          GCCAGCATTAAGTTGGGCACTCTAGAGGGACTGCCAGGGATAACCTGGAGGAAGGTGGGG
                                          ************************************************************

Terrisporobacter_glycolicus_S000          ATGACGTCAAATCATCATGCCCCTTATGCTTAGGGCTACACACGTGCTACAATGGGTGGT
```

```
Terrisporobacter_mayombei_S00230      ATGACGTCAAATCATCATGCCCCTTATGCTTAGGGCTACACACGTGCTACAATGGGTGGT
                                      ************************************************************

Terrisporobacter_glycolicus_S000      ACAGAGGGCAGCCAAGTCGTGAGGCCGAGCTAATCCCTTAAATGCCATTCTCAGTTCGGA
Terrisporobacter_mayombei_S00230      ACAGAGGGCAGCCAAGTCGTGAGGCCGAGCTAATCCCTTAAA-GCCATTCTCAGTTCGGA
                                      ****************************************** *****************

Terrisporobacter_glycolicus_S000      TTGTAGGCTGAAACTCGCCTACATGAAGCTGGAGTTACTAGTAATCGCAGATCAGAATGC
Terrisporobacter_mayombei_S00230      TTGTAGGCTGAAACTCGCCTACATGAAGCTGGAGTTACTAGTAATCGCAGATCAGAATGC
                                      ************************************************************

Terrisporobacter_glycolicus_S000      TGCGGTGAATGCGTTCCCGGGTCTTGTACACACCGCCCGTCACACCACGGAAGTTGGGGG
Terrisporobacter_mayombei_S00230      TGCGGTGAATGCGTTCCCGGGTCTTGTACACACCGCCCGTCACACCACGGAAGTTGGGGG
                                      ************************************************************

Terrisporobacter_glycolicus_S000      CGCCCGAAGCCACTTAGCTAACCCTTTTGGGAAGCGAGTGTCGAAGGTGAAATCAATAAC
Terrisporobacter_mayombei_S00230      CGCCCGAAGCCACTTAGCTAACCTTTT--GGAAGCGAGTGTCGAAGGTGAAATCAATAAC
                                      *********************** ***  *******************************


Terrisporobacter_glycolicus_S000      TGGGGTGAAGTCGTAACAAGGTAGCCGTATCGGAAGGTGCGGCT-------------
Terrisporobacter_mayombei_S00230      TGGGGTGAAGTCGTAACAAGGTAGCCGTATCGGAAGGTGCGGCTGGATCACCTCCTT
                                      ********************************************
==================================================================================================

CLUSTAL W (1.81) multiple sequence alignment

Vallitalea_guaymasensis_S0032878      AGTTT-GATCCTGGCTCAGGATGAACGCTGGCGGCGTGCTTAACACATGCAAGTCGAGCG
Vallitalea_pronyensis_S003804798      AGTTTGGATCCTGGCTCAGGATGAACGCTGGCGGCGTGCTTAACACATGCAAGTCGAGCG
                                      ***** ******************************************************

Vallitalea_guaymasensis_S0032878      AGAAGCTATCTATTGAACCTTCGGGTGATTTAGATAGTGGAAAGCGGCGGACGGGTGAGT
Vallitalea_pronyensis_S003804798      AGAAACTTAAGGATGATCCTTCGGGTGATTCCTTAAGTGGACAGCGGCGGACGGGTGAGT
                                      **** **      *** *************     ****** ******************

Vallitalea_guaymasensis_S0032878      AACGCGTGGGTAACCTGCCCTATGCAGGGGGATAACACATTGAAAAGTGTGCTAATACCG
Vallitalea_pronyensis_S003804798      AACGCGTGGGTAACCTGCCCTATGCAGGGGGATAACGCACTGAAAAGTGCGCTAATACCG
                                      ************************************ ** ********* **********

Vallitalea_guaymasensis_S0032878      CATAAGACCACAGAGTCGCATGACTCAGTGGTAAAAACTCCGGTAGCATAGGATGGACCC
Vallitalea_pronyensis_S003804798      CATAAGACCACAGGATCACATGATCTGGTGGTAAAAACTCCGGTGGCATAGGATGGACCC
                                      *************  ** *****    ***************** ***************
```

```
Vallitalea_guaymasensis_S0032878      GCGTCTGATTAGCTAGTAGGTAAGGTAACGGCTTACCTAGGCGACGATCAGTAGCCGACC
Vallitalea_pronyensis_S003804798      GCGTCTGATTAGCTAGTAGGTGAGGTAACGGCTCACCTAGGCGACGATCAGTAGCCGACC
                                      ********************* *********** **************************

Vallitalea_guaymasensis_S0032878      TGAGAGGGTGATCGGCCACATTGGGACTGAGACACGGCCCAAACTCCTACGGGAGGCAGC
Vallitalea_pronyensis_S003804798      TGAGAGGGTGATCGGCCACATTGGGACTGAGACACGGCCCAAACTCCTACGGGAGGCAGC
                                      ************************************************************

Vallitalea_guaymasensis_S0032878      AGTGGGGAATATTGCACAATGGGGGAAACCCTGATGCAGCGACGCCGCGTGAAGGAAGAA
Vallitalea_pronyensis_S003804798      AGTGGGGAATATTGCACAATGGGGGAAACCCTGATGCAGCGACGCCGCGTGAAGGAAGAA
                                      ************************************************************

Vallitalea_guaymasensis_S0032878      GGTTTTCGGATCGTAAACTTCTATCAGCAGGGAAGATAGTGACAGTACCTGACTAAGAAG
Vallitalea_pronyensis_S003804798      GGTTTTCGGATCGTAAACTTCTATCAGCAGGGAAGATAGTGACAGTACCTGACTAAGAAG
                                      ************************************************************

Vallitalea_guaymasensis_S0032878      CCCCGGCTAACTACGTGCCAGCAGCCGCGGTAATACGTAGGGGGCAAGCGTTATCCGGAT
Vallitalea_pronyensis_S003804798      CCCCGGCTAACTACGTGCCAGCAGCCGCGGTAATACGTAGGGGGCAAGCGTTATCCGGAT
                                      ************************************************************

Vallitalea_guaymasensis_S0032878      TTACTGGGTGTAAAGGGTGCGTAGGCGGCGAAGTAAGTCAGATGTGAAAGCCCGAGGCTC
Vallitalea_pronyensis_S003804798      TTACTGGGTGTAAAGGGTGCGTAGGCGGCGGAGTAAGTCAGATGTGAAAGCCCCAGGCTC
                                      ****************************** ********************** ******

Vallitalea_guaymasensis_S0032878      AACCTCGGGACTGCATTTGAAACTGCTTTGCTAGAGTGCAGGAGAGGAAAGCGGAATTCC
Vallitalea_pronyensis_S003804798      AACCTGGGGACTGCATTTGAAACTGCTTTGCTAGAGTGCAGGAGAGGAAAGTGGAATTCC
                                      ***** ********************************************* ********

Vallitalea_guaymasensis_S0032878      TAGTGTAGCGGTGAAATGCGTAGATATTAGGAGGAACACCAGTGGCGAAGGCGGCTTTCT
Vallitalea_pronyensis_S003804798      TAGTGTAGCGGTGAAATGCGTAGATATTAGGAGGAACACCAGTGGCGAAGGCGACTTTCT
                                      ****************************************************** *****

Vallitalea_guaymasensis_S0032878      GGACTGTAACTGACGCTGAGGCACGAAAGCGTGGGGAGCGAACAGGATTAGATACCCTGG
Vallitalea_pronyensis_S003804798      GGACTGTAACTGACGCTGAGGCACGAAAGCGTGGGGAGCGAACAGGATTAGATACCCTGG
                                      ************************************************************

Vallitalea_guaymasensis_S0032878      TAGTCCACGCCGTAAACGATGAATGCTAGGTGTCGGGGGTCGAACCTCGGTGCCGCAGCA
Vallitalea_pronyensis_S003804798      TAGTCCACGCCGTAAACGATGAATGCTAGGTGTCGGGGGTCGAACCTCGGTGCCGCAGCA
                                      ************************************************************

Vallitalea_guaymasensis_S0032878      AACGCATTAAGCATTCCACCTGGGGAGTACGATCGCAAGATTGAAACTCAAAGGAATTGA
Vallitalea_pronyensis_S003804798      AACGCATTAAGCATTCCACCTGGGGAGTACGATCGCAAGATTGAAACTCAAAGGAATTGA
```

```
                                   ************************************************************

Vallitalea_guaymasensis_S0032878      CGGGGGCCCGCACAAGCGGTGGAGCATGTGGTTTAATTCGAAGCAACGCGAAGAACCTTA
Vallitalea_pronyensis_S003804798      CGGGGGCCCGCACAAGCGGTGGAGCATGTGGTTTAATTCGAAGCAACGCGAAGAACCTTA
                                      ************************************************************

Vallitalea_guaymasensis_S0032878      CCAAATCTTGACATCCTTCTGACCGTTCCTTAATCGGAACTTTCCTTCGGGACAGAAGAG
Vallitalea_pronyensis_S003804798      CCAAATCTTGACATCCTTCTGACCGTTCCTTAATCGGAACTTTCCTTCGGGACAGAAGAG
                                      ************************************************************

Vallitalea_guaymasensis_S0032878      ACAGGTGGTGCATGGTTGTCGTCAGCTCGTGTCGTGAGATGTTGGGTTAAGTCCCGCAAC
Vallitalea_pronyensis_S003804798      ACAGGTGGTGCATGGTTGTCGTCAGCTCGTGTCGTGAGATGTTGGGTTAAGTCCCGCAAC
                                      ************************************************************

Vallitalea_guaymasensis_S0032878      GAGCGCAACCCTTATCTTTAGTAGCCAGCAAGTTAAGTTGGGGACTCTAGAGAGACTGCC
Vallitalea_pronyensis_S003804798      GAGCGCAACCCTTATCTTTAGTAGCCAGCAAGTAAAGTTGGGGACTCTAGAGAGACTGCC
                                      ********************************* **************************

Vallitalea_guaymasensis_S0032878      GGGGACAACTCGGAGGAAGGTGGGGATGACGTCAAATCATCATGCCCCTTATGATTTGGG
Vallitalea_pronyensis_S003804798      GGGGACAACTCGGAGGAAGGTGGGGATGACGTCAAATCATCATGCCCCTTATGATTTGGG
                                      ************************************************************

Vallitalea_guaymasensis_S0032878      CTACACACGTGCTACAATGGCGGTGACAAAGGGAAGCAAAATGGTGACATGGAGCAAATC
Vallitalea_pronyensis_S003804798      CTACACACGTGCTACAATGGTAGTGACAAAGGGAAGCGAAGTGGTGACATGGAGCCAACC
                                      ********************  *************** ** ************** ** *

Vallitalea_guaymasensis_S0032878      CCAAAAAAGCCGTCCCAGTTCGGATTGTAGTCTGCAACTCGACTACATGAAGTTGGAATC
Vallitalea_pronyensis_S003804798      CCAAAAAAGCTATCCCAGTTCGGATTGTAGTCTGCAACTCGACTACATGAAGTTGGAATC
                                      **********  ************************************************

Vallitalea_guaymasensis_S0032878      GCTAGTAATCGCGAATCAGAATGTCGCGGTGAATACGTTCCCGGGCCTTGTACACACCGC
Vallitalea_pronyensis_S003804798      GCTAGTAATCGCGAATCAGCATGTCGCGGTGAATACGTTCCCGGGCCTTGTACACACCGC
                                      ******************* ****************************************

Vallitalea_guaymasensis_S0032878      CCGTCACACCATGGGAG-TGGAAGCGCCCGAAGCCAG-GACTCAAC--GCAAAGAGAGAG
Vallitalea_pronyensis_S003804798      CCGTCACACCACGGGAGTTGGAAGCGCCCGAAGCCAGTGACCAAACCTTTATGGATGGAG
                                      *********** ***** ******************* ***  ***    *  **  ***

Vallitalea_guaymasensis_S0032878      CT-TCGAAGG------------------------------
Vallitalea_pronyensis_S003804798      CTGTCCAAGGTGAAGCCGATGACTGGGGTGAAGTCGTAAC
                                      ** ** ****
```
